# Mouse Stromal Cells Confound Proteomic Characterization and Quantification of Xenograft Models

**DOI:** 10.1158/2767-9764.CRC-22-0431

**Published:** 2023-02-06

**Authors:** Zhaomei Shi, Binchen Mao, Xiaobo Chen, Piliang Hao, Sheng Guo

**Affiliations:** 1 School of Life Science and Technology, ShanghaiTech University, Shanghai, P.R. China.; 2 Crown Bioscience Inc., Suzhou, P.R. China.

## Abstract

Xenografts are essential models for studying cancer biology and developing oncology drugs, and are more informative with omics data. Most reported xenograft proteomics projects directly profiled tumors comprising human cancer cells and mouse stromal cells, followed by computational algorithms for assigning peptides to human and mouse proteins. We evaluated the performance of three main algorithms by carrying out benchmark studies on a series of human and mouse cell line mixtures and a set of liver patient-derived xenograft (PDX) models. Our study showed that approximately half of the characterized peptides are common between human and mouse proteins, and their allocations to human or mouse proteins cannot be satisfactorily achieved by any algorithm. As a result, many human proteins are erroneously labeled as differentially expressed proteins (DEP) between samples from the same human cell line mixed with different percentages of mouse cells, and the number of such false DEPs increases superquadratically with the mouse cell percentage. When mouse stromal cells are not removed from PDX tumors, about 30%–40% of DEPs from pairwise comparisons of PDX models are false positives, and about 20% of real DEPs cannot be identified irrespective of the threshold for calling differential expression. In conclusion, our study demonstrated that it is advisable to separate human and mouse cells in xenograft tumors before proteomic profiling to obtain more accurate measurement of species-specific protein expression.

**Significance:**

This study advocates the separate-then-run over the run-then-separate approach as a better strategy for more reliable proteomic profiling of xenografts.

## Introduction

Cancer research and treatment can be improved by accumulating and mining large-scale multi-omics data (1, 2). Proteomic profiling provides protein abundance information that complements genomics data from next-generation sequencing (NGS) and has been used in some large efforts, such as the Clinical Proteomic Tumor Analysis Consortium (CPTAC) project (3), to profile patient samples for understanding the molecular basis of cancer. In recent years, initiatives were started for proteomic characterization of xenograft models (4–6), a primary workhorse for preclinical cancer research and drug development. Xenograft models are established by implanting either cancer cells or patient tumors into immunodeficient mice to form tumors. Cell line–derived xenografts have the advantages of fast model development, highly reproducible efficacy, and convenience for genetic engineering of target genes. Patient-derived xenografts (PDX) maintain a high resemblance to patient tumors in molecular pathology, histology, genomics, and drug response (7–9).

However, proteomic profiling of xenografts faces a major challenge in distinguishing human and mouse proteins because human stroma is rapidly replaced by mouse stroma after initial tumor engraftment (10–12). Although there have been some prudent efforts to separate human and mouse cells before proteomic profiling (13), most studies performed species-specific peptide and protein separation and quantification after data acquisition using a variety of algorithms: (i) in the Full-peptide approach, both human-mouse common peptides and species-unique peptides are searched against a unified human and mouse protein database, common peptides are either assigned to a species or both or discarded (14); (ii) in the Unique-peptide approach, common peptides are discarded to allow unambiguous peptide assignment (15, 16); and (iii) in the Human/Mouse-only approach, peptides are searched against only the human or mouse protein database (6, 17–20). In practice, variants or hybrids of these algorithms were often used (4, 21–23). In all methods, allocating peptides to proteins is done in much the same way as assigning short nucleotide reads from NGS to human and mouse genes based on sequence similarity. Intuitively, this is much harder because proteins share higher sequence similarity than genes between the two species, so there are abundant human-mouse common peptides from cleaving proteins.

Here, we report the first benchmark study that comprehensively evaluates the performance of the three methods in characterizing and quantifying human protein expression in the presence of mouse proteins. Tandem mass tagging (TMT)-based quantitative proteomics technology was used for its high sensitivity, specificity, and multiplexing capacity to accommodate 16 samples. In the first part, we used a series of human-mouse cell mixing samples to show that many human proteins artificially exhibited differential expression between human cell samples containing different percentages of mouse cells. Furthermore, the number of seemingly differentially expressed proteins (DEP) increased quickly with the mouse cell percentage difference between samples. We then performed an in-depth analysis to demonstrate that such artifacts were largely caused by human-mouse common peptides, and the problem is unlikely to be resolved by computational methods. In the second part, we profiled a set of liver PDX models with varied mouse contents, each with an original xenograft tumor and its demoused pair. We showed that PDX tumors are very sensitive to mouse stroma in quantifying human protein expression. Many DEPs were falsely labeled and many real DEPs could not be identified in both intra-PDX and inter-PDX comparative studies.

## Materials and Methods

### PDX Model Growth and Tumor Excision

Tumor fragments from stock mice were harvested and inoculated into mice. Each mouse was inoculated subcutaneously in the right flank with a tumor fragment (2–3 mm in diameter) for tumor development. After tumor inoculation, the animal was monitored daily for morbidity and mortality. Tumor volume was measured twice per week in two dimensions using a caliper, and the volume was calculated using the formula *V* = (*L* × *W* × *W*)/2, where *V* is the tumor volume, *L* is the tumor length (the longest tumor dimension), and *W* is the tumor width (the longest tumor dimension perpendicular to *L*). The tumor was collected once its volume reached 1,000 mm^3^. All animal studies were done in the specific pathogen free animal facility at the institute with approved protocols by the Institutional Animal Care and Use Committee.

### Mouse Cell Removal for PDX Tumors

Five liver PDX models developed from unrelated patients with cancer were used in the study. Collected PDX tumors were dissociated into single-cell suspensions using the Human Tumor Dissociation Kit (Miltenyi Biotec, catalog no.130-095-929) in a gentle MACS Dissociator (Miltenyi Biotec). The number of cells was determined using Cellometer Auto T4 (Nexcelom Bioscience), and a total of 10 million cells (including red blood cells) were used for mouse cell removal following the manufacturer manual of Mouse Cell Depletion Kit (Miltenyi Biotec, catalog no. 130-104-694). Enriched human tumor cells were pooled for each PDX model and five million cell pellets per tube were frozen for mouse cell percentage analysis. All cell pellets were stored at −80°C.

### Measuring Mouse Cell Percentage by the NGS-QC Panel

Five million enriched human tumor cell pellets were used for the mouse content assay by using a deep NGS-QC (quality control) panel (24), which also checked *Mycoplasma* contamination with negative results. Genomic DNA from cells was extracted using MagMAX DNA Multi-Sample Ultra 2.0 (Thermo Fisher Scientific, catalog no. A36570) on a KingFisher Flex automatic workstation (Thermo Fisher Scientific) according to the manufacturer's instructions. DNA integrity was determined by the 2100 Bioanalyzer (Agilent) and quantified using NanoDrop (Thermo Fisher Scientific). One aliquot of a high-quality DNA sample (OD260/280 = 1.8–2.0, OD260/230 ≥ 2.0, >1 μg) was used for deep NGS. Multiplex PCR was used to prepare target sequencing libraries for an MGISEQ2000 sequencer with a paired-end read length of 150 bp (pE150). The NGS-QC panel contains two primer pools covering 678 amplicons (MGI, catalog no. 1000024929). Amplicon amplification and library preparation were performed on an MGISP960 (MGI) automatic workstation using the ATOPlex DNA Multiplex PCR Universal Library Construction Module (catalog no. 1000021191) and the ATOPlex Single Barcode Primer Module (catalog no. 1000024934). After library construction, the Qubit 3.0 Fluorometer dsDNA HS Assay (Thermo Fisher Scientific) was used to quantify the concentrations of the resulting sequencing libraries. The 2100 Bioanalyzer (Agilent) was used to analyze the size distribution, ranging from 280 to 420 bp. Paired-end sequencing was performed using an MGISEQ2000 system following the MGI-provided protocols for 2 × 150 bp paired-end sequencing.

### Sample Preparation of Cell Line Samples

The human cell line 273T and mouse cell line MC38 were seeded in 15 mL medium in T75 which allowed cell confluence to reach 80%–90%, followed by overnight incubation in a CO_2_ water-jacketed incubator (SANYO). After the medium was aspirated, the cells were washed with precooled D-PBS (Cellmax, catalog no. CBS101.05). The 0.25% trypsin-EDTA (Gibco, catalog no. 25200-072) was added to the attached cells and an appropriate volume of complete medium was added to stop trypsinization. The cells were then centrifuged at 3,000 rpm for 5 minutes. The supernatant was aspirated and the cell pellets were retained. Cell line MC38 was acquired from the Institutes of Biomedical Sciences of Fudan University (Shanghai, P.R. China), and 273T was purchased from ATCC. Both cell lines were authenticated by using a deep NGS-QC panel (24), which also checked mycoplasma contamination with negative results.

About 10 million human 293T cells and mouse MC38 cells were resuspended in 500 μL lysis buffer (8 mol/L urea, 50 mmol/L ammonium bicarbonate, 1 mmol/L dithiothreitol [DTT]) with protease inhibitor added (10 mL/tablet, 05892791001, Roche). The suspension was sonicated three times for 10 seconds on ice. The protein concentration of the lysates was determined using the bicinchoninic acid assay. Approximately 1 mg of lysate was reduced with 5 mmol/L DTT at 37°C for 2 hours and alkylated with 20 mmol/L iodoacetamide for 45 minutes in the dark. After the urea was diluted to 1 mol/L with 50 mmol/L ammonium bicarbonate (ABC), trypsin (V5111, Promega) was added at a weight ratio of 1:50. The cells were then incubated for 16 hours at 37°C. The reactions were stopped by adding 20% formic acid (FA) until a pH < 2 was reached. Peptides were desalted with MonoSpin C18 (5010-21701, Shimadzu-GL) according to the manufacturer's instructions and dried under vacuum conditions. Peptides from 293T cells and MC38 cells were dissolved in 100 mmol/L triethylammonium bicarbonate, mixed at specified ratios as shown in Table 1, and labeled with TMTpro-16plex according to the manufacturer's protocol. All 16 labeled samples were combined and dried under vacuum conditions. It was then desalted using a Sep-Pak C18 column (WAT054955, Waters) according to the manufacturer's instructions and dried in a vacuum. Hp-RP fractionation was done as previously described with slight modifications (25). Briefly, the labeled peptides were dissolved in 100 μL buffer A (2% acetonitrile [ACN], adjusted to pH 10 using ammonium hydroxide), injected completely with an autosampler, and fractionated using an XBridge C18 column (4.6 × 250 mm, 5 μm, 130 Å; Waters) on an Ultimate 3000 UPLC system monitored at 280 nm. Thirty-six fractions were collected with a 70-minute gradient of 5% buffer B (98% ACN, adjusted to pH 10 using ammonium hydroxide) for 2 minutes, 5%–15% B for 8 minutes, 15%–30% B for 20 minutes, 30%–45% B for 25 minutes, 45%–95% B for 5 minutes, 95% B for 8 minutes, followed by 95%–5% B for 2 minutes at a flow rate of 0.7 mL/minute. The fractions were then dried in a vacuum, pooled into 12 fractions as described previously (25), and redissolved in 0.1% FA for LC/MS-MS analysis.

**TABLE 1:** Benchmark study samples

16 TMTpro	293T and MC38 cell line mixture^a^	PDX tumor^b^	Mouse cell percentage in PDX tumor
126	100_1	LI6663A_1	7.8
127N	100_2	LI6663A_2	
127C	98_1	LI6650A_1	15.6
128N	98_2	LI6650A_2	
128C	95_1	LI6674A_1	51.3
129N	95_2	LI6674A_2	
129C	92_1	LI6675A	17.3
130N	92_2	LI1057A	90.7
130C	85_1	LI6663B_1	0.2
131N	85_2	LI6663B_2	
131C	75_1	LI6650B_1	0.3
132N	75_2	LI6650B_2	
132C	65_1	LI6674B_1	0.8
133N	65_2	LI6674B_2	
133C	55_1	LI6675B	0.4
134N	55_2	LI1057B	4.9

^a^The number before the underscore shows the percentage of human 293T cells in its mixture with mouse MC38 cells; the number after the underscore denotes the technical replicate of a sample.

^b^LI stands for liver cancer, LI6663 is a PDX model number, and “A” and “B” refer to tumor samples before and after mouse cell depletion, respectively. The number after the underscore denotes the technical replicate of the sample. The five PDX models were originated from unrelated patients.

### Sample Preparation of Xenograft Tumor Samples

The cells extracted from xenograft tumor samples LI1057, LI6650, LI6663, LI6674, and LI6675 before and after removing mouse cells were prepared in the same way as the cell samples. Peptides from these samples were labeled with TMTpro-16plex, according to the manufacturer's instructions, as shown in Table 1.

### Proteomics Data Generation and Analysis

Peptides were separated and analyzed on an Easy-nLC 1200 system coupled to a Q Exactive HF-X (Thermo Fisher Scientific). Approximately 1 μg of peptides was separated in an Easy-Spray column (75 μm × 50 cm, ES903, Thermo Fisher Scientific) at a flow rate of 250 nL/minute at 55°C. Mobile phase A (0.1% formic acid in 2% ACN) and mobile phase B (0.1% formic acid in 98% ACN) were used to establish a 120-minute gradient comprised of 102 minutes of 6%–31% B, 5 minutes of 31–38% B, 1 minute 38%–90% B, and 12 minutes of 90% B. Peptides were then ionized by electrospray at 2.1 kV. A full mass spectrometry (MS) spectrum (375–1,700 m/z range) was acquired at a resolution of 60,000 at m/z 200 and maximum ion accumulation time of 20 ms. Dynamic exclusion was set to 30 seconds. The resolution of the higher-energy collisional dissociation (HCD) MS-MS spectra was set to 30,000 at m/z 200. The AGC setting for MS1 and MS2 were set at 3E6 and 1E5, respectively. The 20 most intense ions above a 4E4 counts threshold were selected for fragmentation by HCD with a maximum ion accumulation time of 50 ms. MS2 isolation width of 1.2 m/z units was used. Single and unassigned charged ions were excluded from the MS-MS analysis. For HCD, the normalized collision energy was set to 32% (26).

The raw data were processed using Proteome Discoverer software (PD; version 2.2.0.388, Thermo Fisher Scientific) with default settings unless otherwise specified. The UniProt human protein database (release 2022_08, 79489 sequences) or the UniProt mouse database (release 2016_07, 49821) was used for protein searches of TMTpro-16plex labeled samples with proteomics contaminants from MaxQuant, and razor peptides were used for protein quantification. We designated these two search algorithms as Human-only and Mouse-only methods. In addition, the UniProt human and mouse protein databases were combined and used for protein search and quantification using only unique peptides (the Unique-peptide method) or by using both unique peptides and razor peptides (the Full-peptide method). Specifically, the two methods were chosen by setting the “The Peptides to Use” parameter to “Unique” and “Unique + Razor.” A unique peptide is mapped to only one protein or protein group, whereas a razor peptide is shared among multiple protein groups or proteins. Trypsin/P was set as the enzyme, and two missed cleavage sites of trypsin were allowed. The mass error was set to 10 ppm for precursor ions and 0.02 Da for fragment ions. Carbamidomethylation on Cys and TMTpro (+304.207 Da, K and peptide N-termini) was specified as a fixed modification; and oxidation (M), pyro-Glu (peptide N-term Q), and acetylation (Protein N-term) were set as variable modifications. The FDR thresholds for proteins and peptides were specified as 1%. The minimum peptide length was set to 7. All other parameters were set to their default values.

In all three methods, the sum of the human protein abundance was normalized to a constant value across all samples. To obtain thresholds for differential expression, the ratio of protein abundance was calculated for every protein between two technical replicates in two rounds. Each replicate was used as the denominator to avoid bias. The Kruskal–Wallis test was used to check the equality of medians among multiple groups, and Welch test was used to check the equality of means between two groups. All statistical analyses were performed using R software (version 4.2.1). Gene set enrichment analysis was performed online using the Enrichr platform (27).

### Data Availability

The MS proteomics data were deposited into the ProteomeXchange Consortium (http://proteomecentral.proteomexchange.org) via the iProX partner repository (28) with the dataset identifier PXD037070. The raw NGS-QC Panel sequencing data in FASTQ format for quantifying mouse ratios in 10 PDX tumor samples were deposited to the Sequence Read Archive with accession number PRJNA925686 (https://www.ncbi.nlm.nih.gov/sra/PRJNA925686).

## Results

### Sample Description

We prepared two sets of samples for the benchmark study (Table 1). The first was a series of human cell line 293T and mouse cell line MC38 mixtures, made by directly mixing peptides from individual cell lines via protein extraction and lysis. Eight mixing ratios were used, each with two technical replicates. The second was a collection of five liver PDX models, each with an original tumor excised from an immunodeficient mouse and the same tumor treated with a mouse cell depletion kit to remove mouse stromal cells. All tumor samples were homogenized for protein extraction and proteomic profiling. Several samples had two technical replicates. The percentage of mouse stromal cells in a PDX tumor sample was accurately measured using a deep-sequencing NGS assay (24). For simplicity, we use human/mouse cell percentage in both sets with the understanding that it refers to the percentage of human/mouse proteins in the cell line mixtures and the actual percentage of human/mouse cells in the PDX tumors. In addition, we note that 293T and MC38 are epithelial cells and the protein expression of MC38 does not recapitulate that of mouse stromal cells. Their mixtures were mainly used for peptide mapping and protein quantification.

### Human Protein Quantification in Human and Mouse Cell Line Mixtures

#### Overview of Protein Quantification Methods and Data Quality

We performed human protein quantification by searching against a combined human and mouse reference protein database using all mappable peptides, or using only unique peptides, or by searching against only a human reference protein database. We denote these three methods as Full-peptide, Unique-peptide, and Human-only, respectively. A unique peptide is unambiguously mapped to only one protein or protein group from which a master protein is used to represent the expression of multiple proteins with very high sequence similarity. In addition, we carried out a Mouse-only search against a mouse reference protein database. The Full-peptide and Unique-peptide methods quantified approximately twice as many human proteins as mouse proteins, which is unsurprising because the samples were made up of either purely or primarily human cells (Tables 1 and 2). The Human-only method ignored mouse content and returned approximately 1,000 more human proteins, many of which, as we will show later, primarily resulted from human-mouse common peptides (Table 2). All proteins were used in subsequent analyses even though their quantification had different confidence levels (Supplementary Table S1).

**TABLE 2:** Number of quantified proteins by four methods in 16 mixtures of human cell line 293T and mouse cell line MC38

	Full-peptide	Unique-peptide	Human-only	Mouse-only
Quantified human proteins	7,283 (6,831, 322, 130)^a^	7,277 (6,819, 326, 132)	8,174 (7,590, 461, 123)	0
Quantified mouse proteins	3,473 (3,218, 173, 82)	3,451 (3,195, 174, 82)	0	7,041 (6,260, 652, 129)

^a^Number of proteins with abundance quantification in all 16 samples; the three numbers in parentheses are the number of proteins with high, medium, and low quantification confidence (FDR < 0.01, 0.05, and 0.1, respectively).

**FIGURE 1:**
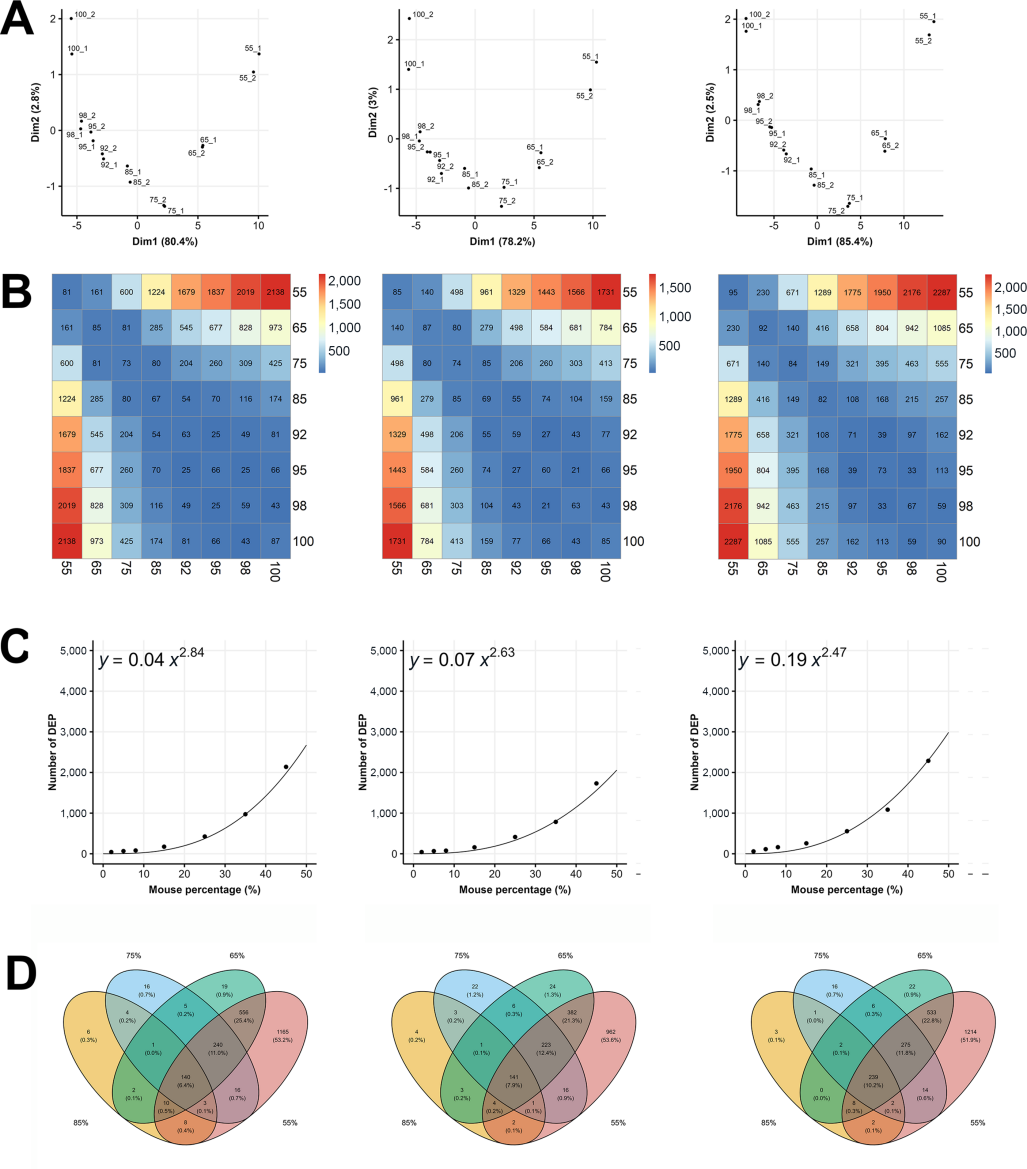
Human protein quantification in human and mouse cell line mixtures. Results from three methods (Full-peptide, Unique-peptide, and Human-only) are shown in the left, middle, and right panels in each graph. There are eight biological samples with a titration of human cell percentages (100, 98, 95, 92, 85, 75, 65, 55). Each biological sample has two technical replicates, denoted by numbers 1 and 2 after the underscore character. A protein's average expression in two technical replicates is used as its expression for the biological sample. **A,** The first two dimensions from PCA on 16 samples. **B,** The number of DEPs between two biological samples with a 1% FDR. **C,** The number of DEPs increases with mouse percentage, as described by a fitted power curve. **D,** Overlapping of DEPs for four biological samples with human cell percentages 55, 65, 75, and 85.

To evaluate proteomic profiling quality, we performed principal component analysis (PCA) on human proteins from the 16 samples and observed that technical replicates were always adjacent to each other, indicating that the variability of data acquisition was well controlled (Fig. 1A). In addition, the first principal component explains about 80% of the variance in the data, and along the axis there are samples with decreasing human cell percentage from left to right, demonstrating greater impact to the quantification of human proteins with increasingly more mouse proteins in the mixtures, which we will explore further below.

#### More Human Proteins were Falsely Identified as Differentially Expressed with Increasing Mouse Cell Percentages

**FIGURE 2:**
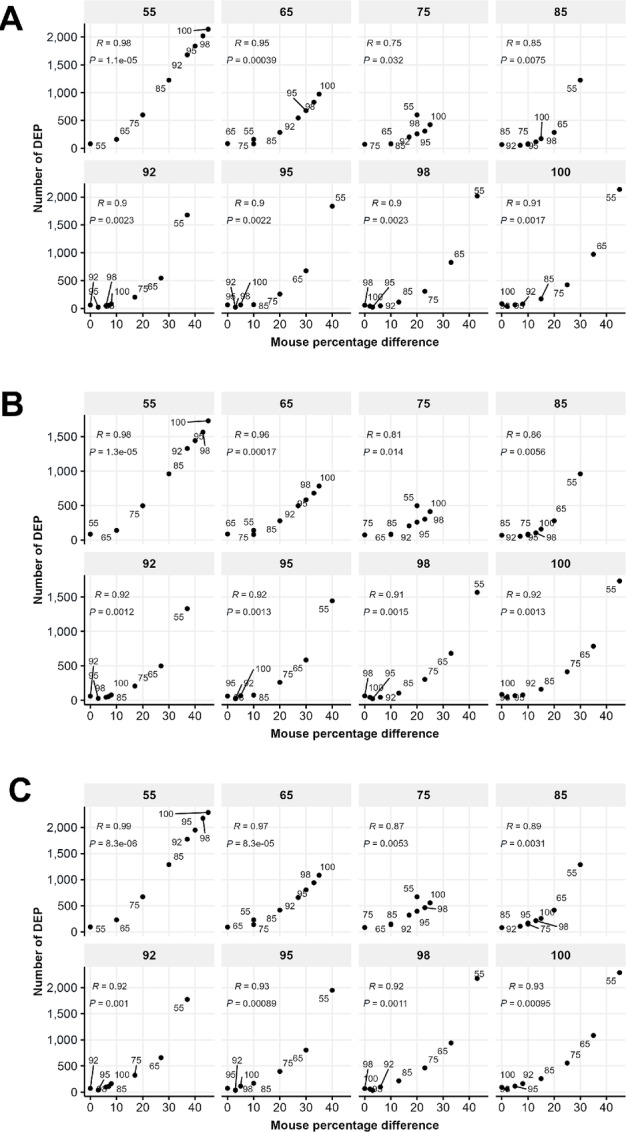
Number of DEPs for human proteins in human and mouse cell line mixtures. Results are shown for three methods: Full-peptide (**A**), Unique-peptide (**B**), and Human-only (**C**). There are eight biological samples with a titration of human cell percentages (100, 98, 95, 92, 85, 75, 65, 55). For every sample, the number of DEPs was calculated between it and each of the other seven samples with a 1% FDR. The positive association between the number of DEPs and mouse percentage difference is assessed by the Pearson correlation coefficient with an associated *P* value.

To assess the effect of mouse proteins on the quantification of human proteins in cell line mixtures, we identified seemingly DEPs between samples with different mouse cell percentages. We emphasize that these human DEPs were not truly differentially expressed between these samples, but merely artifacts due to the influence of mouse proteins. The thresholds for differential expression were set to exclude 0.5% of proteins at both ends of the expression ratios (TMT ratios) between all pairs of technical replicates, that is, the FDR was set to 1% and we expected to see approximately 70–80 or fewer proteins beyond the thresholds between two technical replicates. As shown in the diagonal entries in Fig. 1B, this is indeed the case. Using the aforementioned thresholds, we found that the number of DEPs was also around or below 70–80 between samples with 92%, 95%, 98%, and 100% human cells for the Full-peptide and Unique-peptide methods. Therefore, we conclude that human protein quantification is not significantly affected by the existence of mouse proteins for these two methods, as long as the mouse cells are below approximately 8%. This is not the case for the Human-only approach, a mere 5% of mouse cells already resulted in 113 DEPs. The number of human proteins falsely identified as differentially expressed between the pure human 293T cells and their mixtures with mouse MC38 cells increased rapidly with the mouse cell percentage, which could be approximated by some power-law functions (Fig. 1C). In addition, DEPs identified in samples with higher human cell percentages were almost all contained in samples with lower human cell percentages (Fig. 1D), an observation that demonstrates high data quality and invites further exploration in the next section. The impact of mouse cell percentage on the number of DEPs was also revealed when samples with other mouse cell percentages were chosen as reference samples (Fig. 2).

#### Human and Mouse Common Peptides Distort Human Protein Quantification

Protein quantification in proteomics is based on the assignment of peptides to one or more proteins containing the peptides. Many peptides are common to human and mouse homologous proteins. By comparing the Human-only and Mouse-only results, we found 36,310 human-unique peptides, 13,366 mouse-unique peptides, and 35,566 human-mouse common peptides. The high percentage of common peptides distorts human protein quantification, resulting in false DEPs, as evidenced by several investigations below.

**FIGURE 3:**
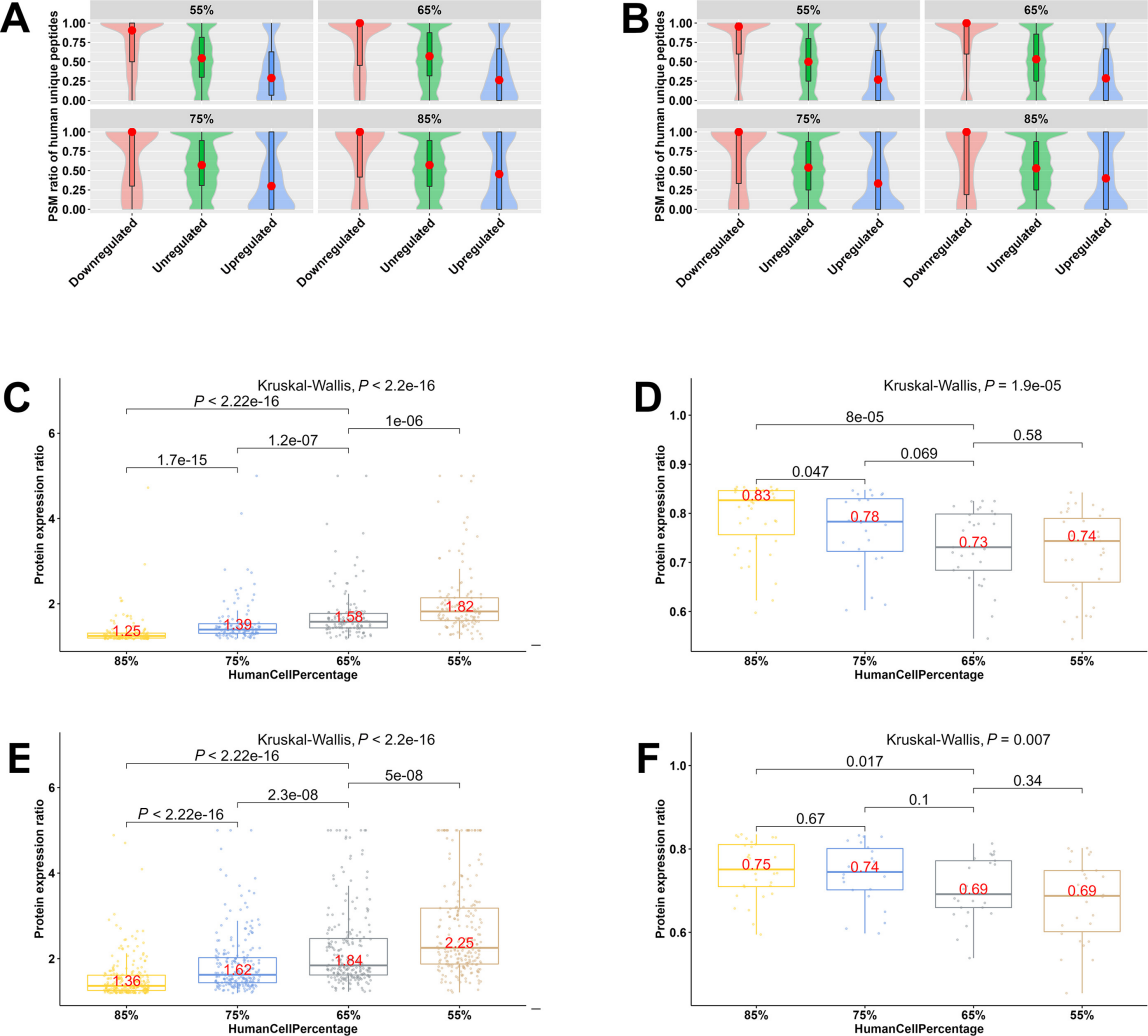
PSM distribution of human-unique peptides for proteins in human and mouse cell line mixtures. Violin plots depicting the distribution of PSM ratios of human-unique peptides for proteins from the Full-peptide (**A**) and Human-only methods (**B**). A red dot marks the median of a distribution. Proteins are classified as downregulated/upregulated/unregulated when compared with pure human cell samples for mixtures with 55%, 65%, 75%, and 85% human cells. PSM stands for peptide spectrum match. A higher PSM ratio indicates the protein is quantified by a higher percentage of PSM from human-unique peptides. PSM ratio is 1 or 0 if a protein is entirely quantified by human-unique peptides or human-mouse common peptides, respectively. Expression ratios for DEPs between human-mouse mixtures and pure human cell samples for Full-peptide (**C** and **D**) and Human-only results (**E** and **F**). Only DEPs common to four human cell percentages (85%, 75%, 65%, and 55%) are used. Numbers in red font are the distribution medians. *P* values for multiple-group comparisons are from the Kruskal–Wallis test. *P* values for pairwise comparisons are from Welch *t* test.

First, we computed the PSM ratio of the human-unique peptides for every protein. PSM stands for peptide spectrum match, each peptide has a corresponding PSM, and the number of PSM measures the abundance of the peptide. The PSM ratio of a protein is the ratio of PSM counts from human-unique peptides to the total PSM counts from both human-unique peptides and human-mouse common peptides. A higher PSM ratio indicates that the protein is quantified by more PSMs from human-unique peptides. The ratio is 1 if the protein is entirely quantified by human-unique peptides, and 0 if the protein is quantified only by human-mouse common peptides. When the pure human 293T cell samples were used as the reference, we observed that downregulated proteins generally have higher PSM ratios with medians close to or equal to 1, upregulated proteins have much lower PSM ratios, while unregulated proteins have in-between PSM ratios with medians around 0.5 (Fig. 3A and B). It is worth noting again that these human proteins appearing to be downregulated or upregulated were artifacts caused by mouse protein influence. Such phenomena can be explained by the interference of human-mouse common peptides in human protein quantification. Specifically, in human and mouse cell mixtures, many common peptides derived from mouse proteins are erroneously marked as from human proteins (the opposite, that is, marking human-origin common peptides as mouse-origin, exists to a far lesser extent due to the lower, sometimes far lower, percentage of mouse proteins and the corresponding algorithmic treatment), so the relative share of human-origin peptides decreases, and more so with increasing mouse protein percentages. Therefore, if a protein is primarily quantified using human-unique peptides (those with high PSM ratios), it appears to be downregulated. The PSM ratio increases as the percentage of mouse cells increases. On the contrary, if a protein is mainly quantified by human-mouse common peptides (ones with low PSM ratios), it appears to be upregulated because it takes up mouse-origin peptides as its own.

To more precisely evaluate how human-mouse common peptides affect protein expression measurement, we examined the change in DEP expression ratios between human and mouse mixtures and pure human cell samples to human cell percentage in the Full-peptide and Human-only results. We used only DEPs common to four human cell percentages (85%, 75%, 65%, and 55%) because there were sufficient numbers of DEPs while minimizing the effect of false-positive DEPs. In both datasets, the expression ratios for upregulated proteins steadily increased with the percentage of mouse cells but showed a descending trend for downregulated proteins (Fig. 3C–F). The explanation is that as the percentage of mouse cells increases, more mouse-origin common peptides are mistakenly marked as of human-origin, and the upregulated proteins are primarily quantified by common peptides; therefore, their relative expression among all proteins increases, so does the expression ratio; while the downregulated proteins are largely quantified by human-unique peptides and their relative expression decreases. Note that the analysis depicted in Fig. 3 did not consider the Unique-peptide method because no human-mouse common peptides were used.

**FIGURE 4:**
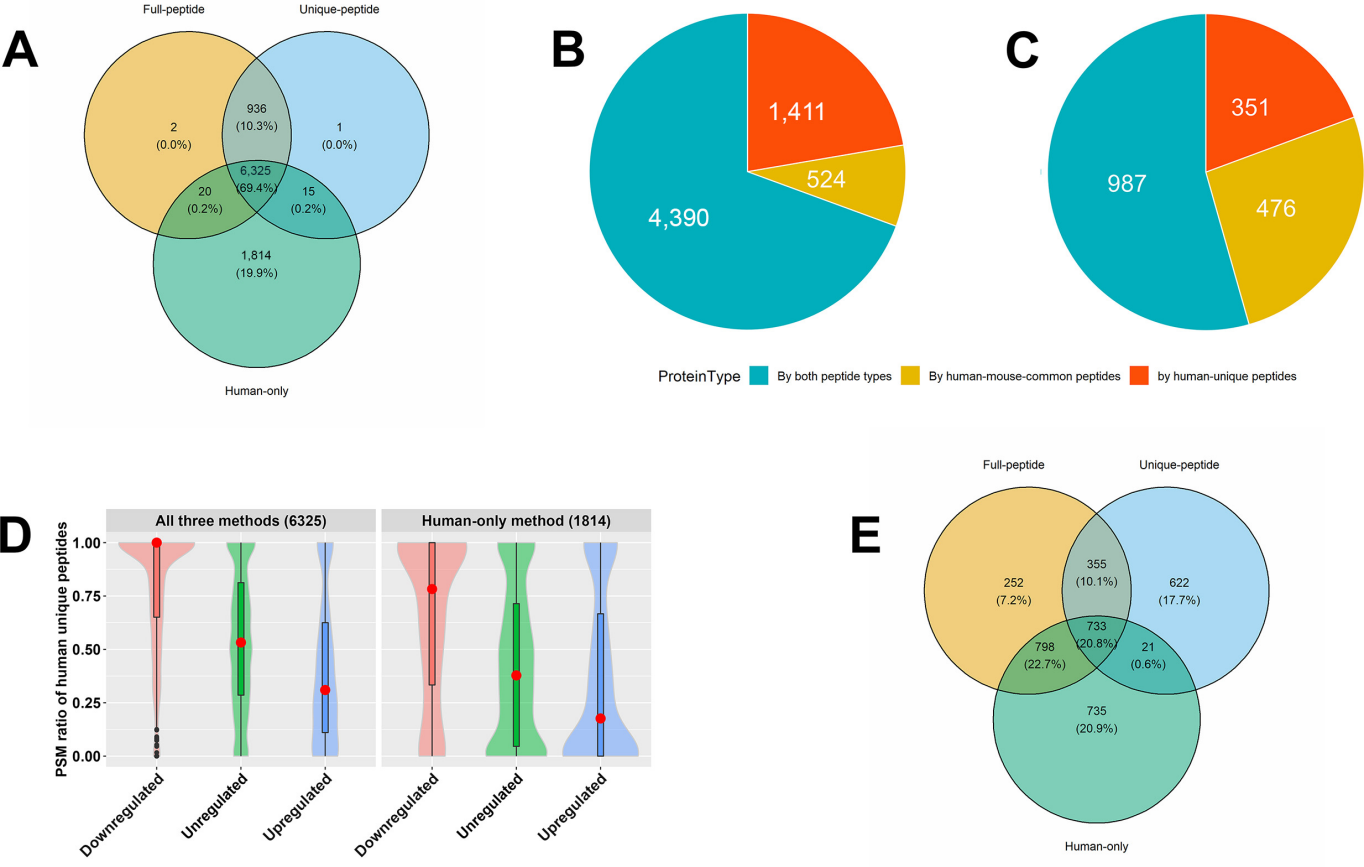
Comparison of proteins common to three methods and exclusive to the Human-only method in human and mouse cell line mixtures. **A,** Overlapping of quantified human proteins between the three methods. A higher proportion of human proteins are quantified solely by human-mouse common peptides in the 1,814 proteins (**B**) exclusive to the Human-only method than in the 6,325 proteins (**C**) common to all three methods. Specifically, 476 of the 1,814 (26.2%) proteins were solely quantified by human-mouse common peptides, while only 524 of the 6,325 (8.3%) proteins were so. **D,** The PSM ratios of human-unique peptides are comparatively higher in the 1,814 protein set for downregulated/upregulated/unregulated proteins. A red dot marks the median of a distribution. **E,** Overlapping of DEPs between the three methods when the human cell percentage is 55%.

The three quantification methods produced different sets of proteins, which essentially overlapped between the Full-peptide and Unique-peptide methods. The three methods shared 6,325 proteins, and the Human-only method owned 1,814 proteins exclusively (Fig. 4A). Because this method treats all peptides as only from human proteins, these exclusive proteins were more likely to be generated from or impacted by human-mouse common peptides, some of which were from mouse proteins. This postulation was confirmed by comparing the proportions of three protein classes between the 1,814 and 6,325 proteins (Fig. 4B and C). Specifically, among the 1,814 proteins, 476 (26.2%) proteins were solely quantified by human-mouse common peptides; in contrast, only 524 (8.3%) of the 6,325 proteins were so. Correspondingly, the PSM ratios of human-unique peptides were systematically lower for downregulated, upregulated, and unregulated proteins among the 1,814 proteins (Fig. 4D).

Finally, we examined the overlap of DEPs among the three methods when the percentage of human cells was 55% (Fig. 4E). We observed that each method produced unique proteins such that they shared only 733 proteins or 20.8% of all DEPs. The proportion of overlapping proteins between any two methods also varied (23.1%, 39.4%, and 52.9%, respectively). Hence, the use of more than one method will not satisfactorily exclude false DEPs. In conclusion, the large number of human-mouse common peptides makes it impossible to accurately quantify many human and mouse proteins, and there is no computational remedy once proteomics data for human and mouse cell mixtures are acquired.

### Human Protein Quantification in PDX Tumors

**FIGURE 5:**
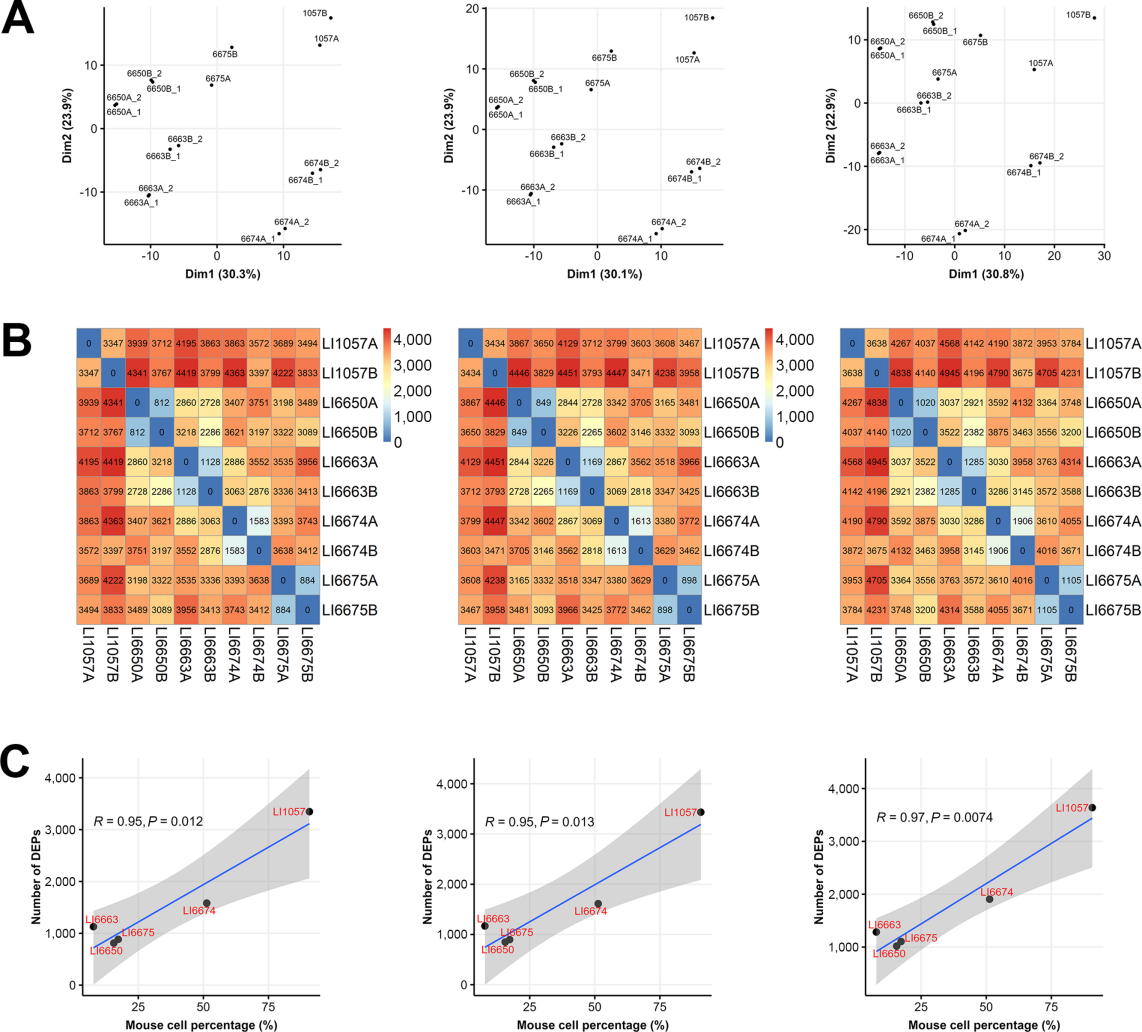
Human protein quantification in PDX tumors. Results from three methods (Full-peptide, Unique-peptide, and Human-only) are shown in the left, middle, and right panels in each graph. There are five liver PDX models each with an original tumor sample (ending with “**A**”) and a demoused tumor sample (ending with “**B**”), some of the samples have two technical replicates, denoted by numbers 1 and 2 after an underscore character. A protein's average expression in two technical replicates is used as its expression for the tumor. **A,** The first two dimensions from PCA on PDX tumor samples (**B**) The number of DEPs between samples with a 1% FDR. **C,** The number of DEPs between the original and demoused tumor samples of a PDX is positively correlated with mouse cell percentage in the original tumor sample. Pearson correlation coefficients and corresponding *P* values are shown.

Similar to the cell line data, we also quantified protein expression for PDX tumors by the aforementioned four methods, and we again focused on human protein expression by including proteins with all confidence levels (Table 3). PDX tumors showed more divergent human protein expression patterns than cell line samples such that the first two principal components accounted for only approximately 50% of the variance in the data. Technical replicates were tightly clustered, and demoused tumors were close to the original tumors from the same PDX models (Fig. 5A).

**TABLE 3:** Number of quantified proteins by four methods in PDX tumors

	Full-peptide	Unique-peptide	Human-only	Mouse-only
Quantified human proteins	7,354 (6,809, 429, 116)^a^	7,334 (6,790, 428, 116)	8,323 (7,673, 554, 96)	0
Quantified mouse proteins	2,687 (2,415, 216, 56)	2,669 (2,397, 216, 56)	0	6,815 (6,105, 645, 65)

^a^Number of proteins with abundance quantification in all 16 samples; the three numbers in parentheses are the number of proteins with high, medium, and low quantification confidence (FDR < 0.01, 0.05, and 0.1, respectively).

We calculated thresholds for calling differential protein expression with a 1% FDR using six pairs of technical replicates (Table 1). Subsequently, we identified DEPs between samples that were not technical replicates—the off-diagonal entries in the matrix (Fig. 5B). There were generally 3,000–4,000 DEPs between different PDX models for either original or demoused tumor samples, reflecting the complex and heterogeneous proteomes between PDX models, especially with different levels of mouse stromal infiltration. There were more DEPs between LI1057 samples (both LI1057A and LI1057B) and other PDX samples than between other PDX samples themselves. LI1057A had an exceptionally high mouse cell percentage (90.7%), and there was significant amount of residual mouse stroma in LI1057B (4.9%) even after applying the mouse depletion kit. This is likely the cause of the excessive DEPs for LI1057.

We examined the impact of mouse stroma on human protein quantification from two perspectives. In the intra-PDX study, we identified DEPs between the original and demoused tumor samples of a PDX, and observed a strong positive correlation between the number of DEPs and the percentage of mouse cells in the original tumor sample (Fig. 5C). The top 1,000 DEPs were investigated by the gene set enrichment analysis (Supplementary Table S2). In all three methods, we observed the enrichment of cellular components for extracellular matrix, focal adhesion and cytoskeleton organization, and identified strong lysosome formation with the participation of relevant secretory and endocytic pathways, as well as an active translation for protein production via the ribosome. In addition, the Human-only method prioritized the epithelial–mesenchymal transition pathway, whereas the Unique-peptide method presented genes regulated by MYC and upregulated via the activation of the MTORC1 complex. Clearly, the enrichment of these pathways in the original tumors came from the mouse stromal proteins that erroneously appeared to be human DEPs when compared against demoused tumors.

**FIGURE 6:**
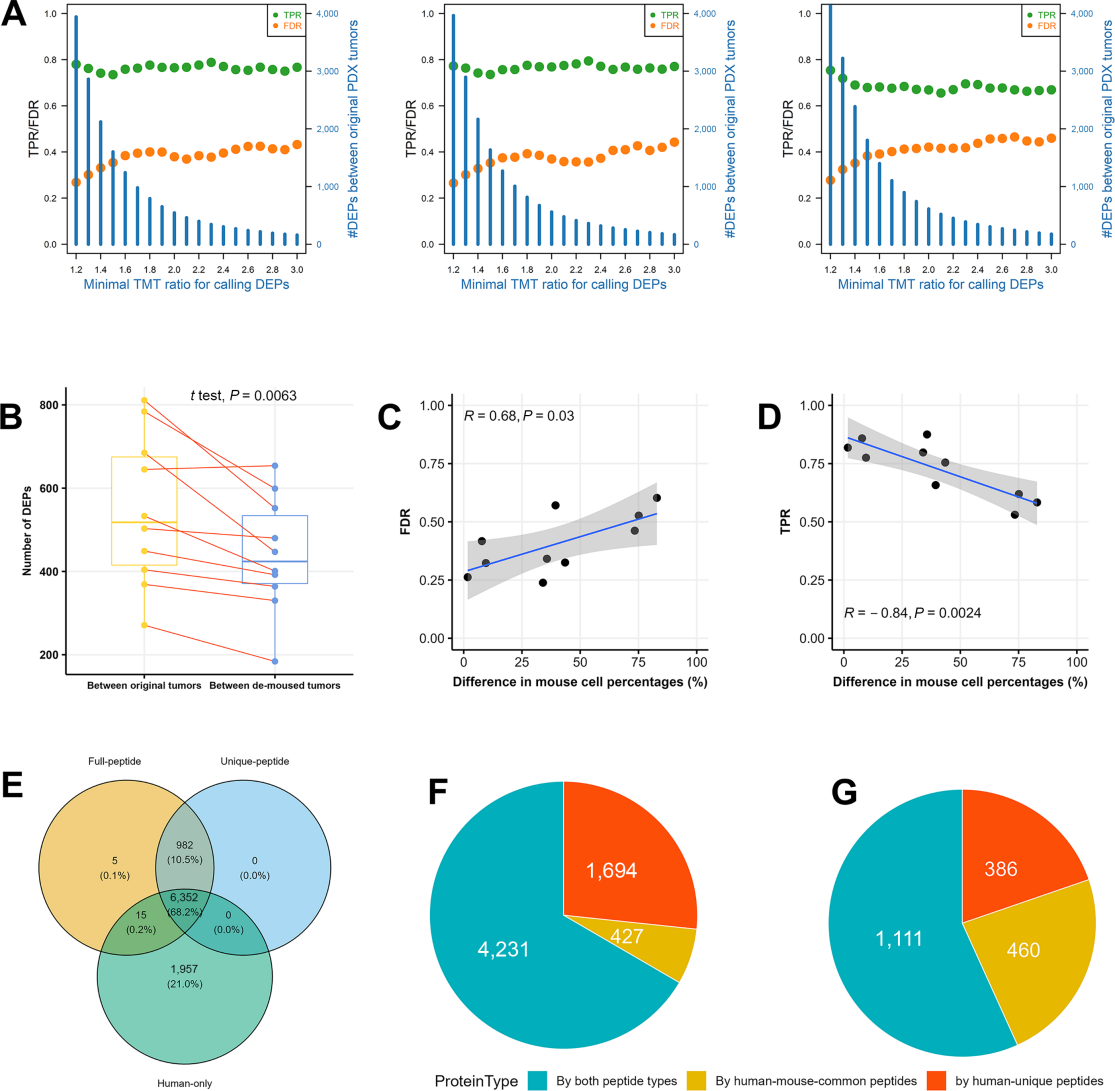
Human protein quantification between PDX models. **A,** DEPs from pairwise comparison between PDX models at minimal expression ratios (i.e., minimal TMT ratios) ranging from 1.2 to 3.0 with methods detailed in main text. FDR is false discovery rate, TPR is true positive rate that is also called recall and sensitivity. At each minimal TMT ratio, all 10 pairwise comparisons were performed between the five PDX models, and the median values of FDR, TRP, and number of DEPs were shown. Results from three methods (Full-peptide, Unique-peptide, and Human-only) are shown in the left, middle, and right panels. **B,** Number of DEPs obtained by the Full-peptide method when minimal TMT ratio was set to 2.0. A red line connects a pair of PDX models for which DEPs were identified between two original tumors and between two demoused tumors. *P* value was from paired *t* test. **C** and **D**, A positive/negative correlation exists between FDR/TPR and the difference in mouse cell percentages in original tumors of two PDX models. **E,** Overlapping of quantified human protein between three methods. **F** and **G**, A higher proportion of proteins were quantified solely by human-mouse common peptides in the 1,957 proteins exclusive to the Human-only method than in the 6,352 proteins common to all three methods.

In the inter-PDX study, we set to obtain DEPs between different PDX models. To this end, we performed the following analysis for every pair of PDX models: (i) We first compared the two demoused tumor samples to obtain DEPs with expression ratio larger than a threshold (minimal TMT ratio). These DEPs were considered true positives (TP). (ii) We then performed the same analysis for the two original tumor samples using the identical threshold. If a DEP identified here was not a true positive, it was designated a false positive (FP). On the other hand, if a true positive was not identified here, it was seen as a false negative (FN). Subsequently, we computed a FDR [FDR = FP/(FP+TP)] and a true positive rate [TPR = TP/(TP+FN), also called recall and sensitivity] (3). The two steps were repeated for a series of thresholds ranging from 1.2 to 3.0, and the results were summarized in Fig. 6A.

We found that when the original tumors were used for proteomic profiling, there were always 20%–30% (i.e., 1-TPR) of human proteins with real differential expression between two PDX models evading detection. Meanwhile, about 30%–40% (FDR) of identified DEPs were actually false positives, their expression levels were not so much different (i.e., below minimal TMT ratio) in the demoused tumors. At low thresholds (e.g., minimal TMT ratio = 1.2–1.5), we have favorably smaller FDR and larger TPR values, however, there were too many DEPs (about 2,000–4,000), so the number of false-positive DEPs were equally large and prohibitive for systematic experimental validation. In practice, we normally set more stringent thresholds and only select the most DEPs for further investigations, yet even if the Minimal TMT ratio was increased to 3.0 that reported only about 100 DEPs, about 40 of them were still not truly differentially expressed in human tumor cells between two PDX models. At the same minimal TMT ratio, there were generally more DEPs from comparison between original tumors than between demoused tumors (Fig. 6B). In addition, FDR increases while TPR decreases with the difference in mouse cell percentages between two original tumors (Fig. 6C and D).

In both the intra- and inter-PDX studies, the prevailing existence of false DEPs resulted from human-mouse common peptides so that mouse proteins distorted human protein quantification, same as in human-mouse cell line mixtures. In the 16 PDX tumor samples, we jointly identified 39,971 human-unique peptides, 10,958 mouse-unique peptides, and 33,431 human-mouse common peptides. We demonstrate this using a single analysis. There were 6,352 human proteins quantified by all three methods, and 1,957 proteins were exclusively quantified by the Human-only method (Fig. 6E). These two sets of proteins possessed very different proportions of proteins quantified solely by human-mouse common peptides, 23.5% vs. 6.7% (460 vs. 427 proteins in Fig. 6F and G), and these two ratios were close to those observed in the cell line samples (26.2% vs. 8.3%).

## Discussion

Xenografts are an invaluable model system with a dichotomous composition of human cancer cells and mouse noncancerous cells (11). This unique advantage can be explored not only to understand fundamentals of cancer biology, such as stromal and tumor-specific gene and protein expression, signal transduction between tumor and stroma, and the impact of implantation and metastasis on transcriptomics and proteomics, but also for oncology drug development encompassing drug mechanism investigation and biomarker discovery (9, 29). All these rely on accurate omics data from xenograft models. This report demonstrates that a better way (separate-then-run) to perform proteomic profiling of xenografts is to separate human and mouse cells before protein extraction and experimental data acquisition via MS.

The difficulty of separating and allocating human and mouse peptides seems underappreciated and the run-then-separate approach is still the mainstream strategy for xenograft proteomics. Several computational methods exist to process xenograft proteomics data, none of which, as we showed here, works satisfactorily because of the copious number of human-mouse common peptides. Proteins are digested by trypsin to generate short peptides with of approximately 7–40 amino acid residues and then characterized by MS. In both human-mouse cell line mixtures and PDX tumors, approximately half of the characterized peptides are common between human and mouse. In addition, there are common peptides among human proteins or mouse proteins themselves. In the Full-peptide method, common peptides are included but not properly assigned because they are often allocated to only one protein, the so-called master protein in a protein group, which has the highest expression in the group based on its unique peptide abundance. In addition, proteins are not equally affected by human-mouse common peptides, as gauged by the PSM ratio. In the Unique-peptide method, common peptides are excluded to avoid incorrect assignment; however, it introduces a new bias because only half of the peptide abundance information can be used to quantify protein expression while proteins are also unequally affected by such omission. In the Human-only method, the problem of common peptide assignment exists and is further complicated by treating mouse peptides as human peptides. As a result, we obtained quantification information for approximately 1,000 more doubtingly human proteins, yet there are about 2,000 proteins exclusive to this method and 1,000 unique to the other two methods, which is caused by different protein groupings in the Proteome Discoverer software. Any variant or combination of the three methods encounters the common-peptide problem and is not likely to achieve good performance.

For the quantification of human protein expression, PDX tumors appear more sensitive to mouse cell infiltration than human cancer cell lines, reflecting their more complex compositions. In PDX tumors, cancer cells are more heterogeneous than immortal cancer cell lines, and mouse cells include immune cells and blood vessel endothelial cells besides common stromal cells. The difference may also be contributed by different experimental protocols. For the cell line mixtures, we extracted proteins from 293T cells and MC38 cells individually and then mixed them by weigh to obtain human and mouse protein percentages. For PDX tumors, we directly extracted proteins from tumors of both human and mouse cells, and then determined the human and mouse cell percentages by measuring their relative DNA amount. It is known that cells are of different sizes (30), and there are variations in protein mass per unit volume (31).

We investigated false DEPs for PDX tumors by both intra- and inter-PDX studies. In the intra-PDX studies, there are already approximately 1,000 DEPs between the original and demoused tumor samples when there are 7.8% mouse cells in the liver PDX tumor LI6663. If we extrapolate to set the threshold of mouse cells at 5% without impeding human protein quantification, about 85% of PDX tumors surpass this threshold (11). Some cancers, such as pancreatic cancer and clear-cell renal carcinoma, are more prone to mouse infiltration because they have high stromal content. In the inter-PDX studies, we demonstrated the risk of both false positives and false negatives. Spurious results can arise from xenograft proteomics data that do not separate human and mouse cells before data acquisition.

In this benchmark study, we used TMT-labeling proteomics technology. It is more sensitive and accurate than commonly used bottom-up proteomics technologies, including both label-free data-dependent acquisition and data-independent acquisition approaches, for which the problems encountered in the TMT approach are likely to worsen. We would also like to emphasize that cell separation is also recommended for mixtures of two or more species with high protein homology.

## Supplementary Material

Table S1 Protein quantification results in cell line mixtures and PDX tumors.

Table S2 Gene set enrichment analysis results using the top 1000 DEPs between the original and de-moused tumors for each of five PDX models.
