# Supplementary material for: Mouse Stromal Cells Confound Proteomic Characterization and Quantification of Xenograft Models
Source: Cancer Res Commun. 2023 Feb 6;3(2):202–14. doi: 10.1158/2767-9764.CRC-22-0431 (PMC10035517; doi:10.1158/2767-9764.CRC-22-0431)
Supplement: Table S2 — Gene set enrichment analysis results using the top 1000 DEPs between the original and de-moused tumors for each of five PDX models. [file crc-22-0431-s02.pptx]

## Slide 1
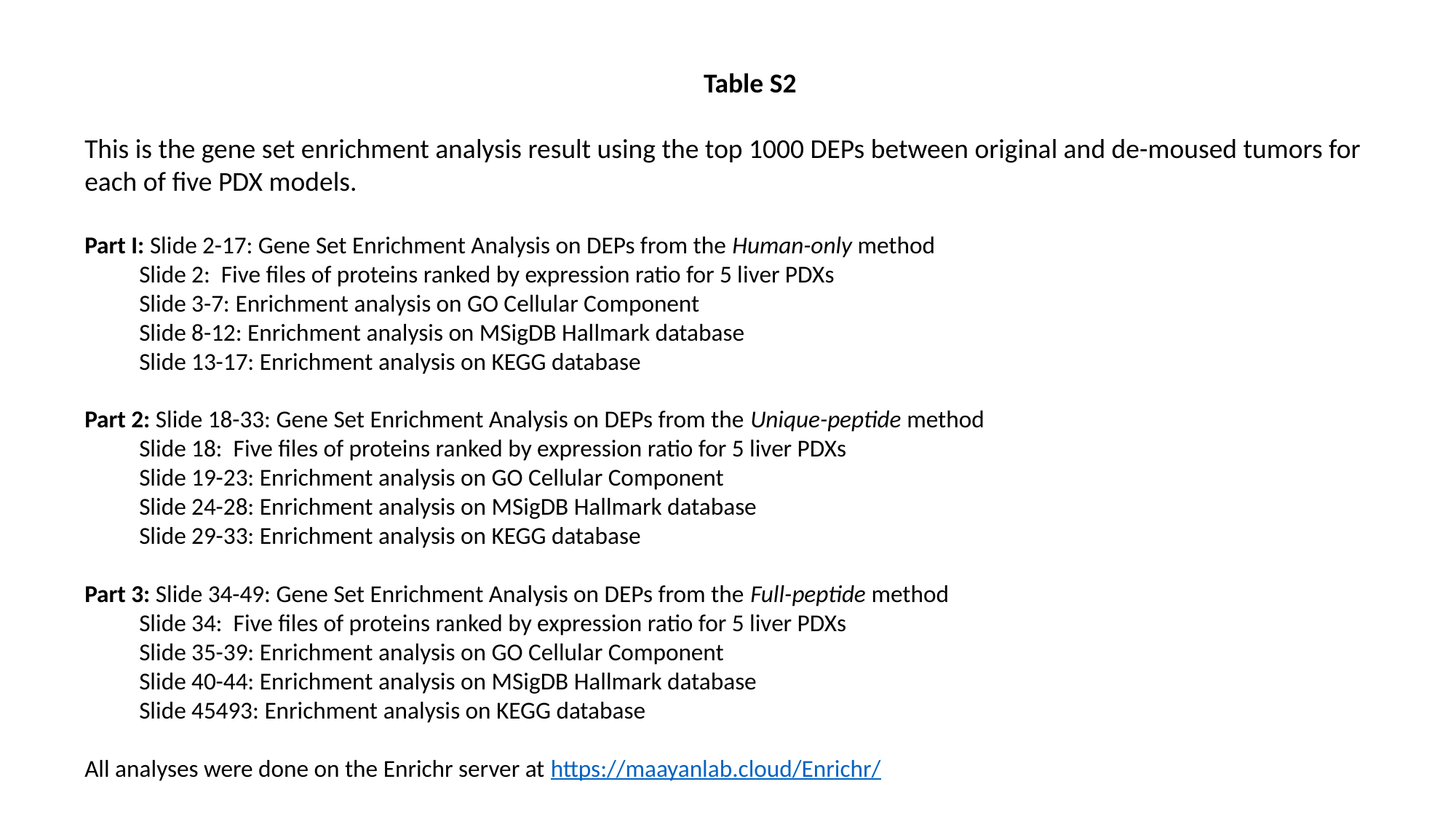

Table S2
This is the gene set enrichment analysis result using the top 1000 DEPs between original and de-moused tumors for each of five PDX models.
Part I: Slide 2-17: Gene Set Enrichment Analysis on DEPs from the Human-only method
Slide 2: Five files of proteins ranked by expression ratio for 5 liver PDXs
Slide 3-7: Enrichment analysis on GO Cellular Component
Slide 8-12: Enrichment analysis on MSigDB Hallmark database
Slide 13-17: Enrichment analysis on KEGG database
Part 2: Slide 18-33: Gene Set Enrichment Analysis on DEPs from the Unique-peptide method
Slide 18: Five files of proteins ranked by expression ratio for 5 liver PDXs
Slide 19-23: Enrichment analysis on GO Cellular Component
Slide 24-28: Enrichment analysis on MSigDB Hallmark database
Slide 29-33: Enrichment analysis on KEGG database
Part 3: Slide 34-49: Gene Set Enrichment Analysis on DEPs from the Full-peptide method
Slide 34: Five files of proteins ranked by expression ratio for 5 liver PDXs
Slide 35-39: Enrichment analysis on GO Cellular Component
Slide 40-44: Enrichment analysis on MSigDB Hallmark database
Slide 45493: Enrichment analysis on KEGG database
All analyses were done on the Enrichr server at https://maayanlab.cloud/Enrichr/

## Slide 2
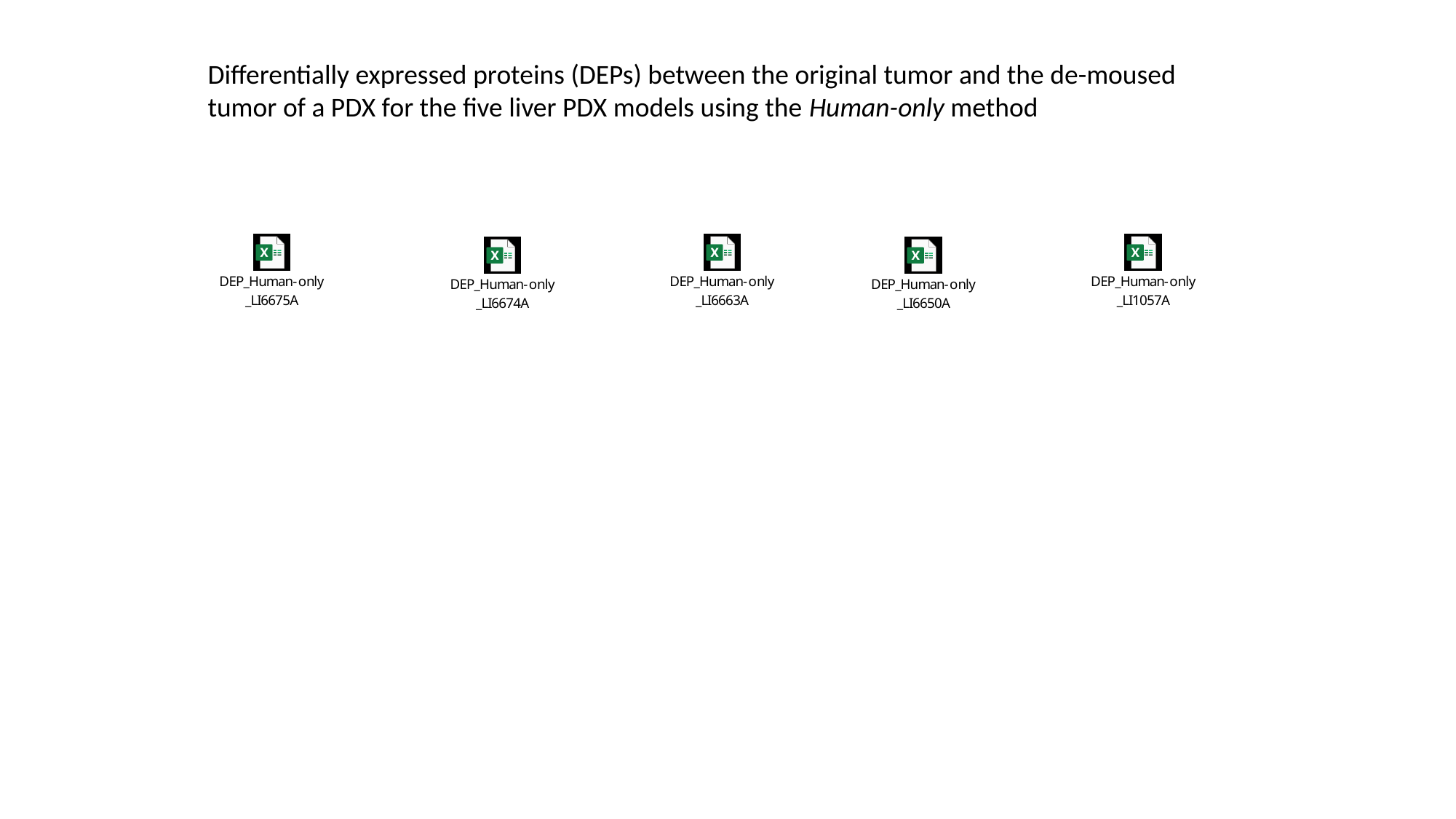

Differentially expressed proteins (DEPs) between the original tumor and the de-moused tumor of a PDX for the five liver PDX models using the Human-only method

## Slide 3
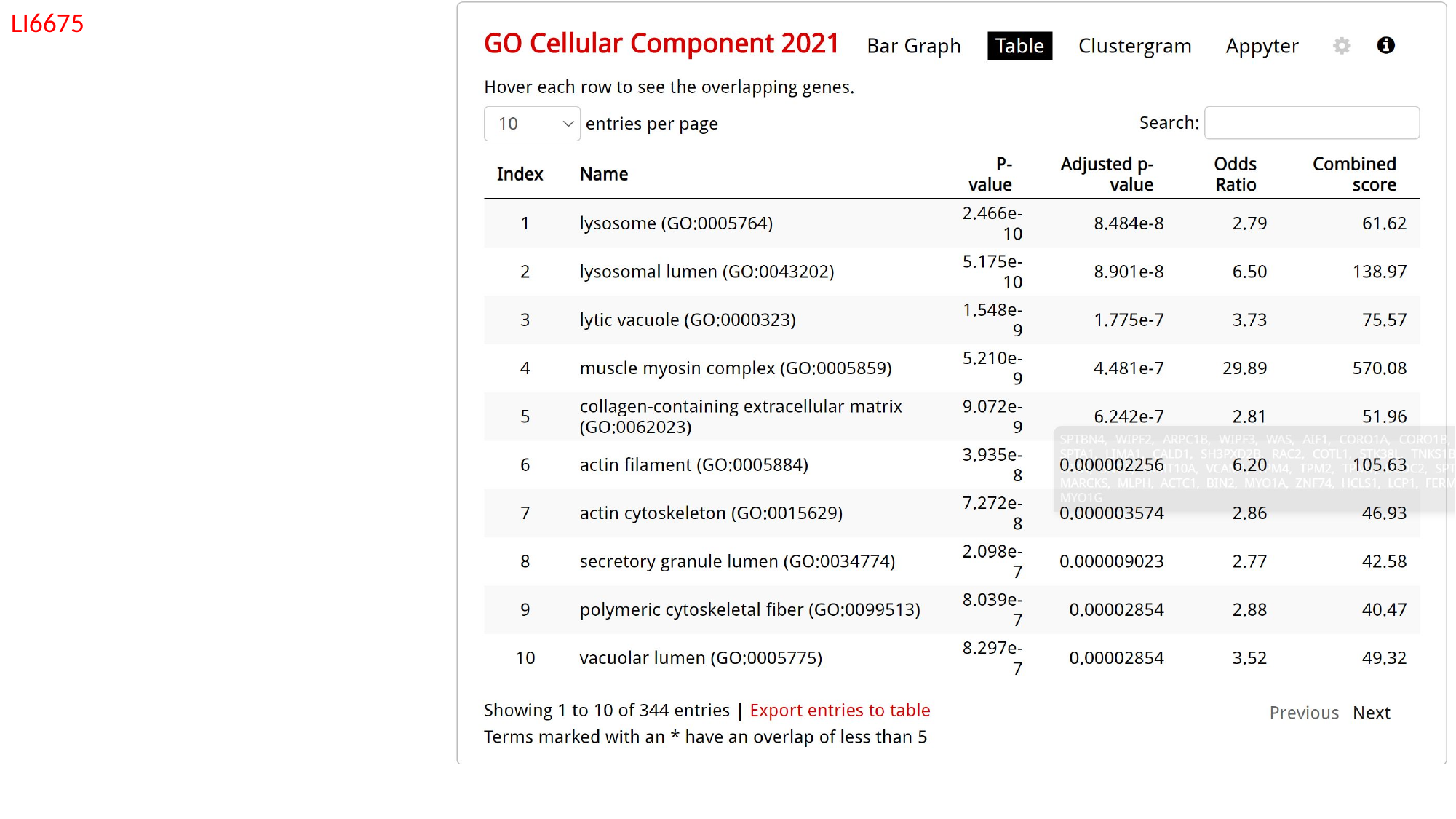

LI6675

## Slide 4
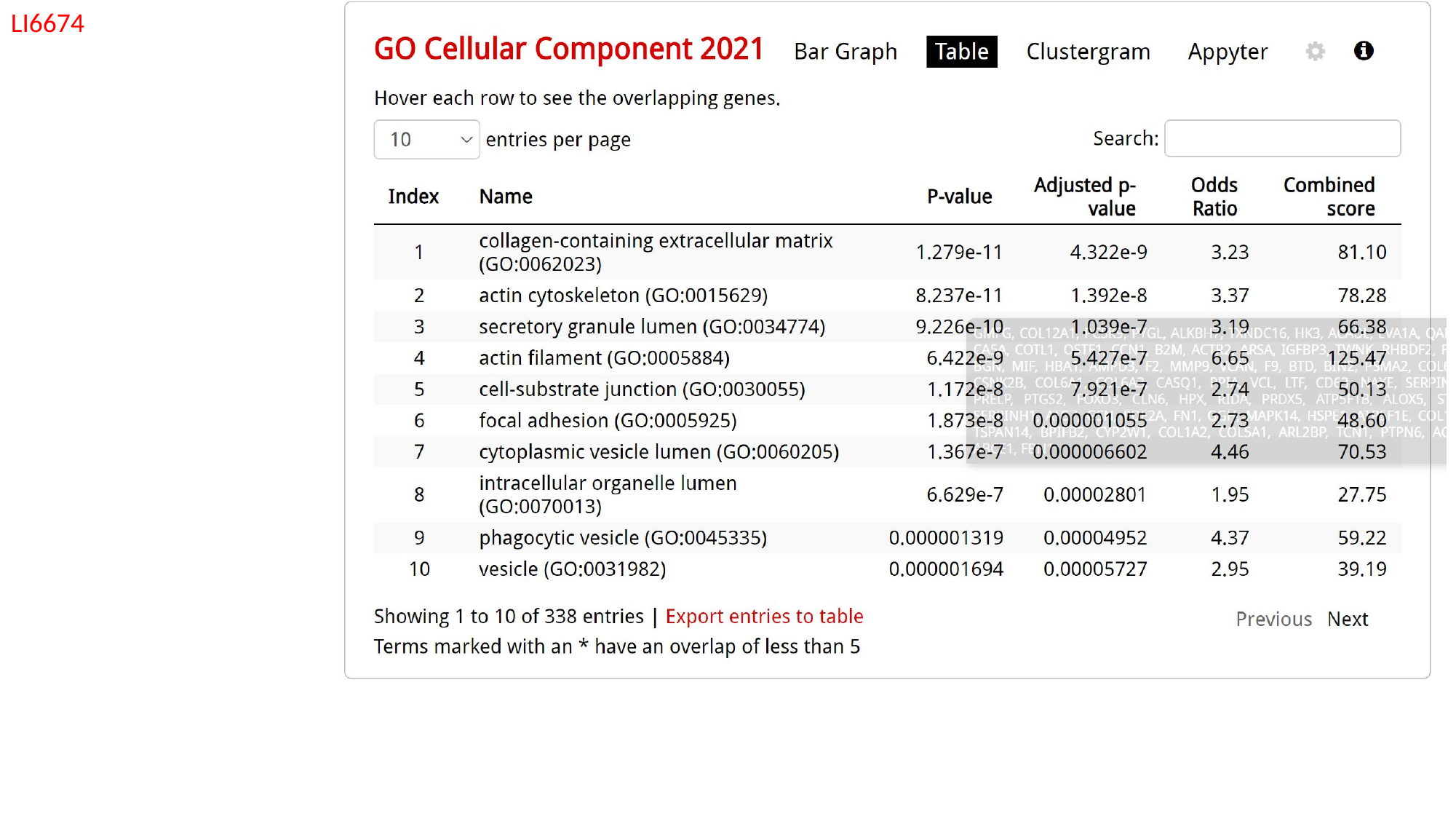

LI6674

## Slide 5
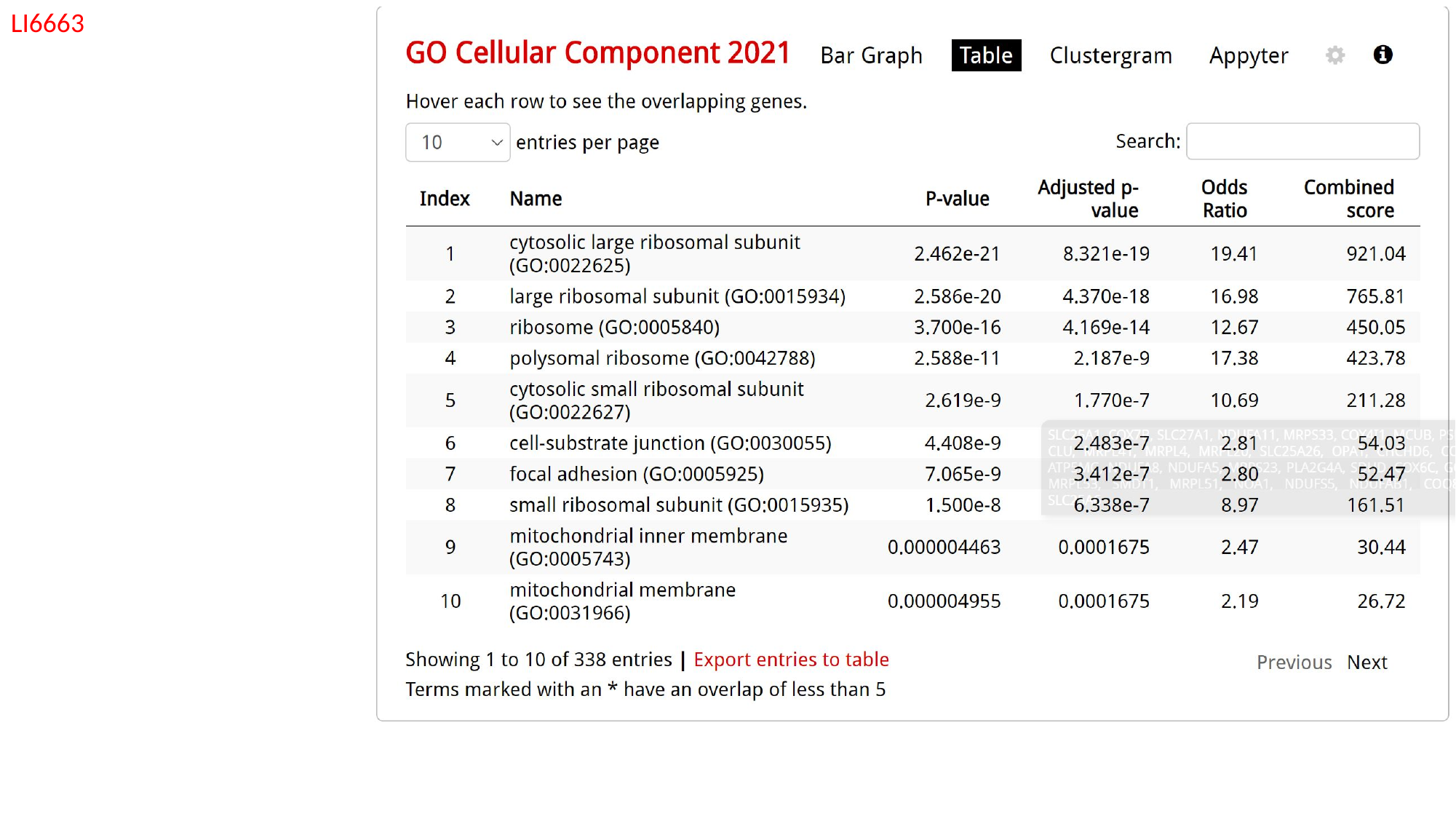

LI6663

## Slide 6
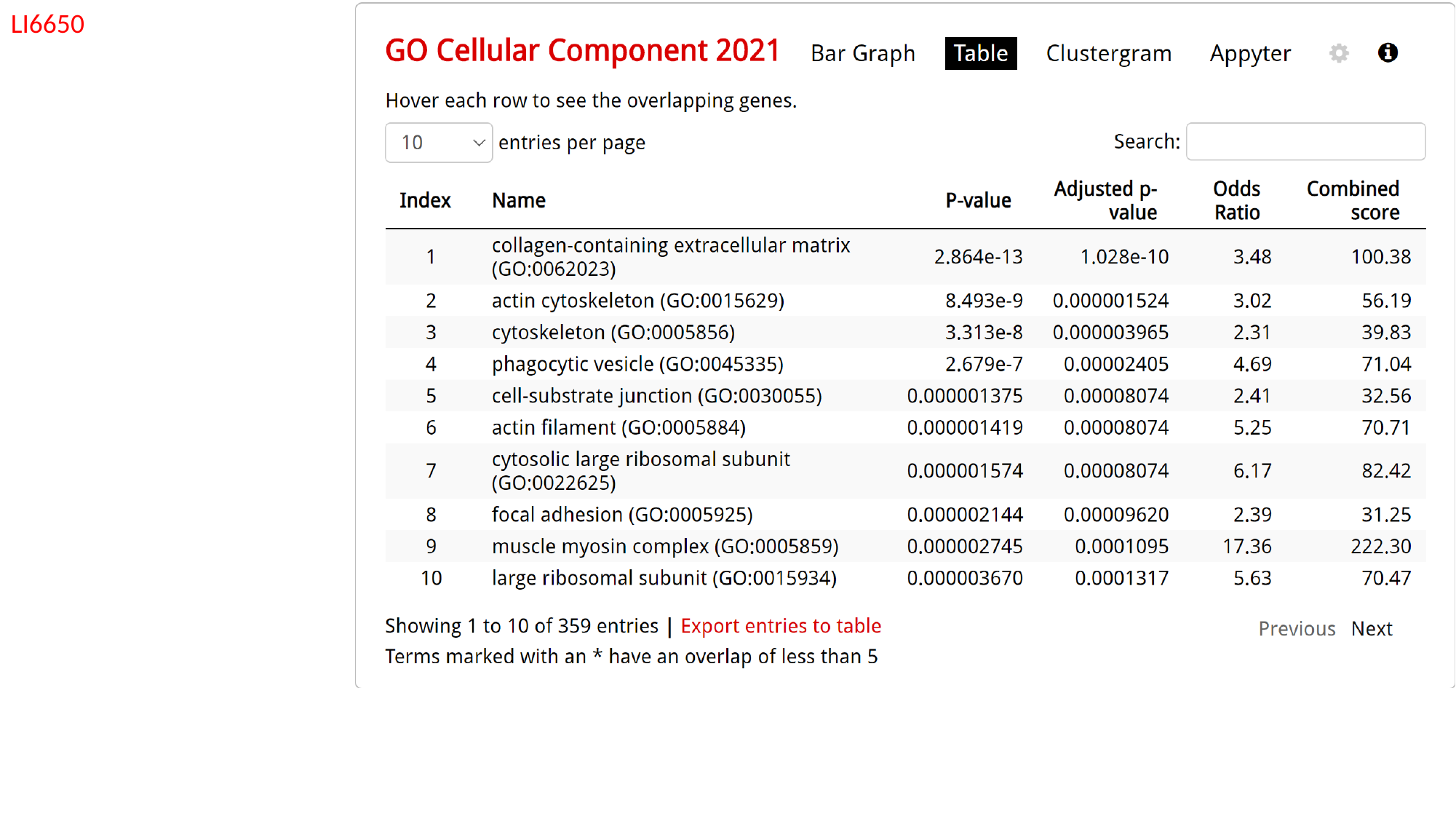

LI6650

## Slide 7
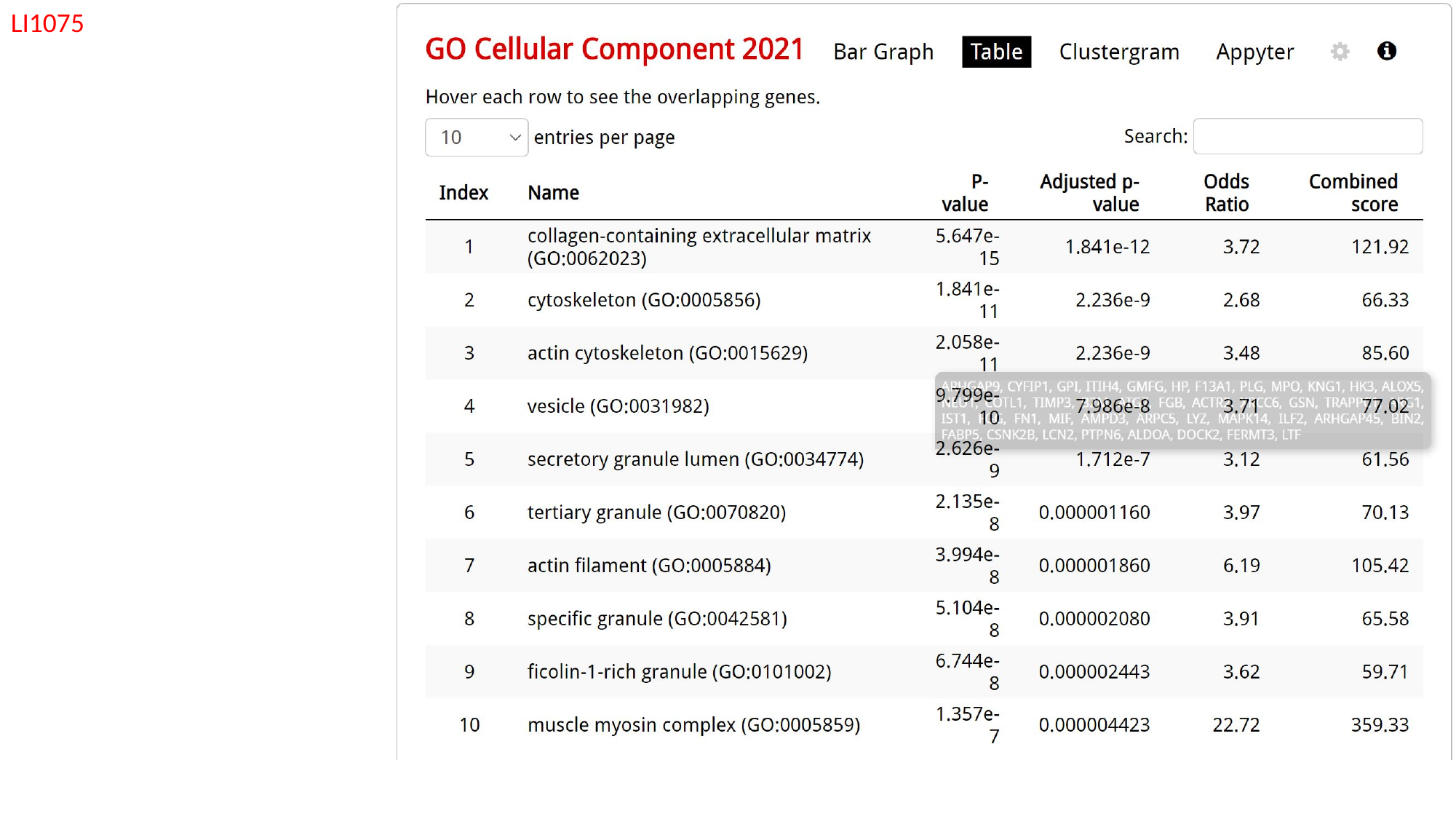

LI1075

## Slide 8
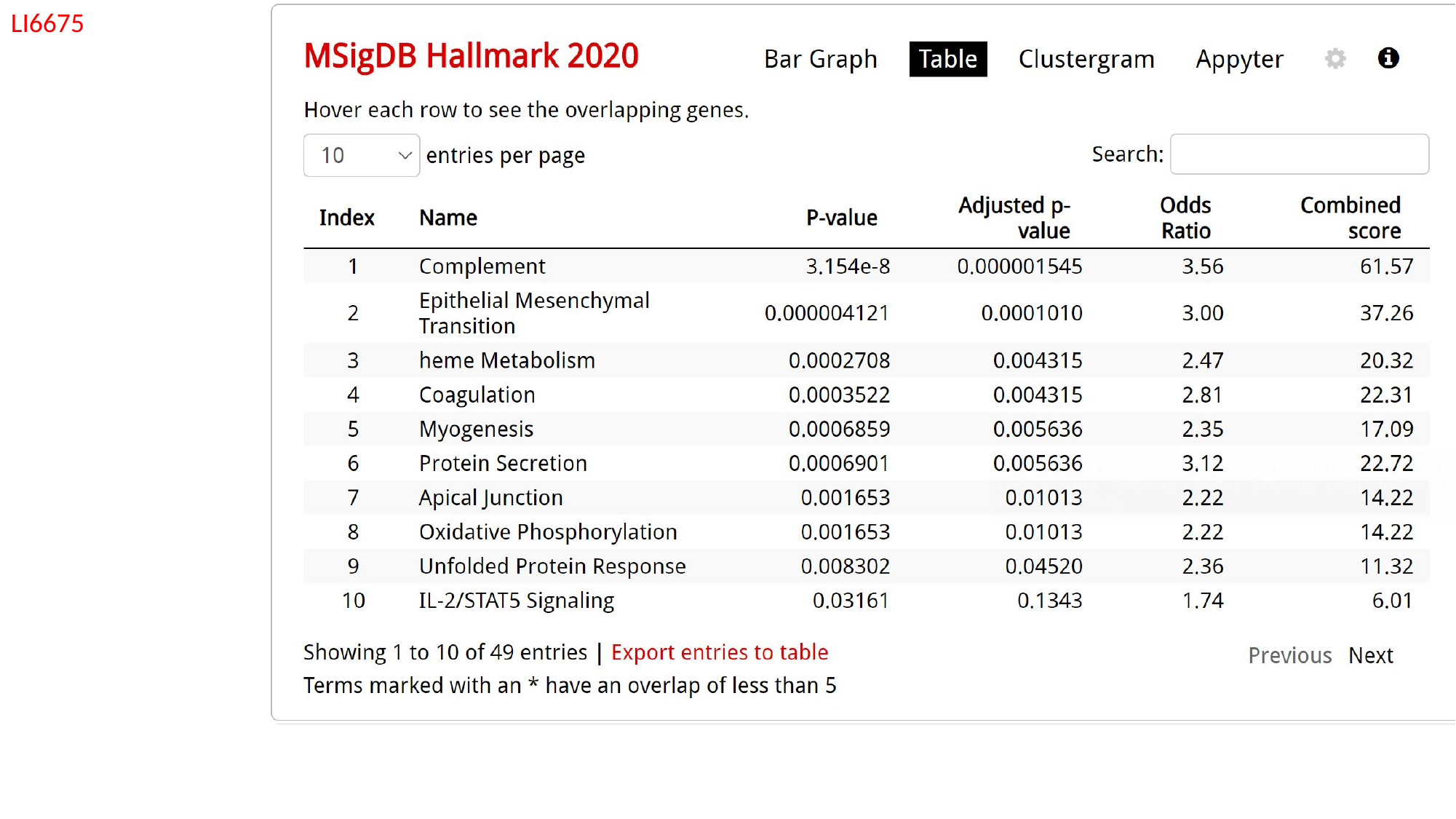

LI6675

## Slide 9
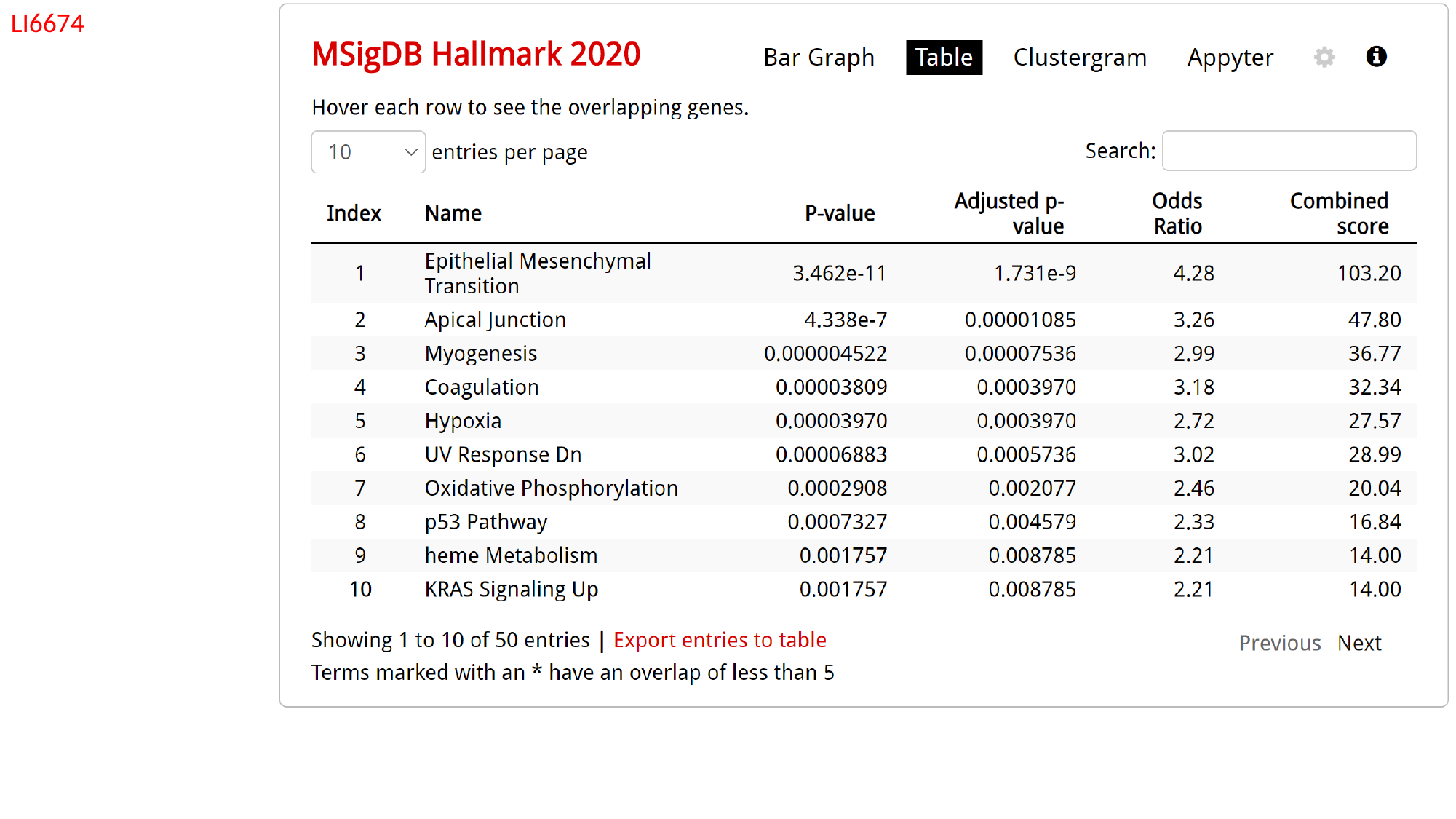

LI6674

## Slide 10
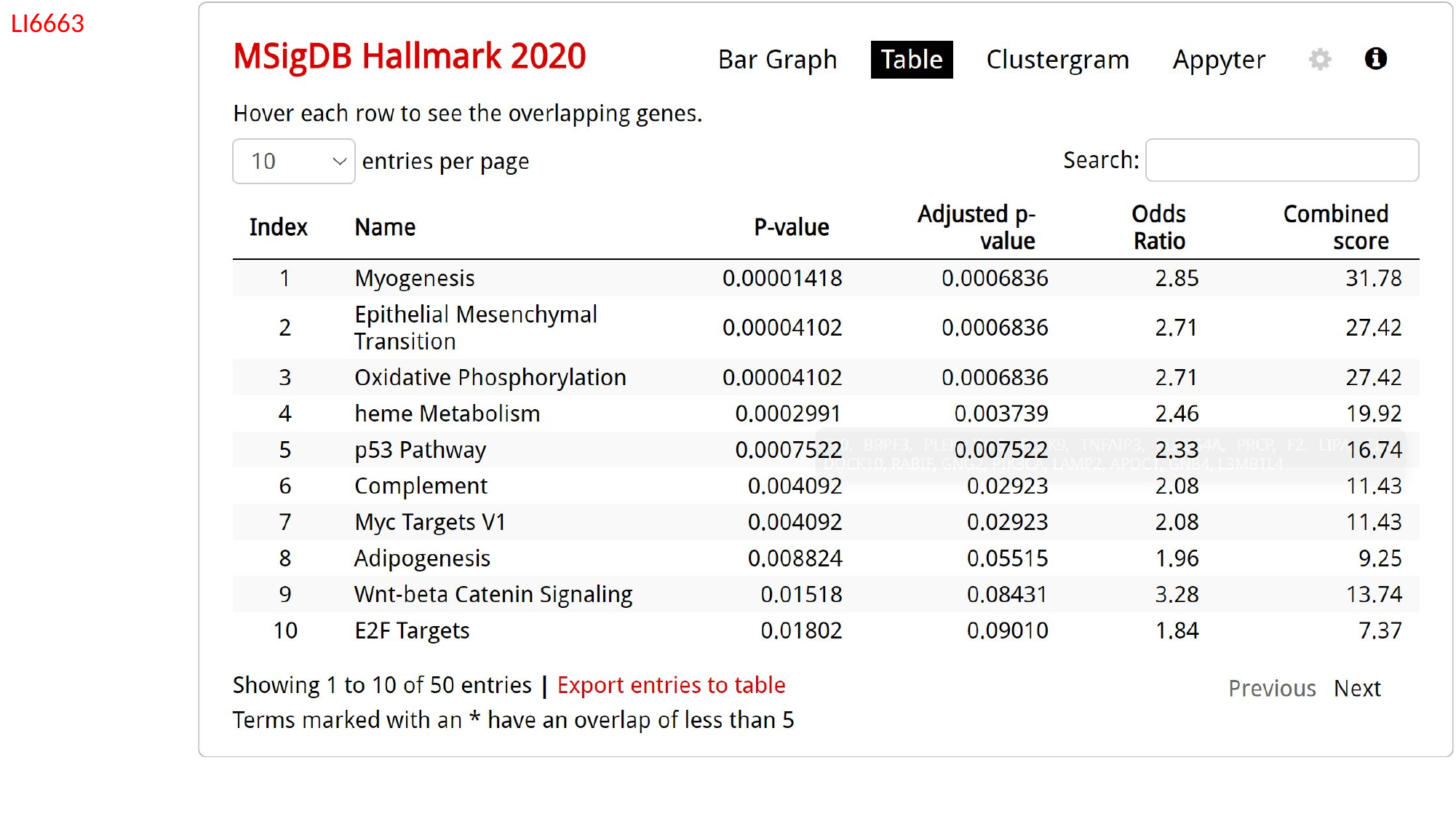

LI6663

## Slide 11
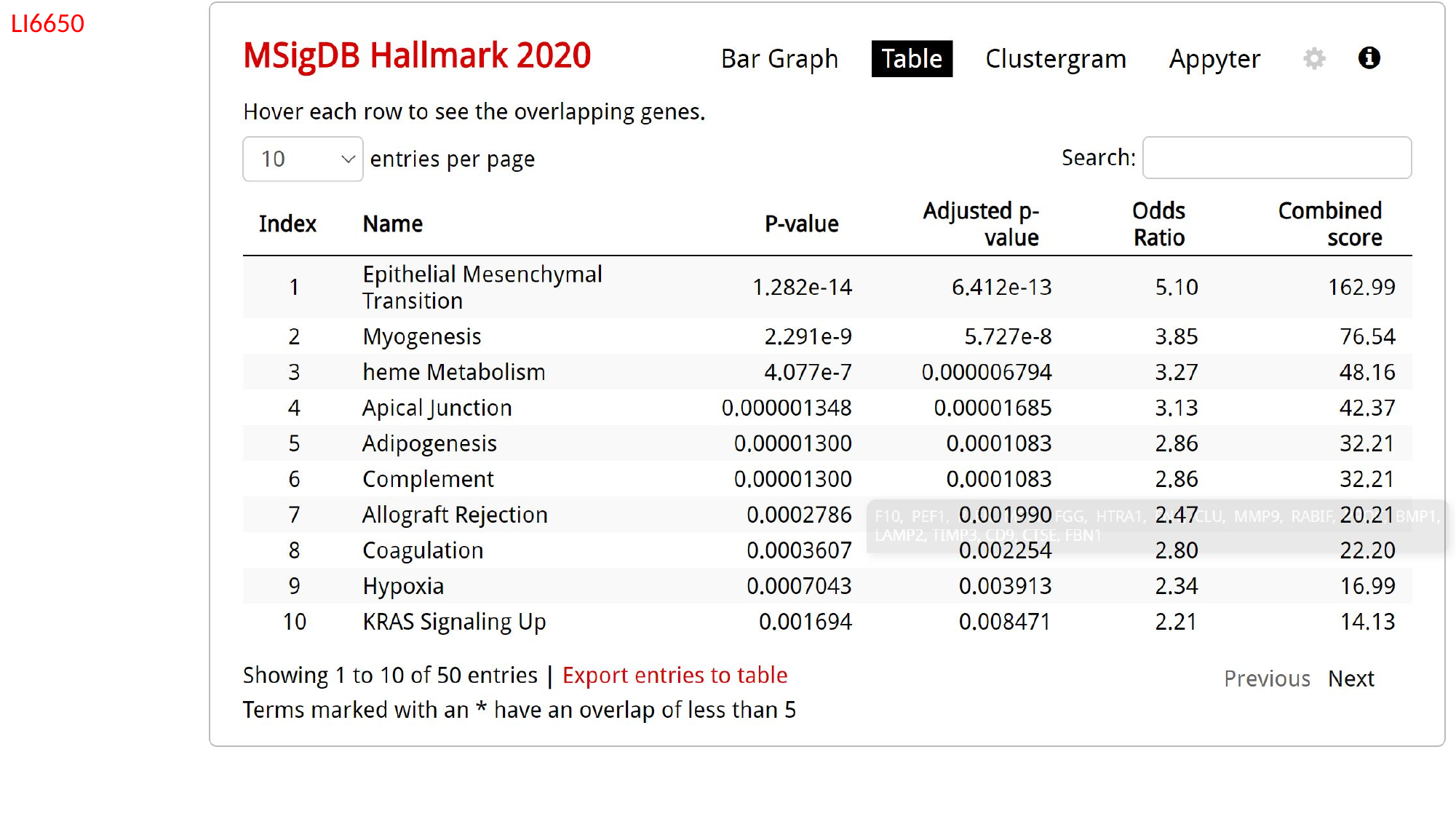

LI6650

## Slide 12
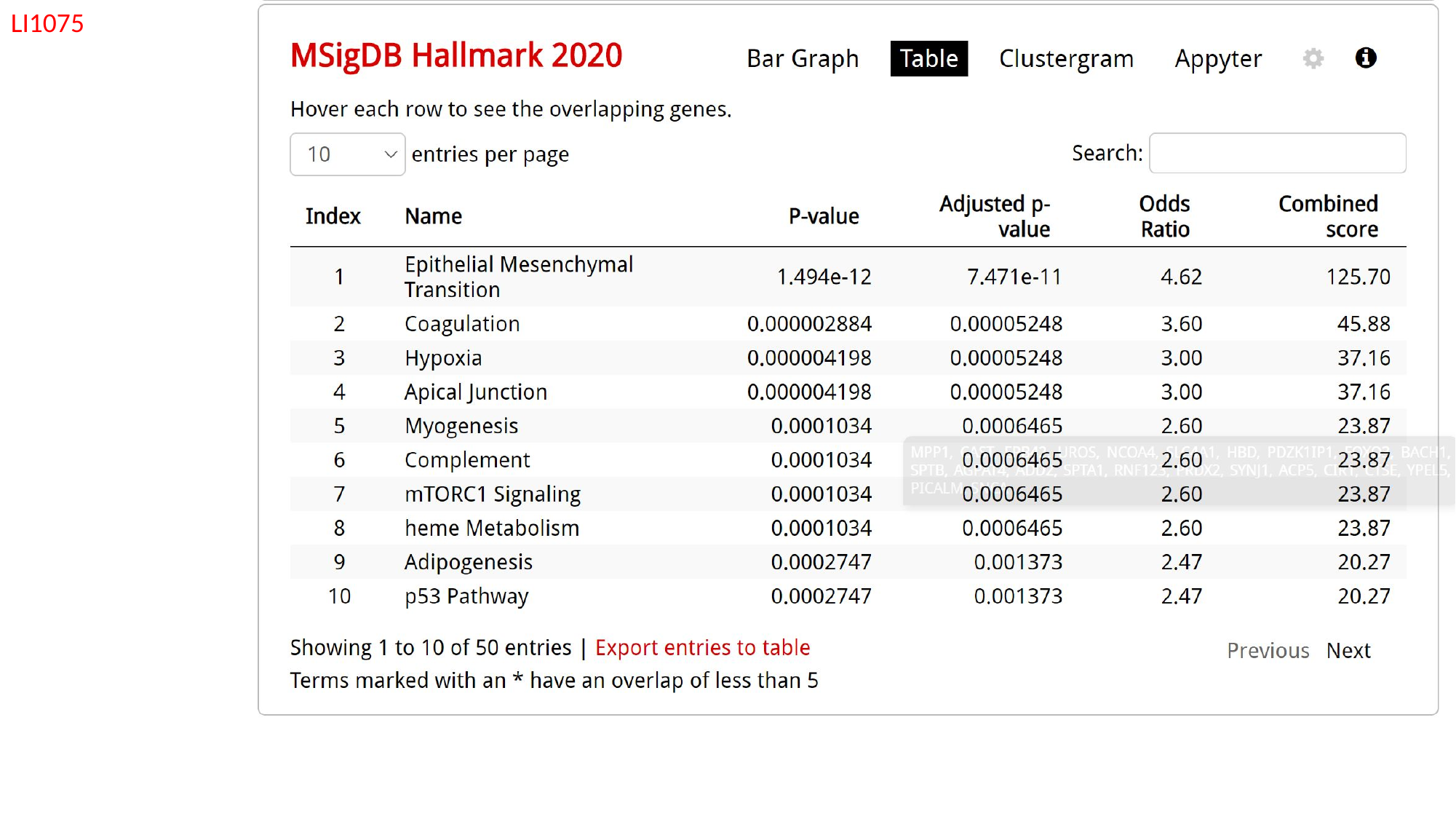

LI1075

## Slide 13
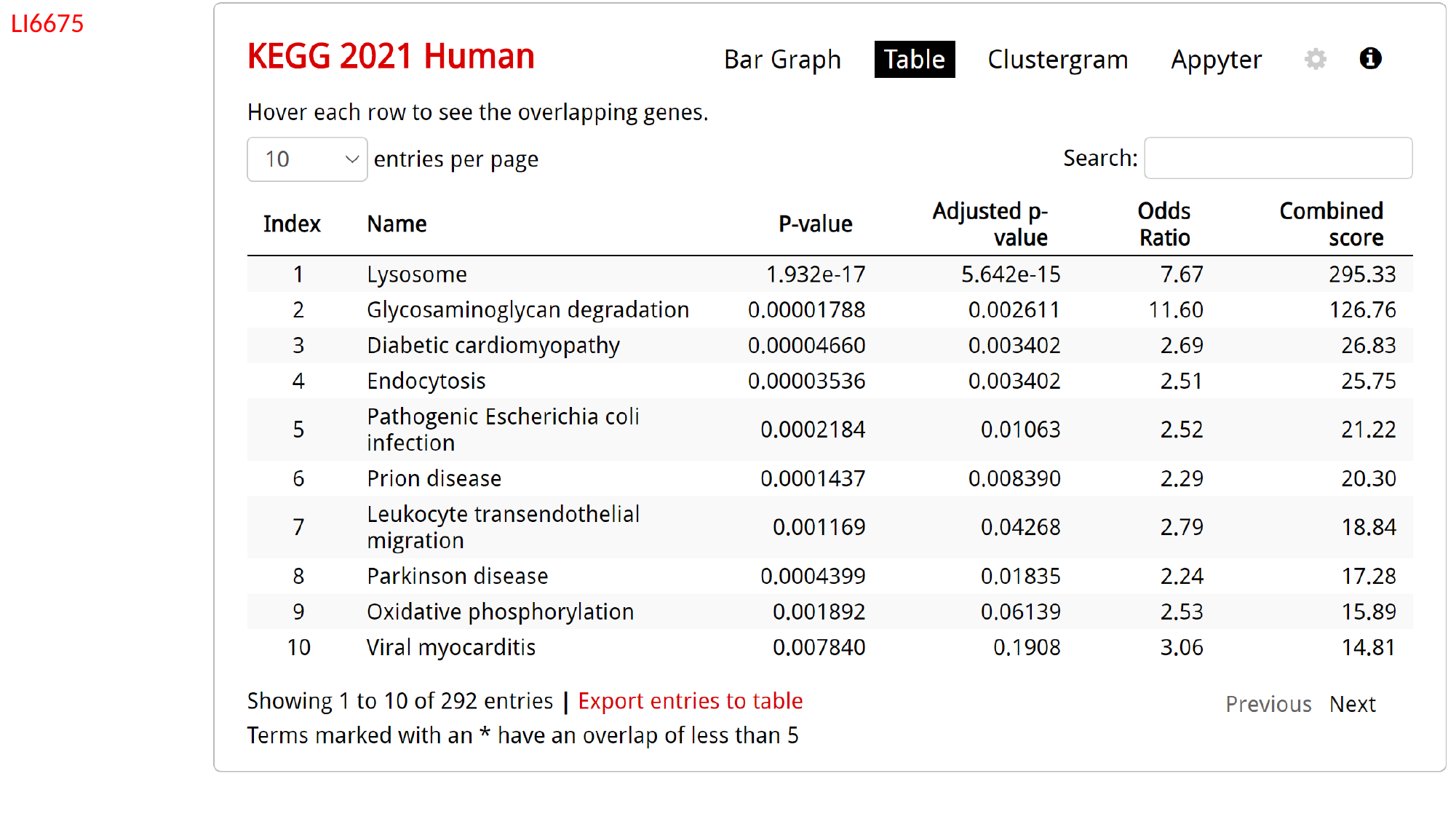

LI6675

## Slide 14
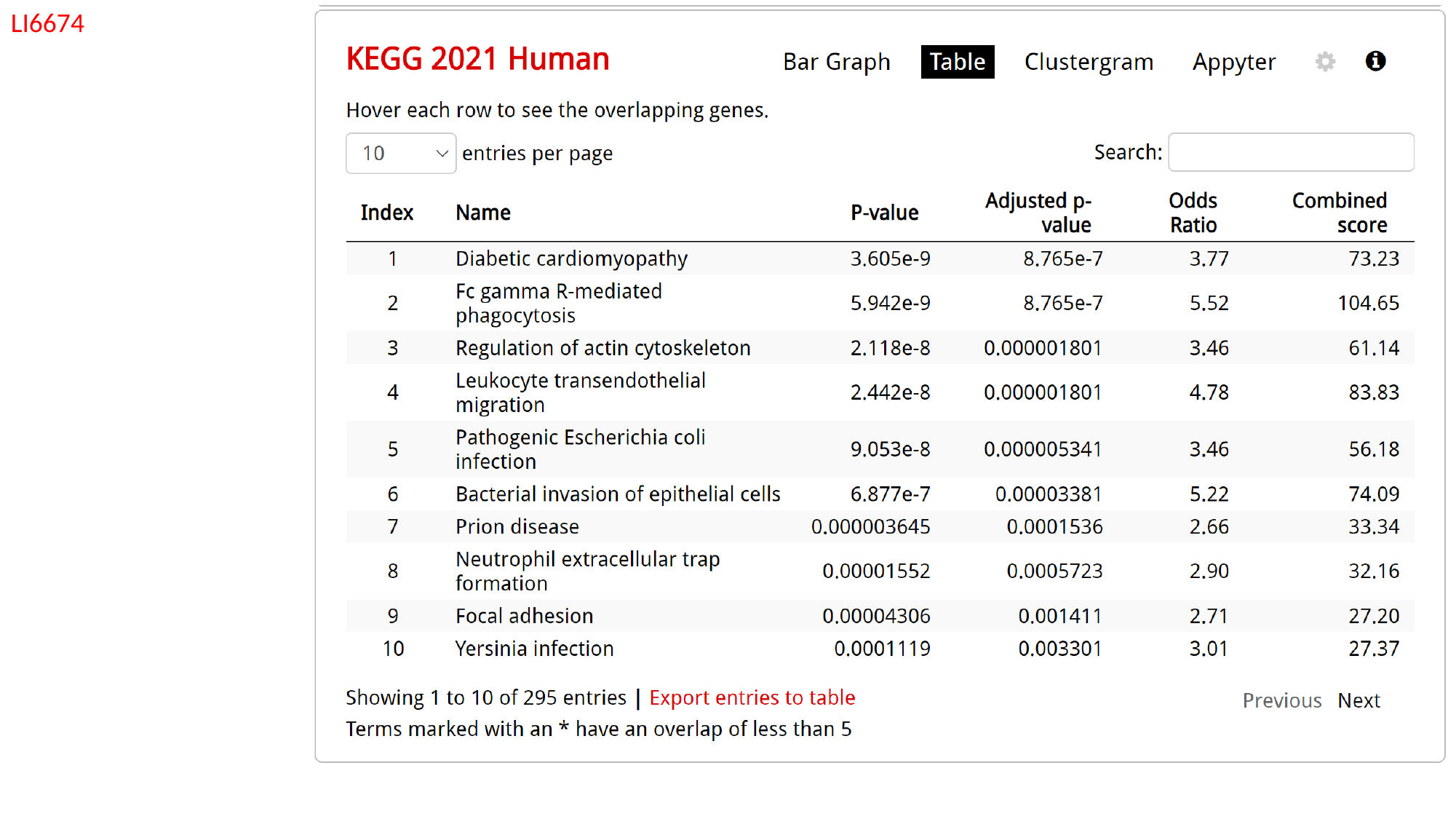

LI6674

## Slide 15
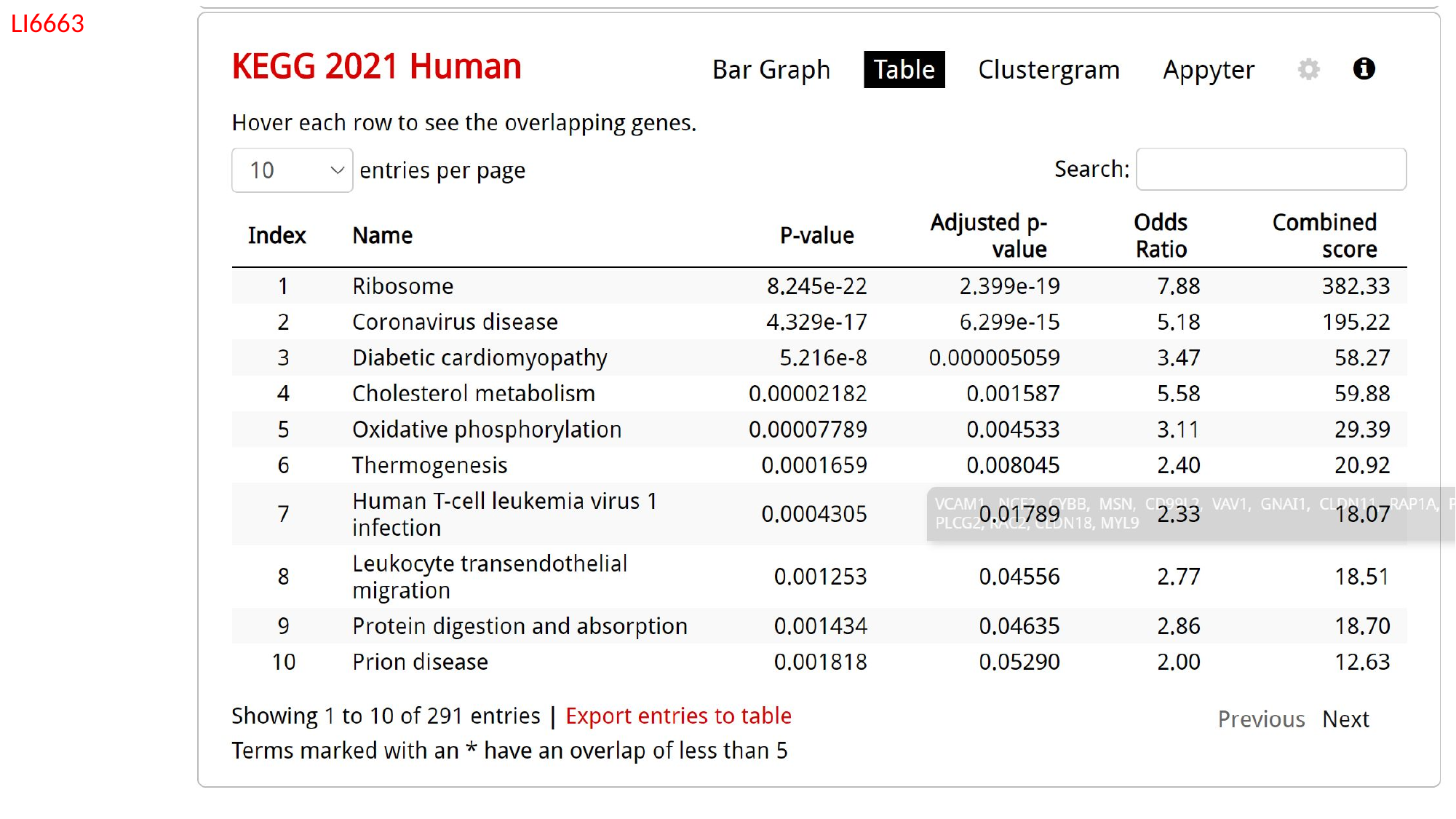

LI6663

## Slide 16
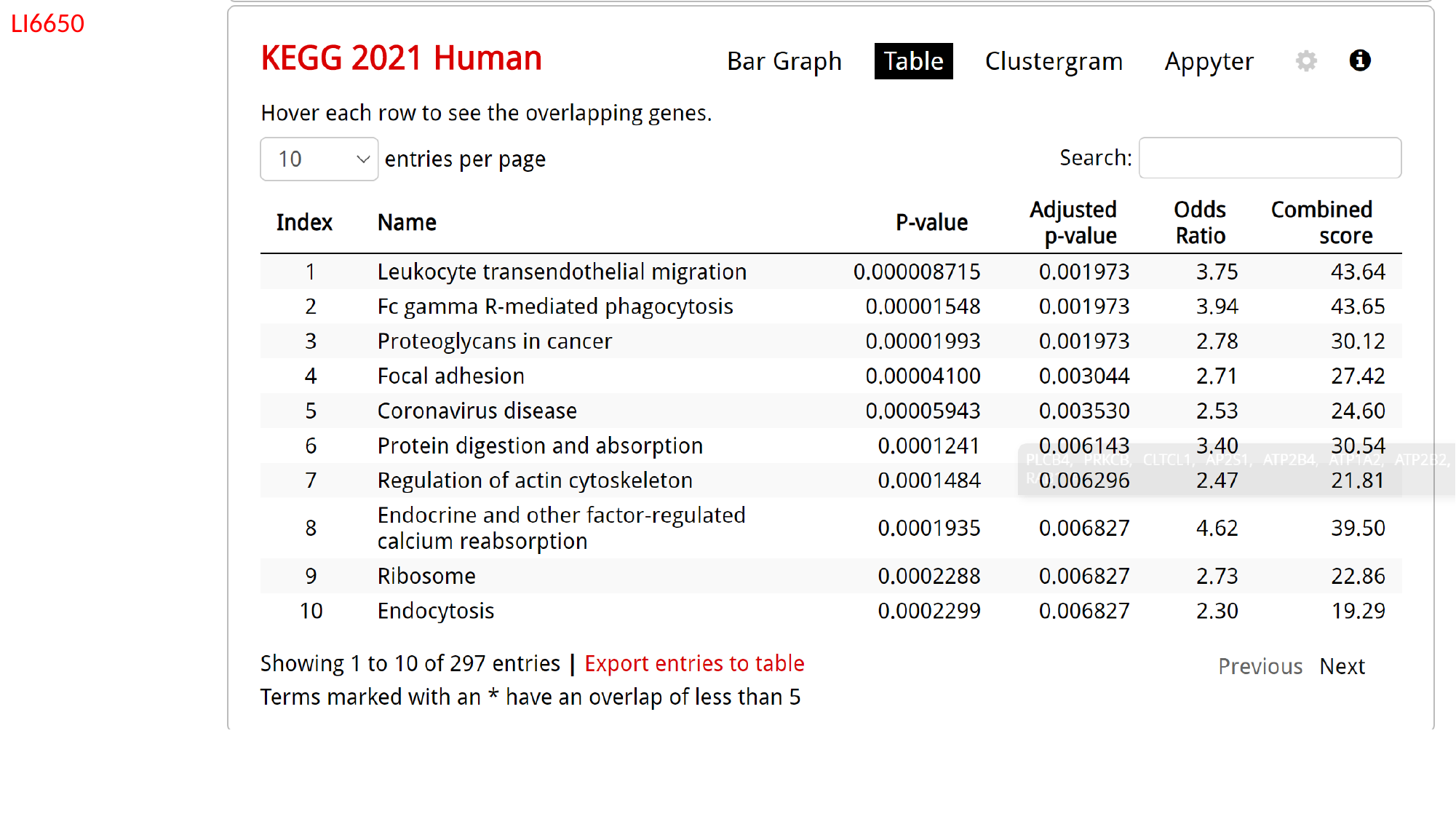

LI6650

## Slide 17
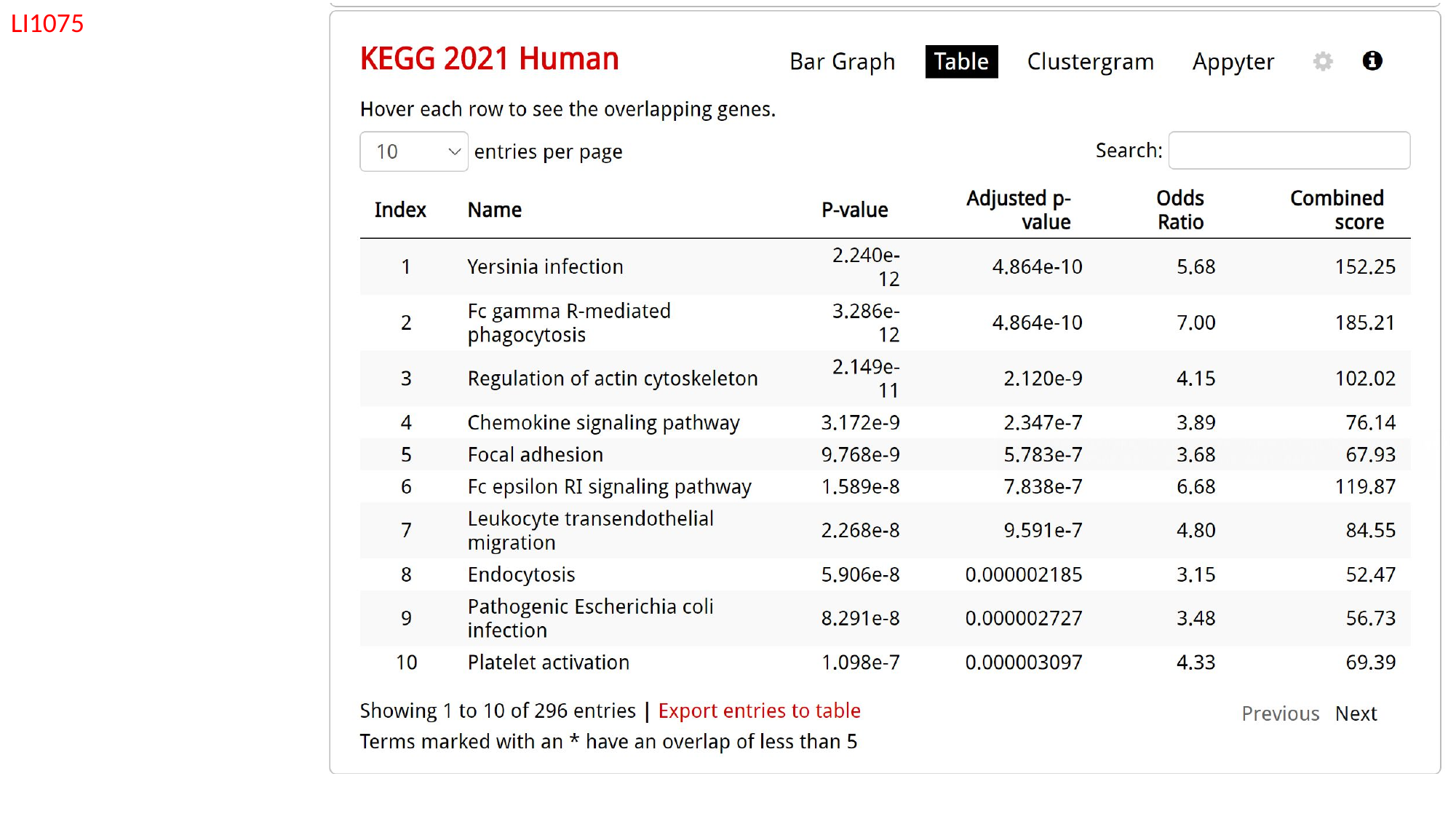

LI1075

## Slide 18
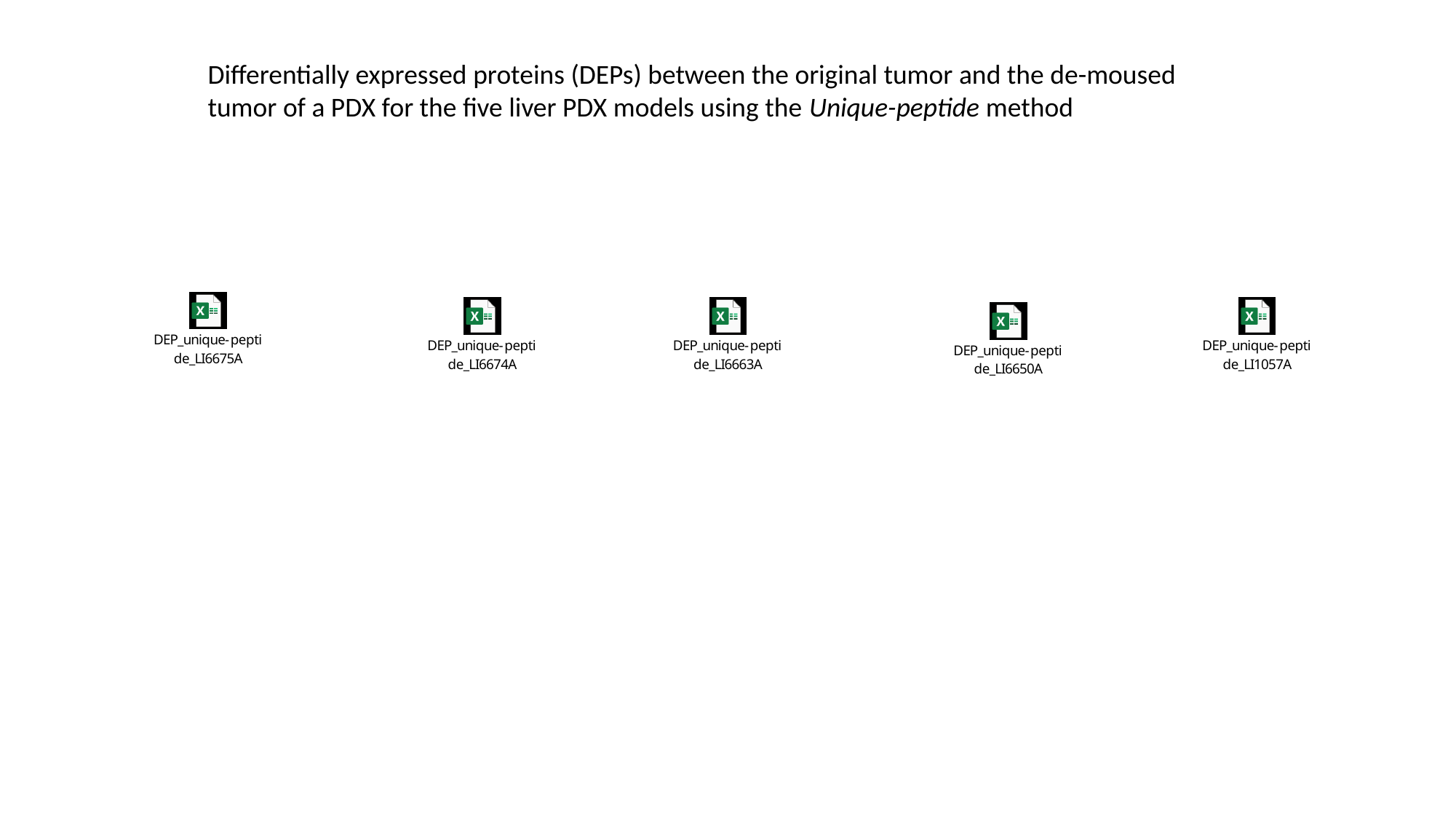

Differentially expressed proteins (DEPs) between the original tumor and the de-moused tumor of a PDX for the five liver PDX models using the Unique-peptide method

## Slide 19
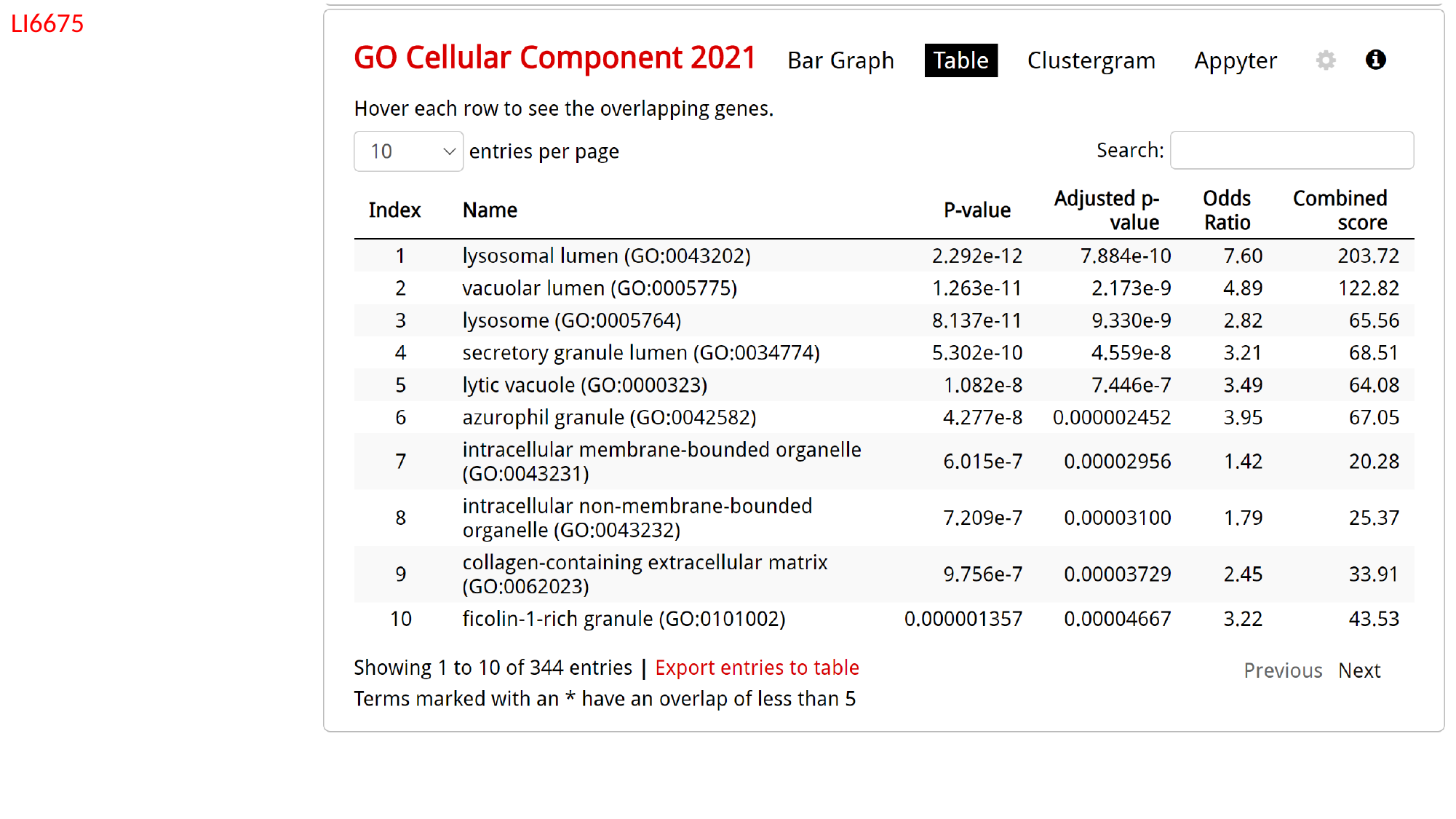

LI6675

## Slide 20
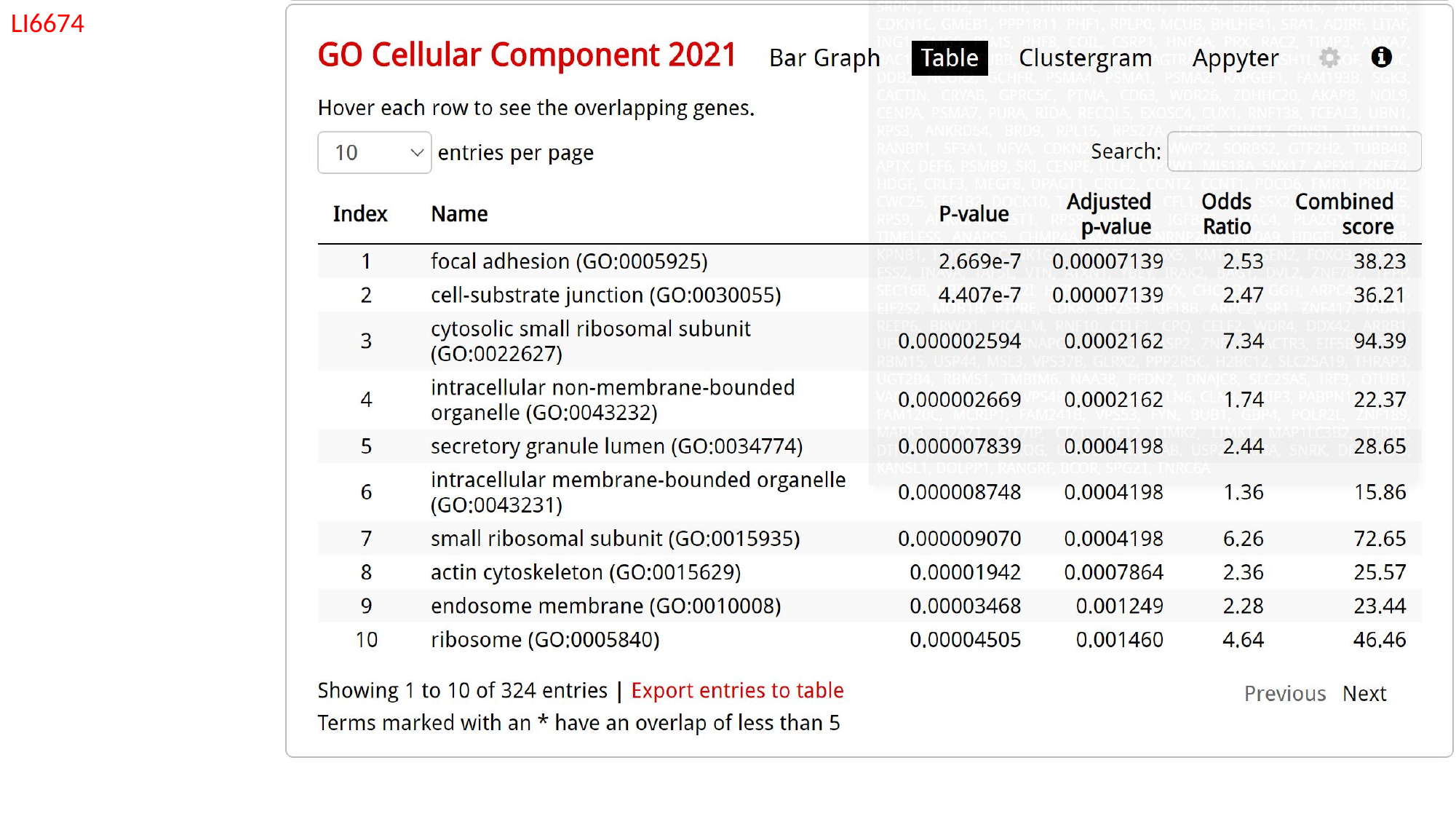

LI6674

## Slide 21
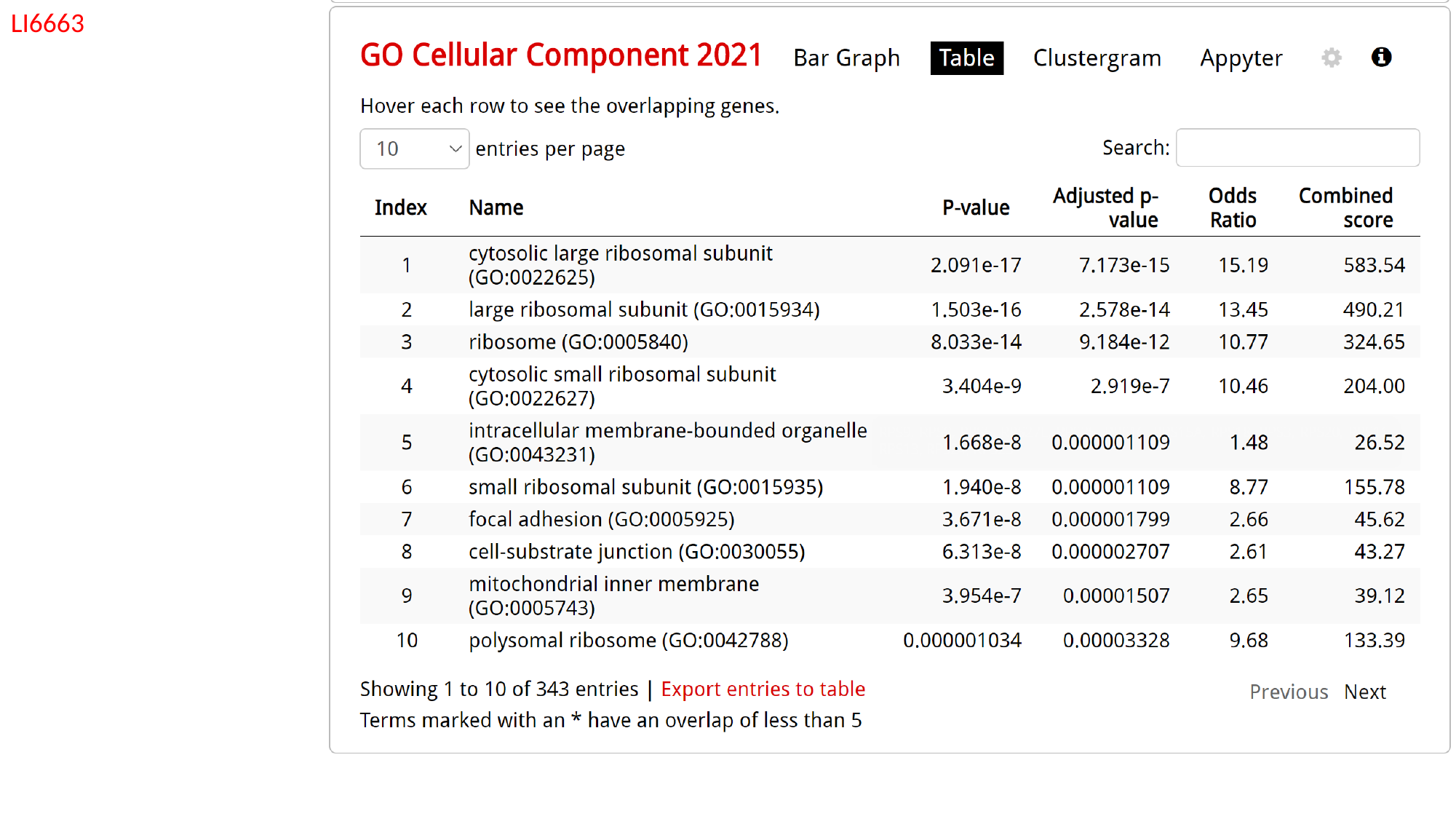

LI6663

## Slide 22
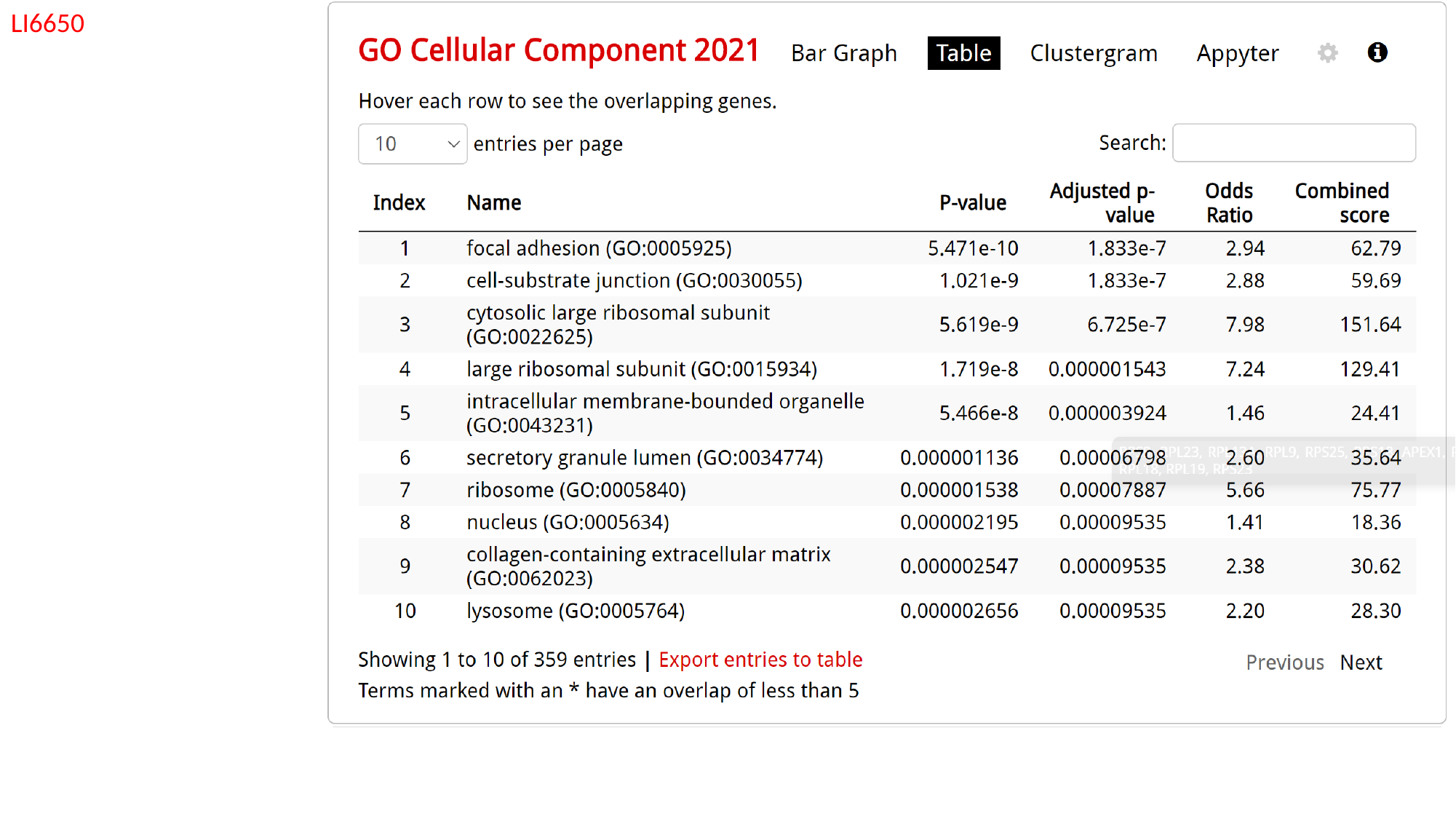

LI6650

## Slide 23
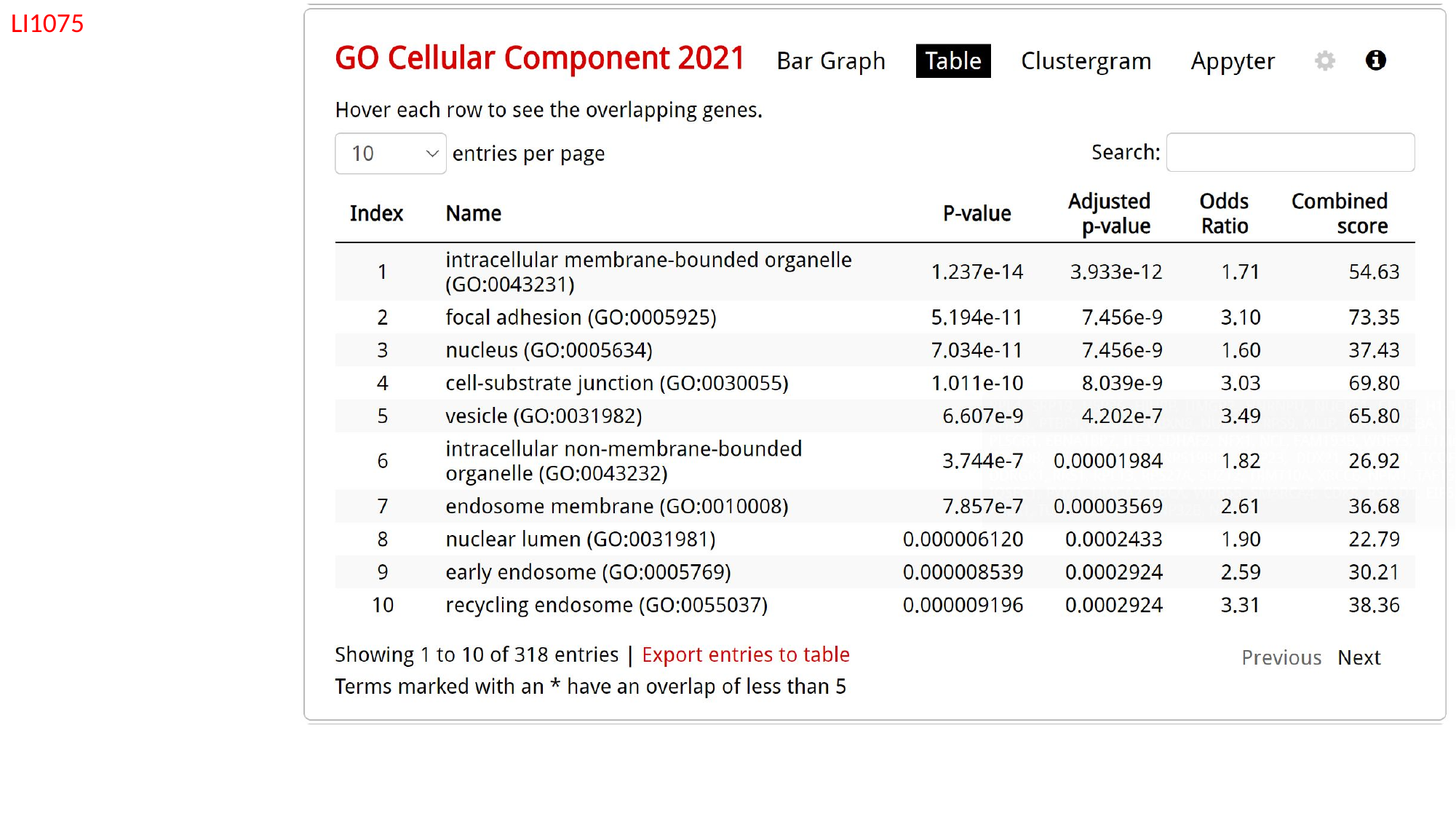

LI1075

## Slide 24
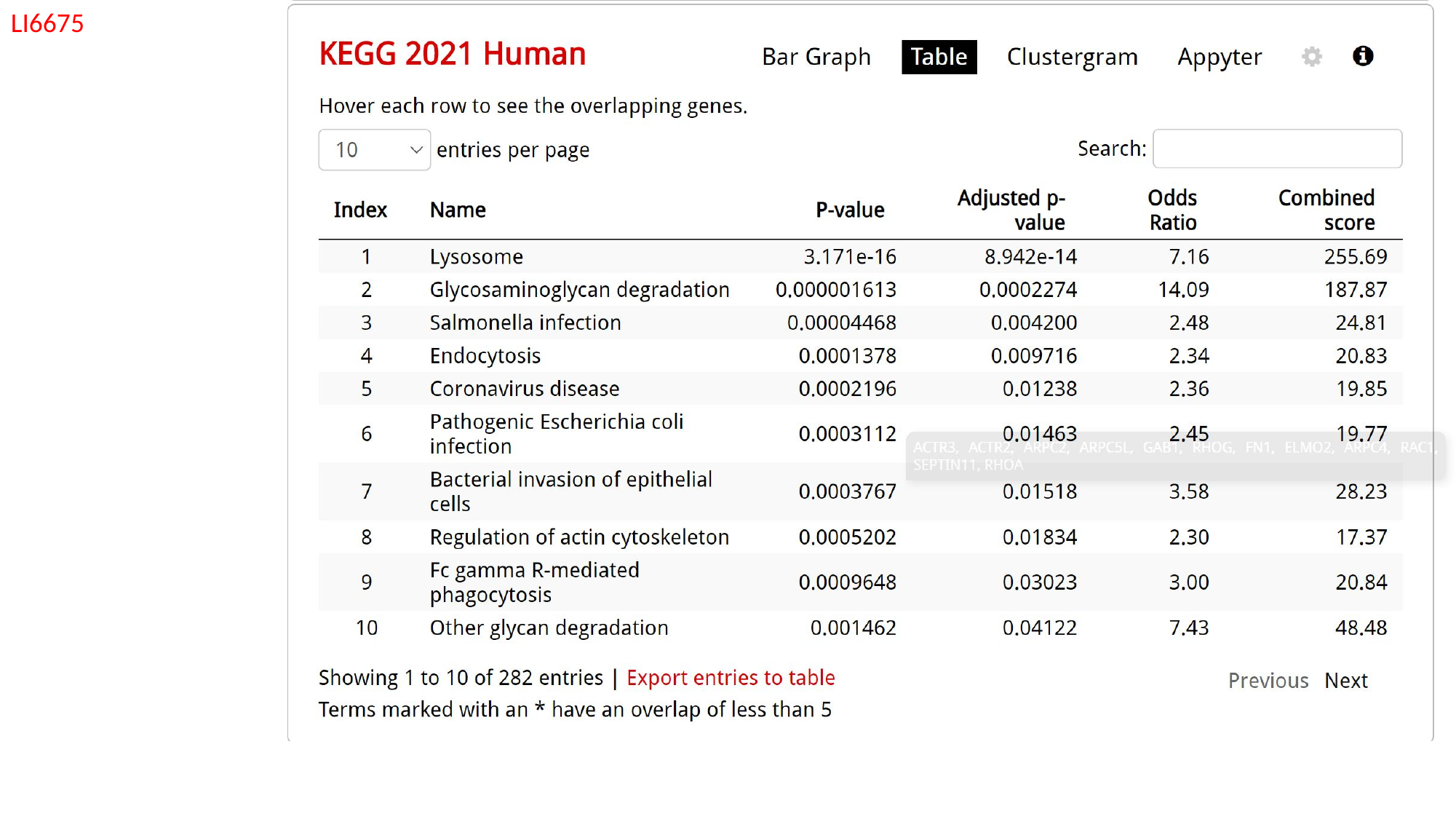

LI6675

## Slide 25
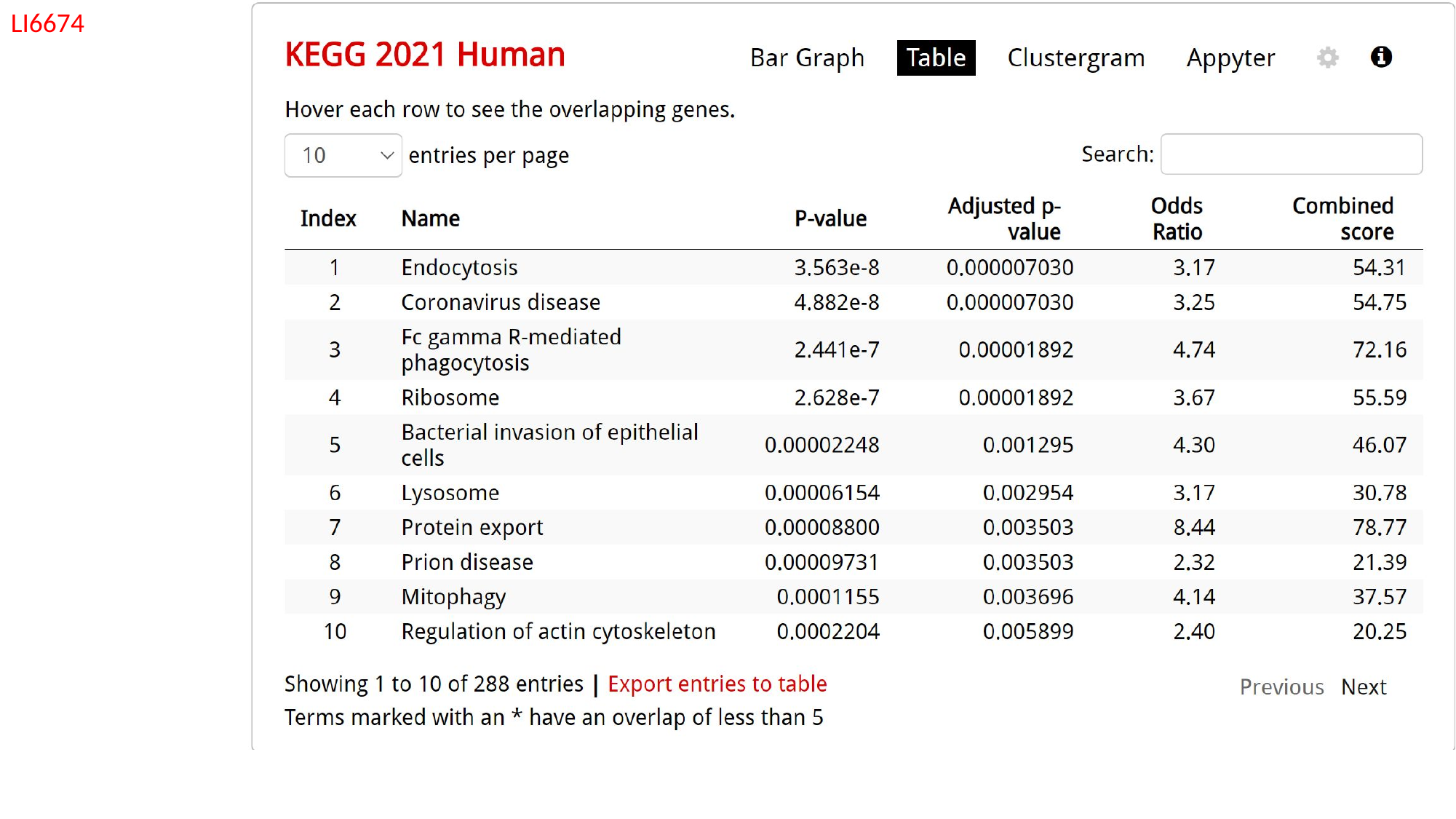

LI6674

## Slide 26
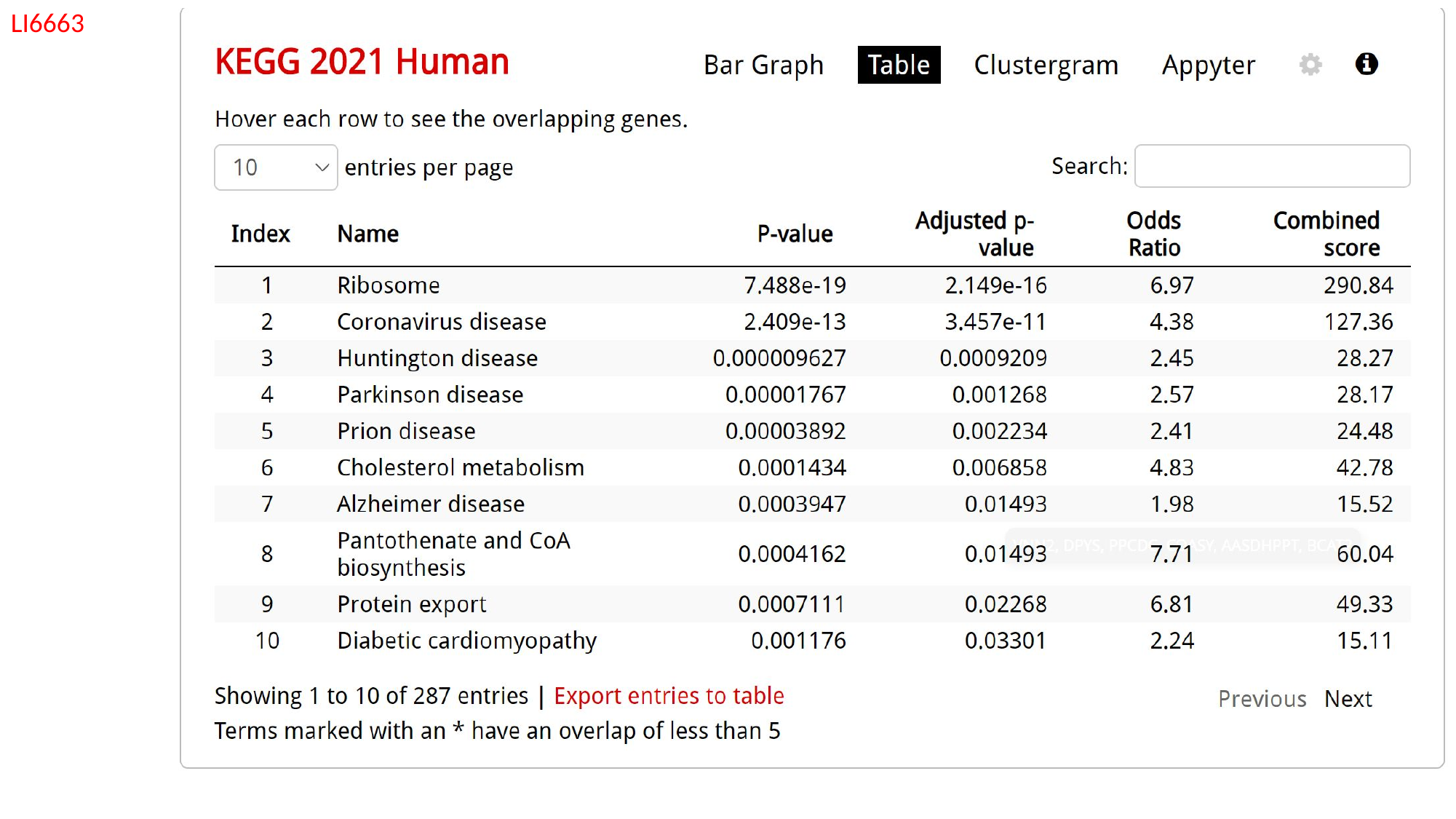

LI6663

## Slide 27
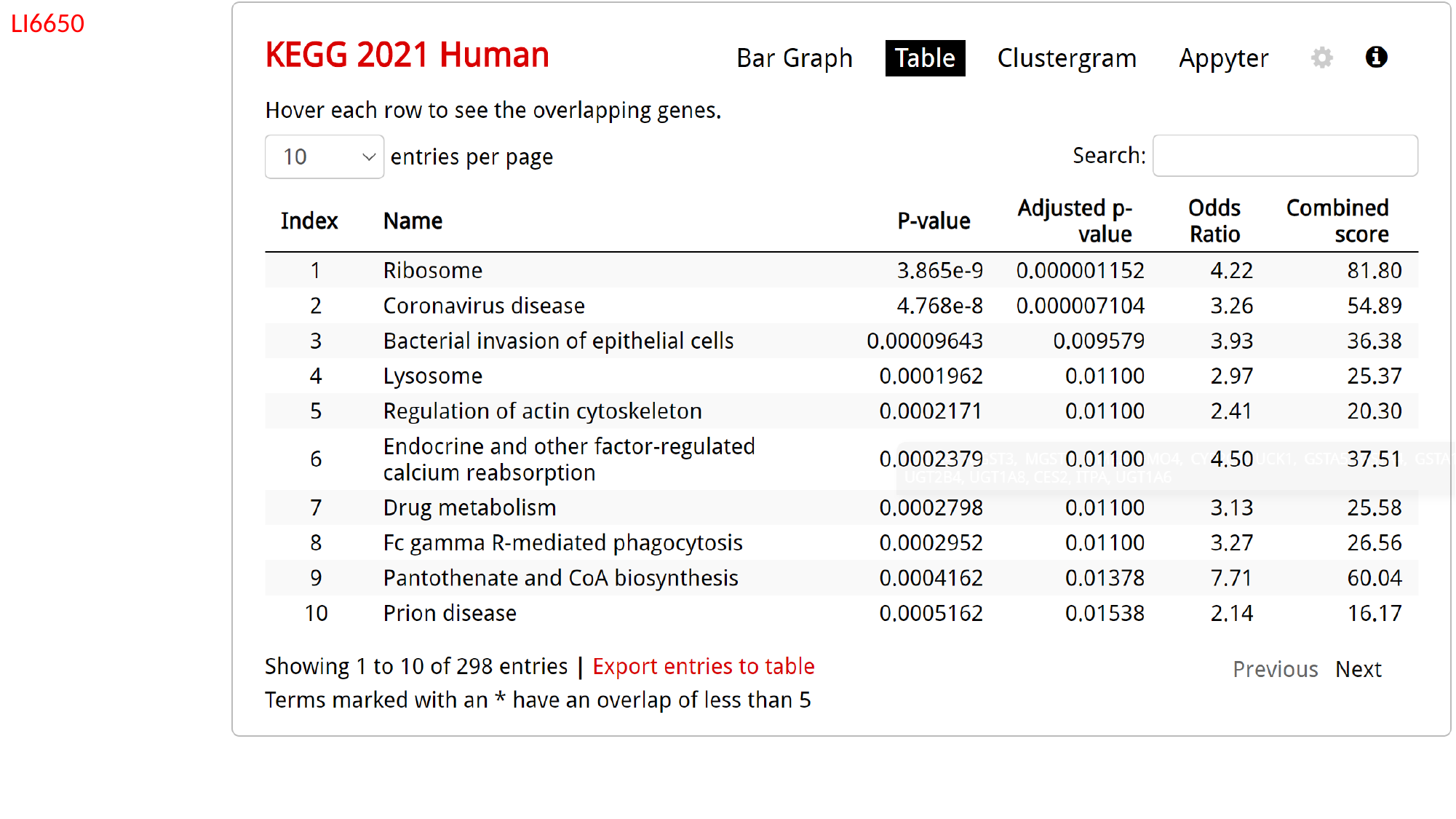

LI6650

## Slide 28
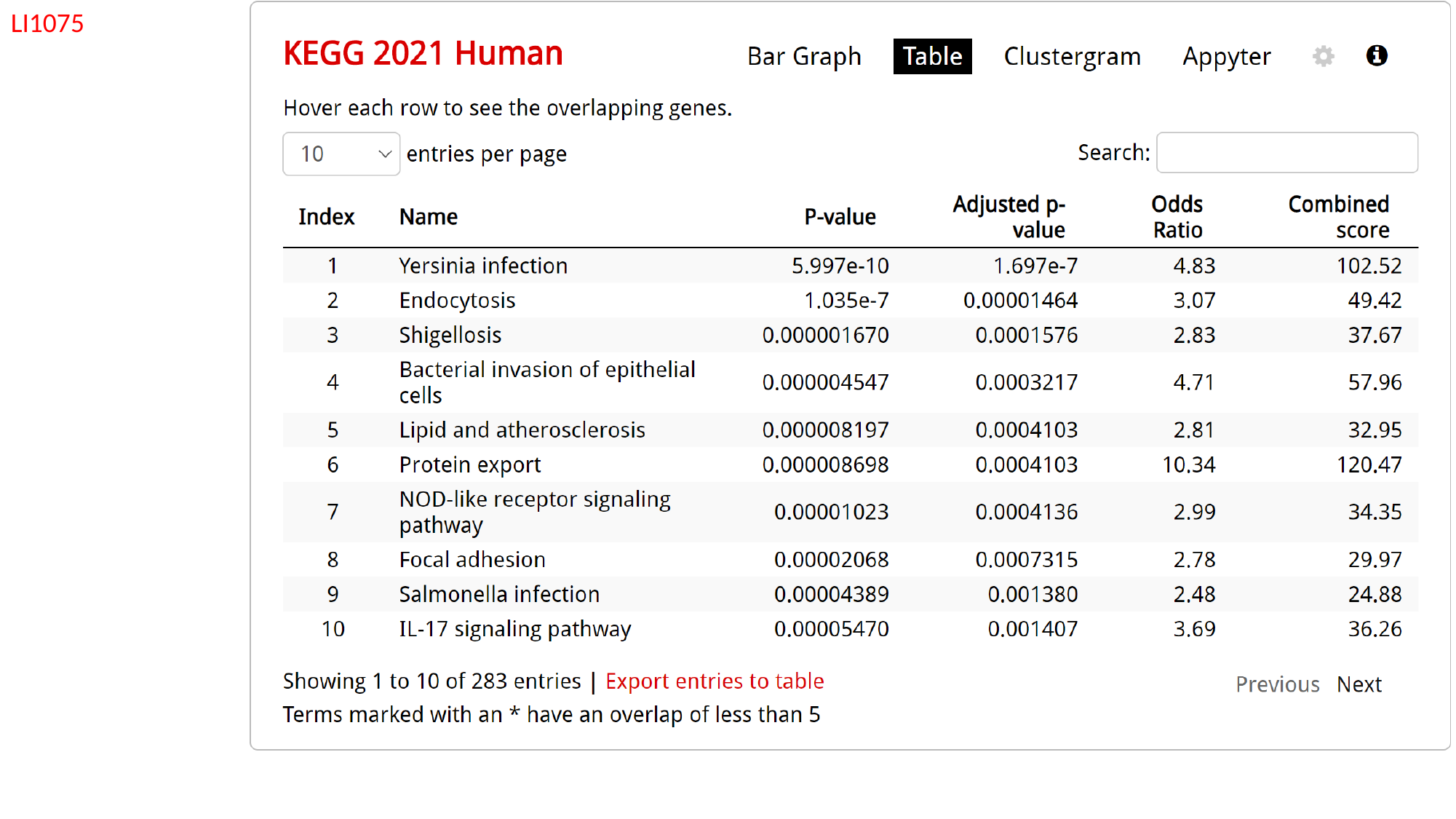

LI1075

## Slide 29
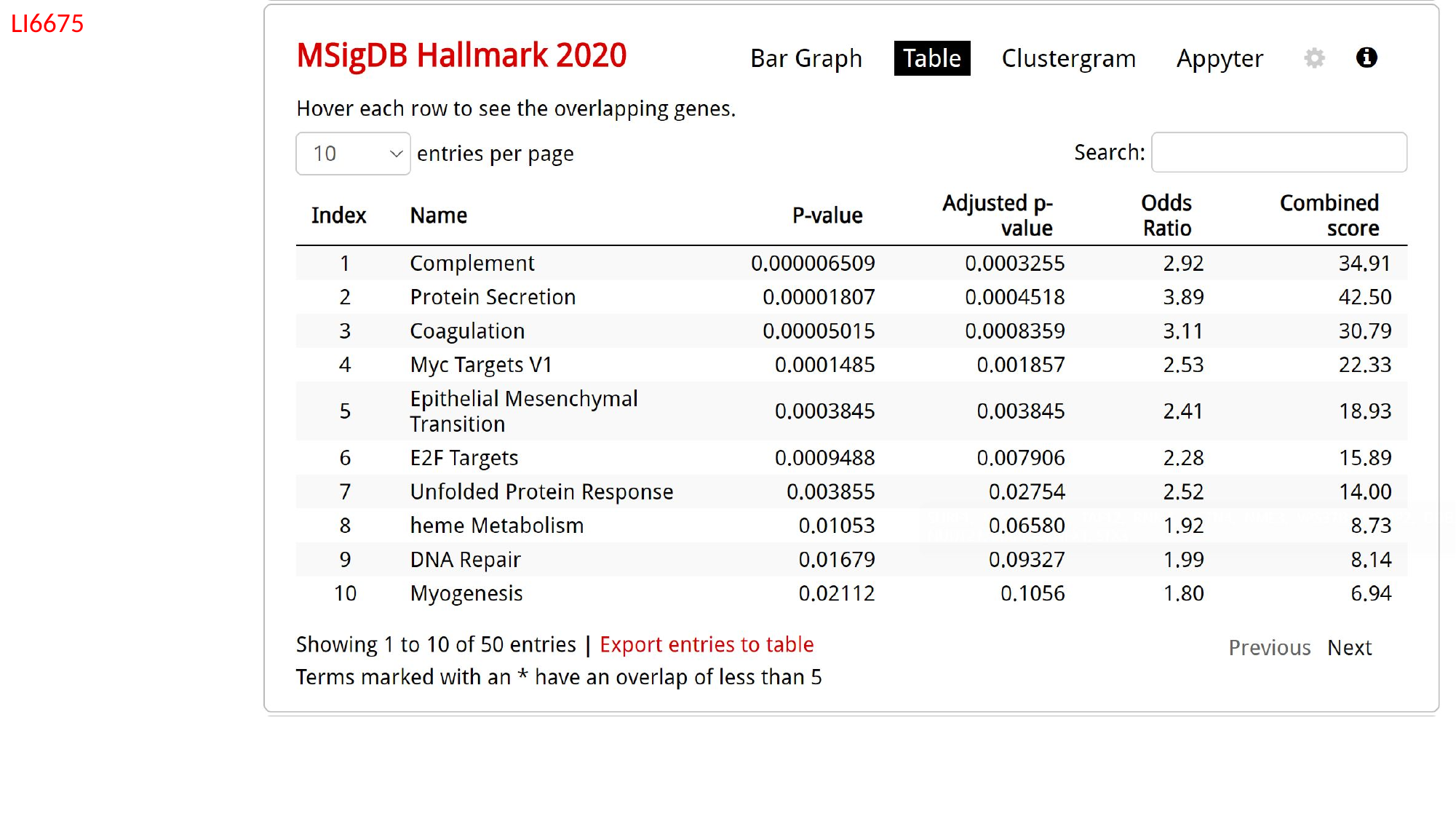

LI6675

## Slide 30
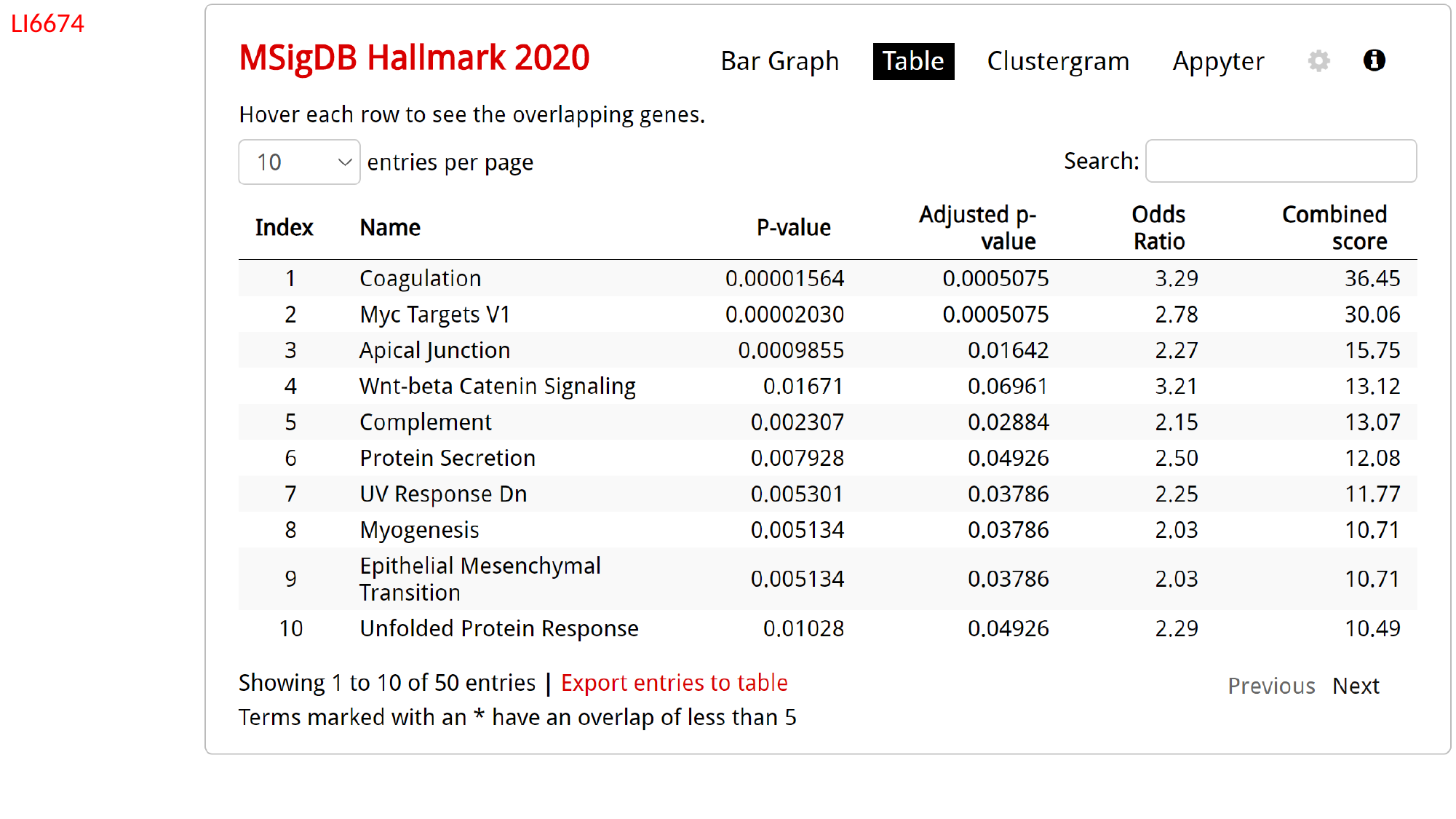

LI6674

## Slide 31
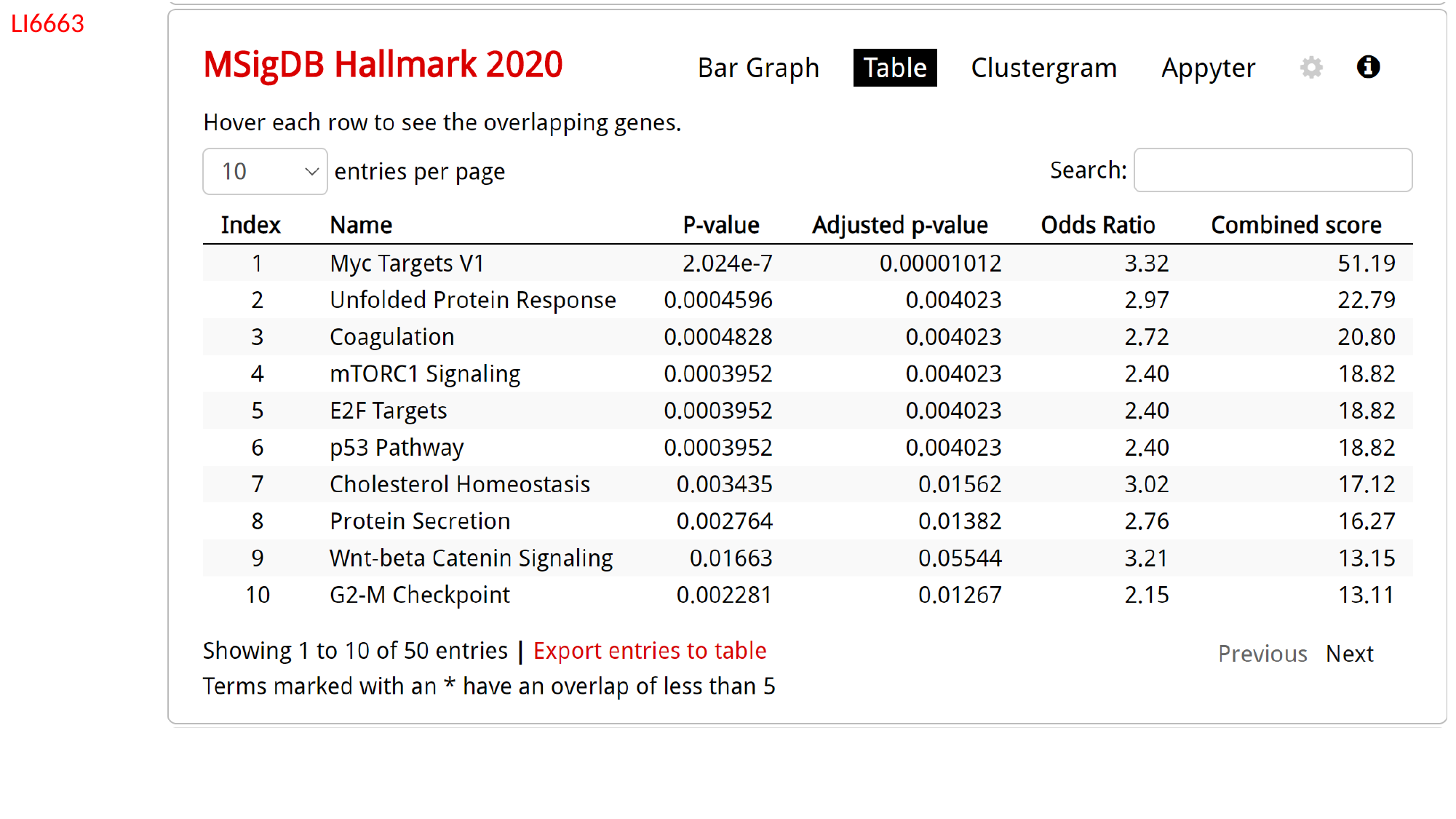

LI6663

## Slide 32
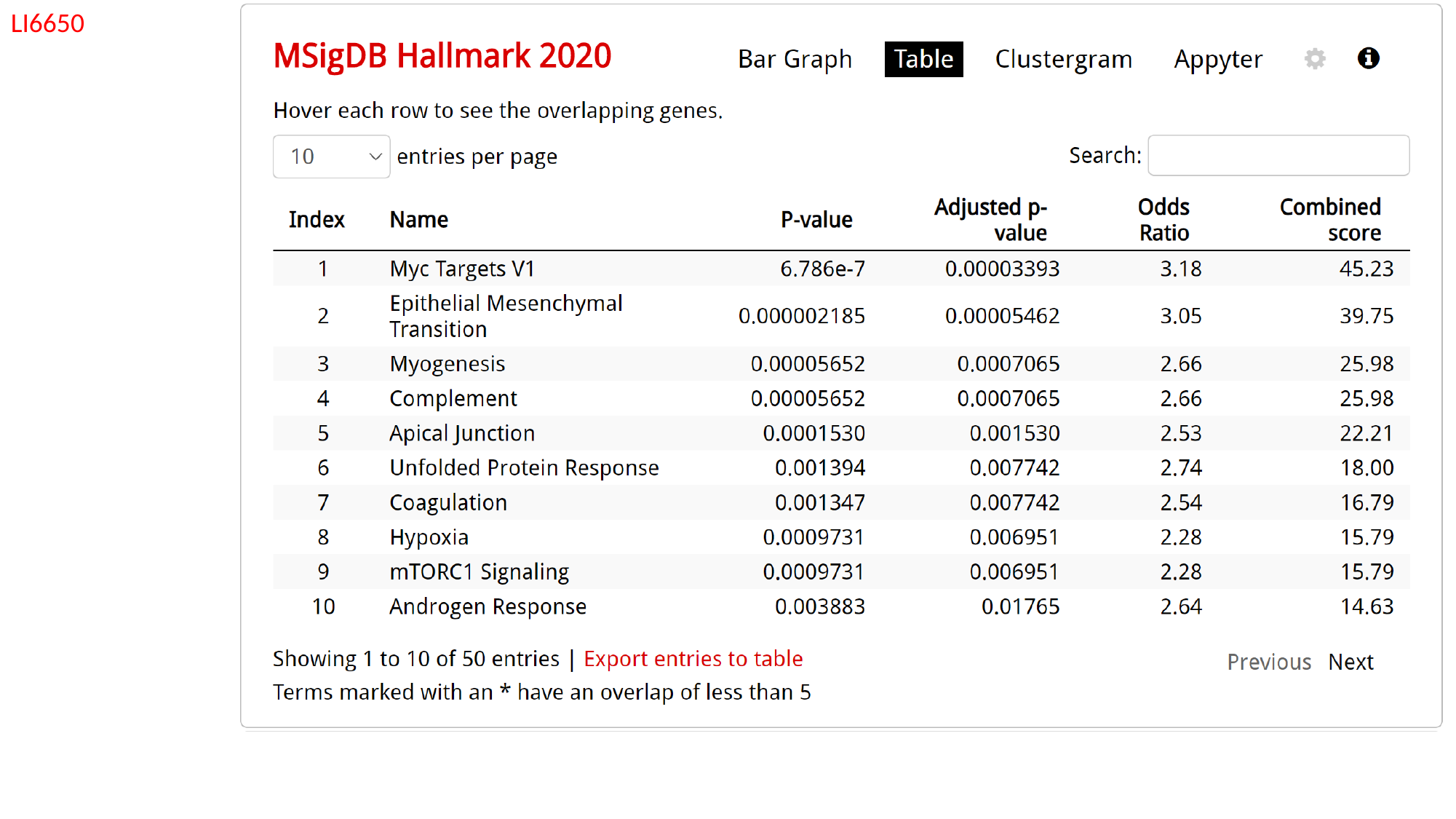

LI6650

## Slide 33
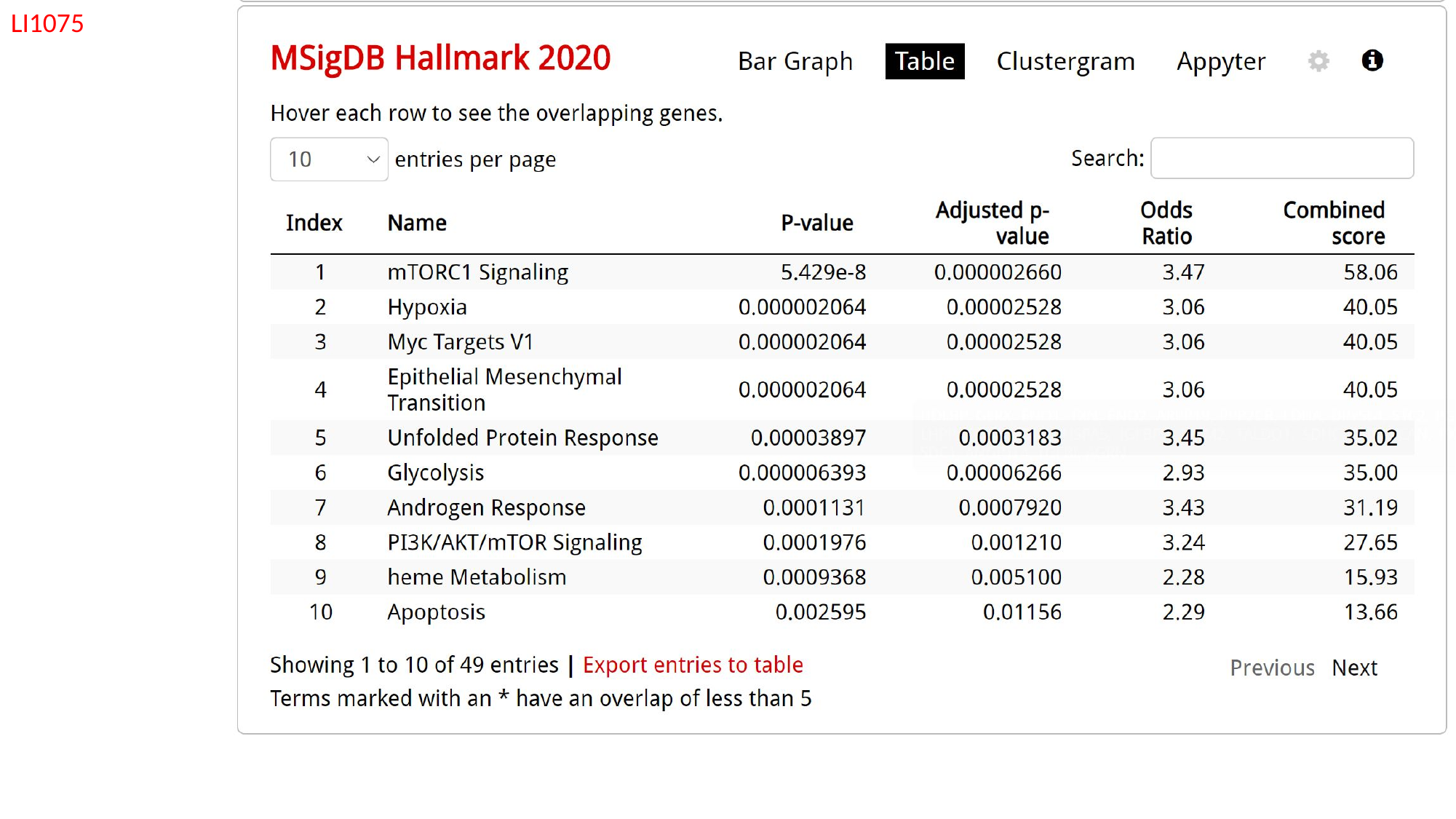

LI1075

## Slide 34
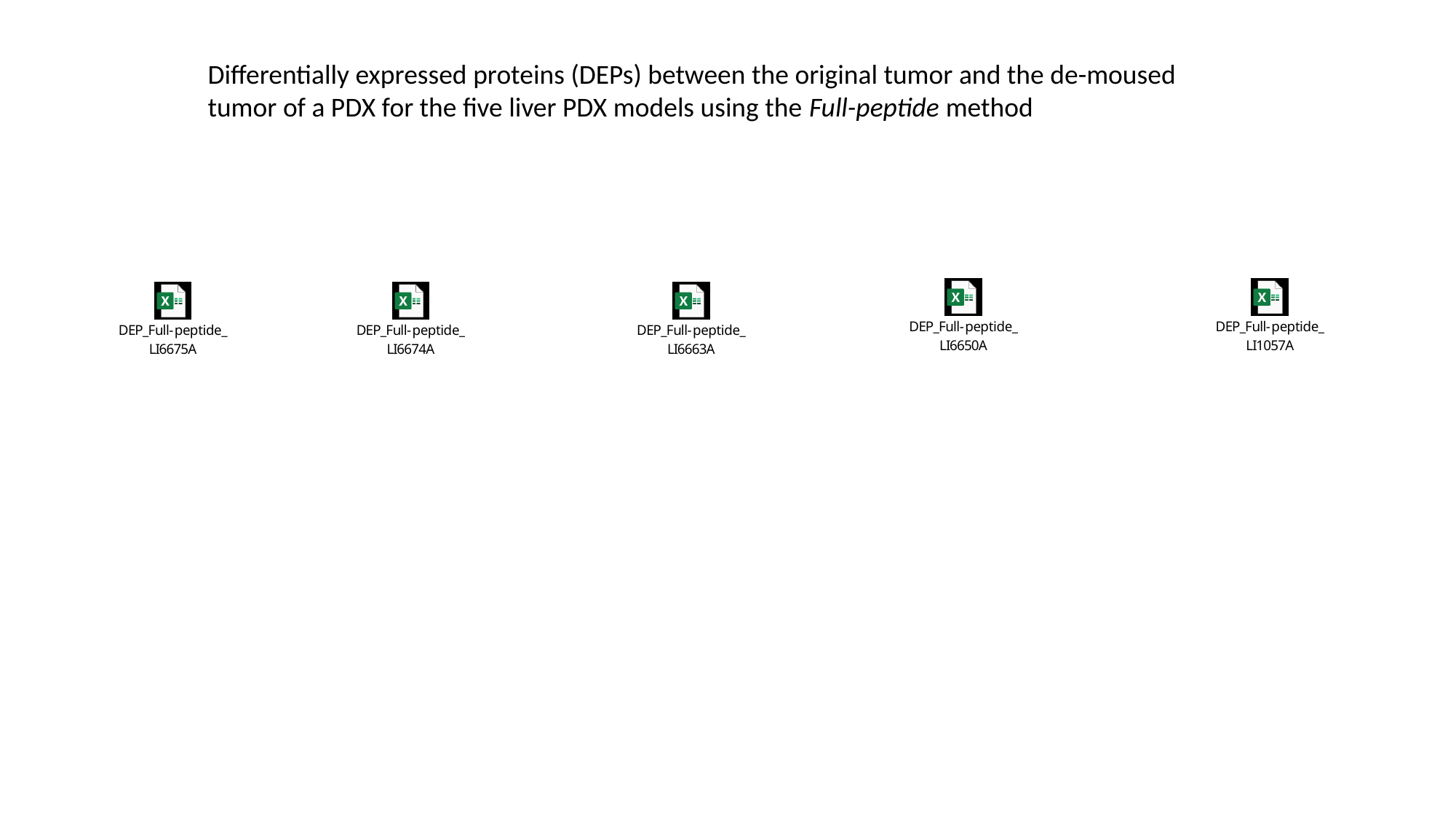

Differentially expressed proteins (DEPs) between the original tumor and the de-moused tumor of a PDX for the five liver PDX models using the Full-peptide method

## Slide 35
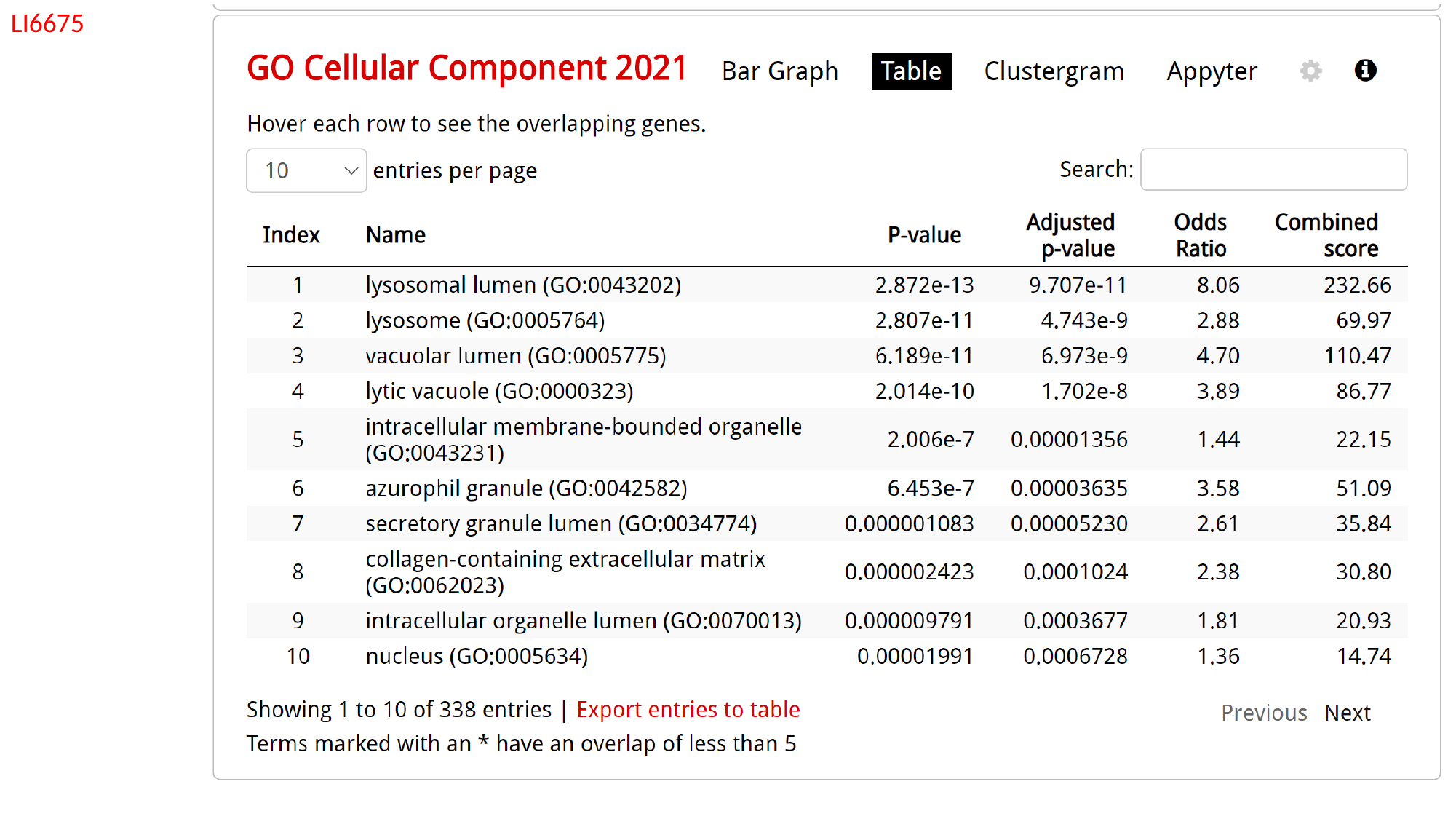

LI6675

## Slide 36
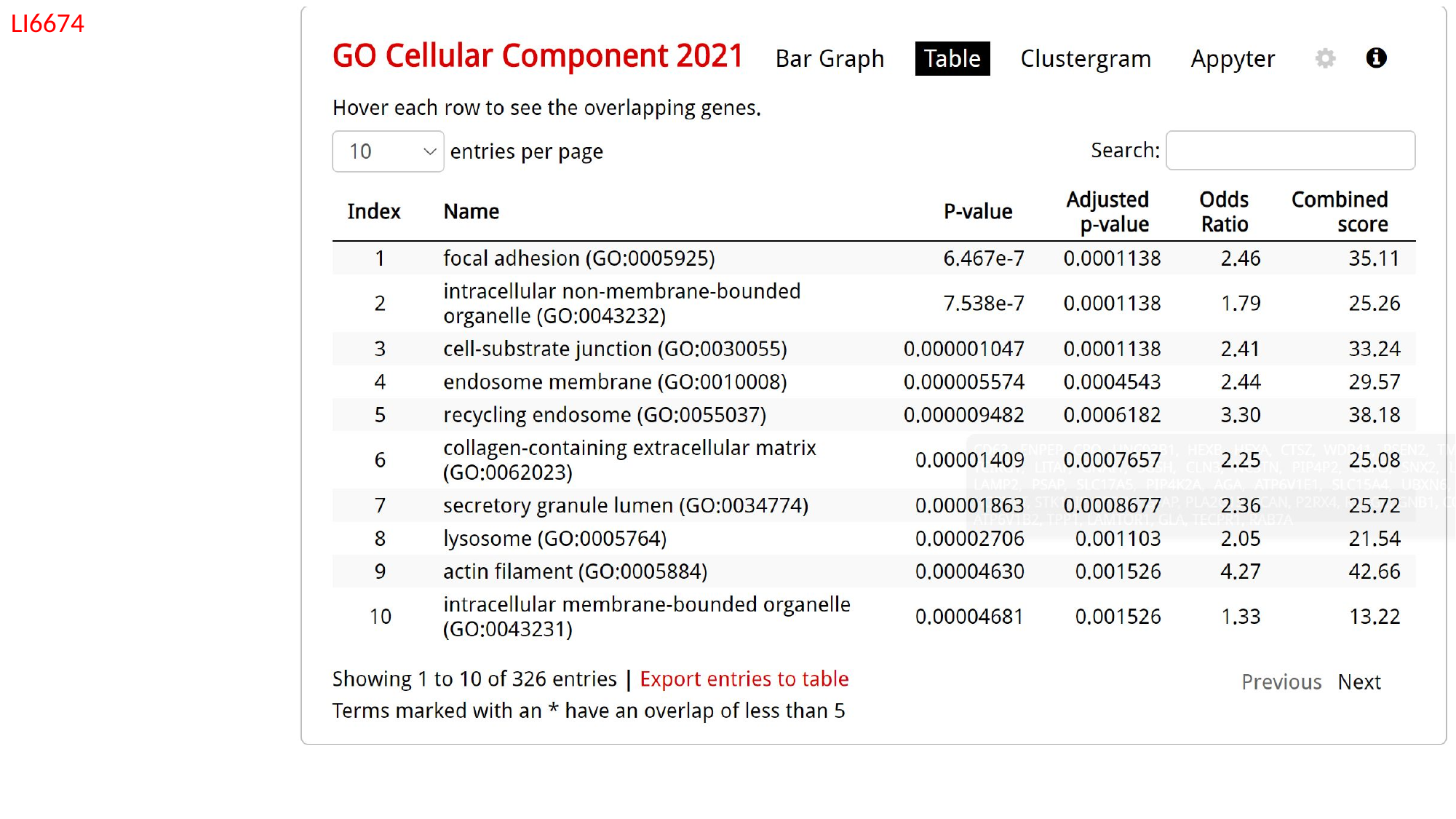

LI6674

## Slide 37
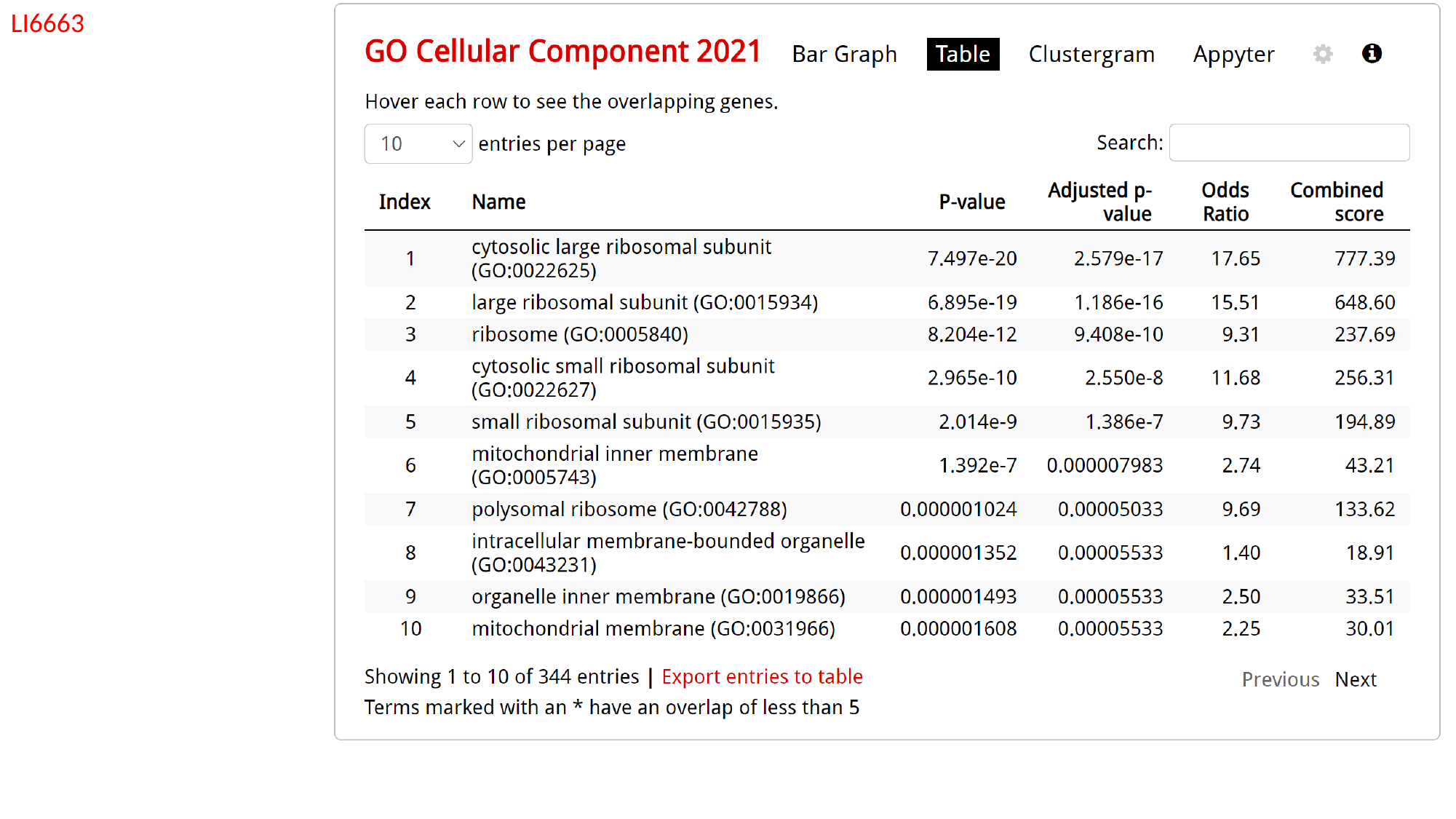

LI6663

## Slide 38
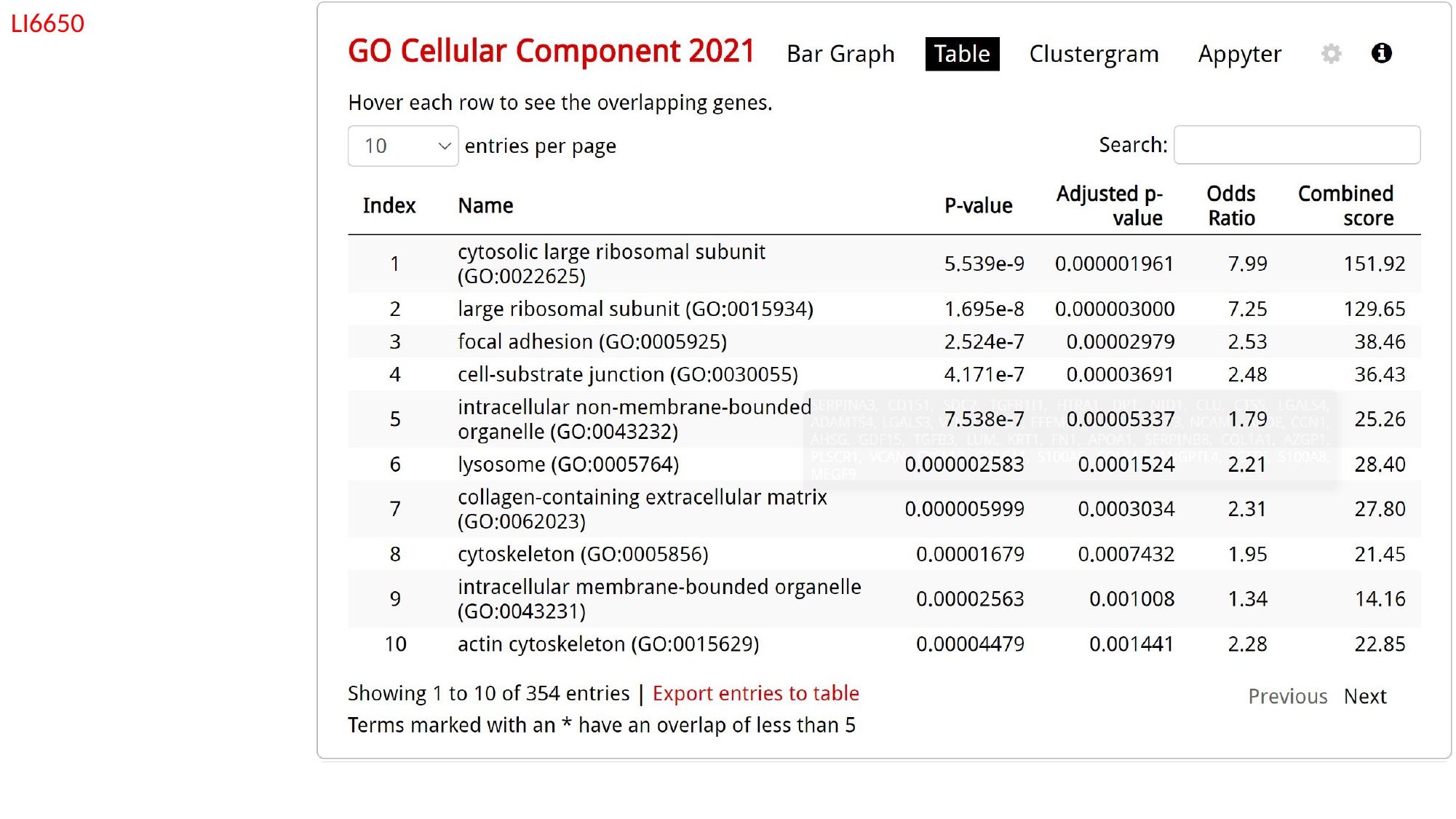

LI6650

## Slide 39
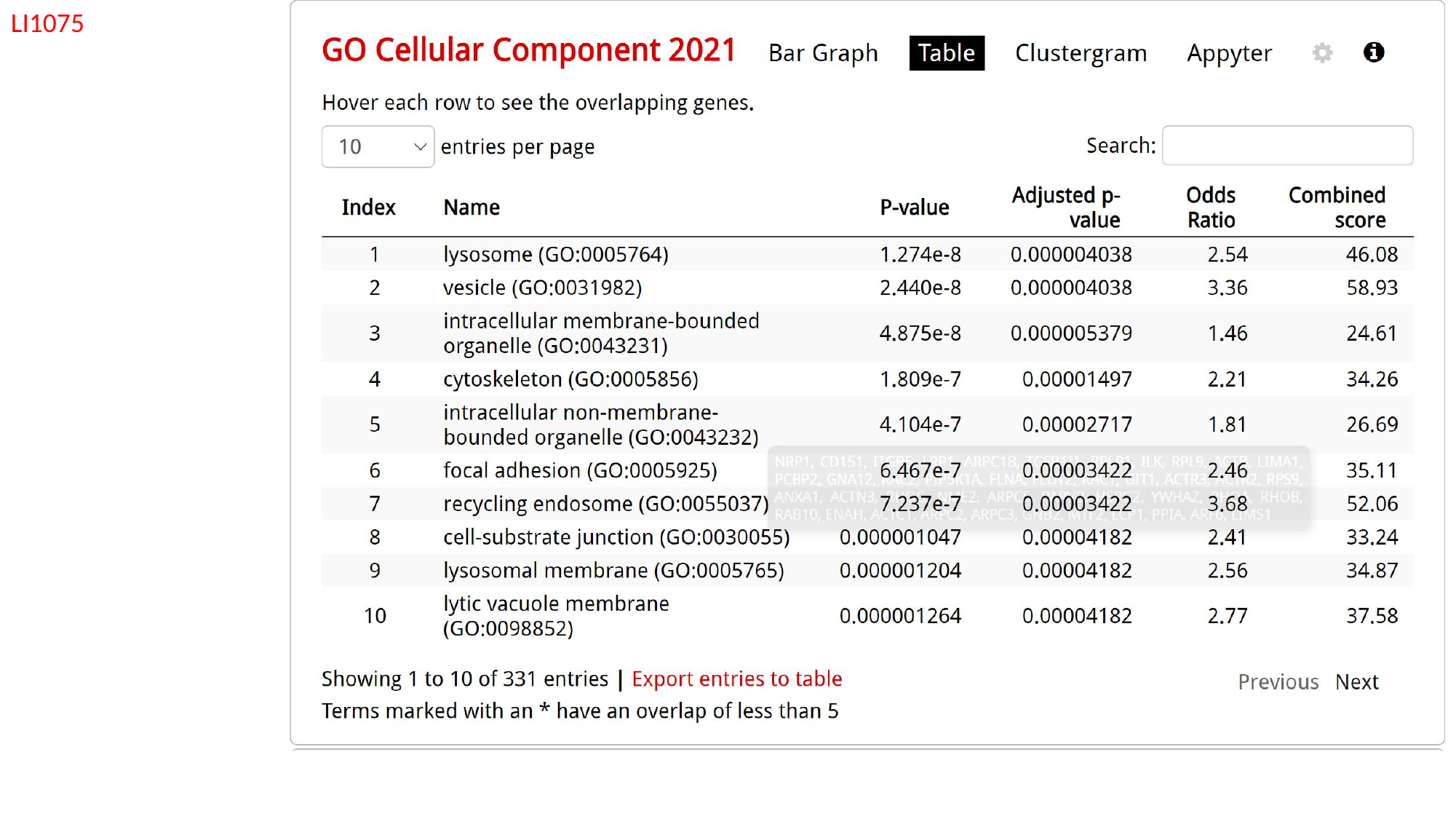

LI1075

## Slide 40
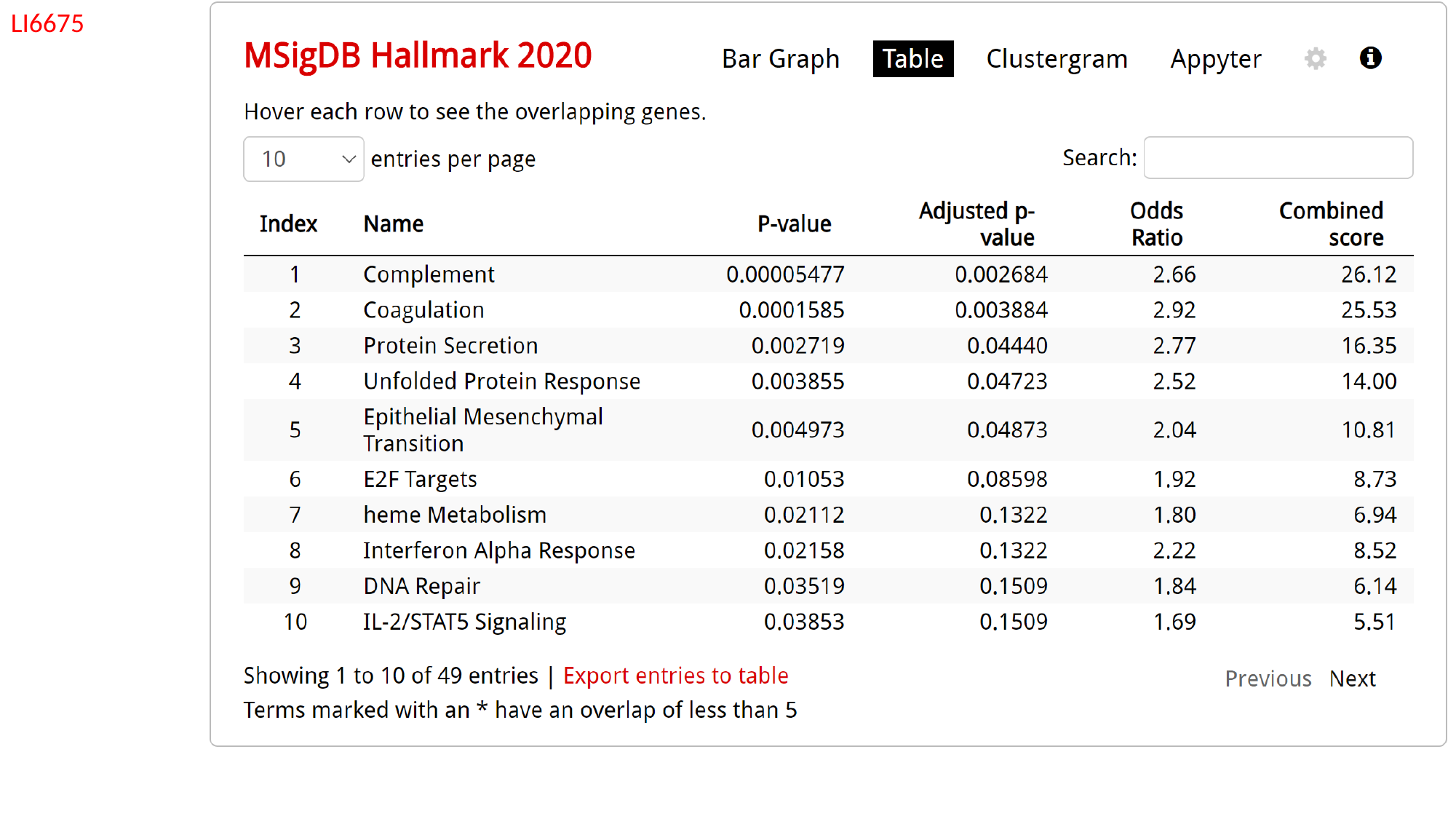

LI6675

## Slide 41
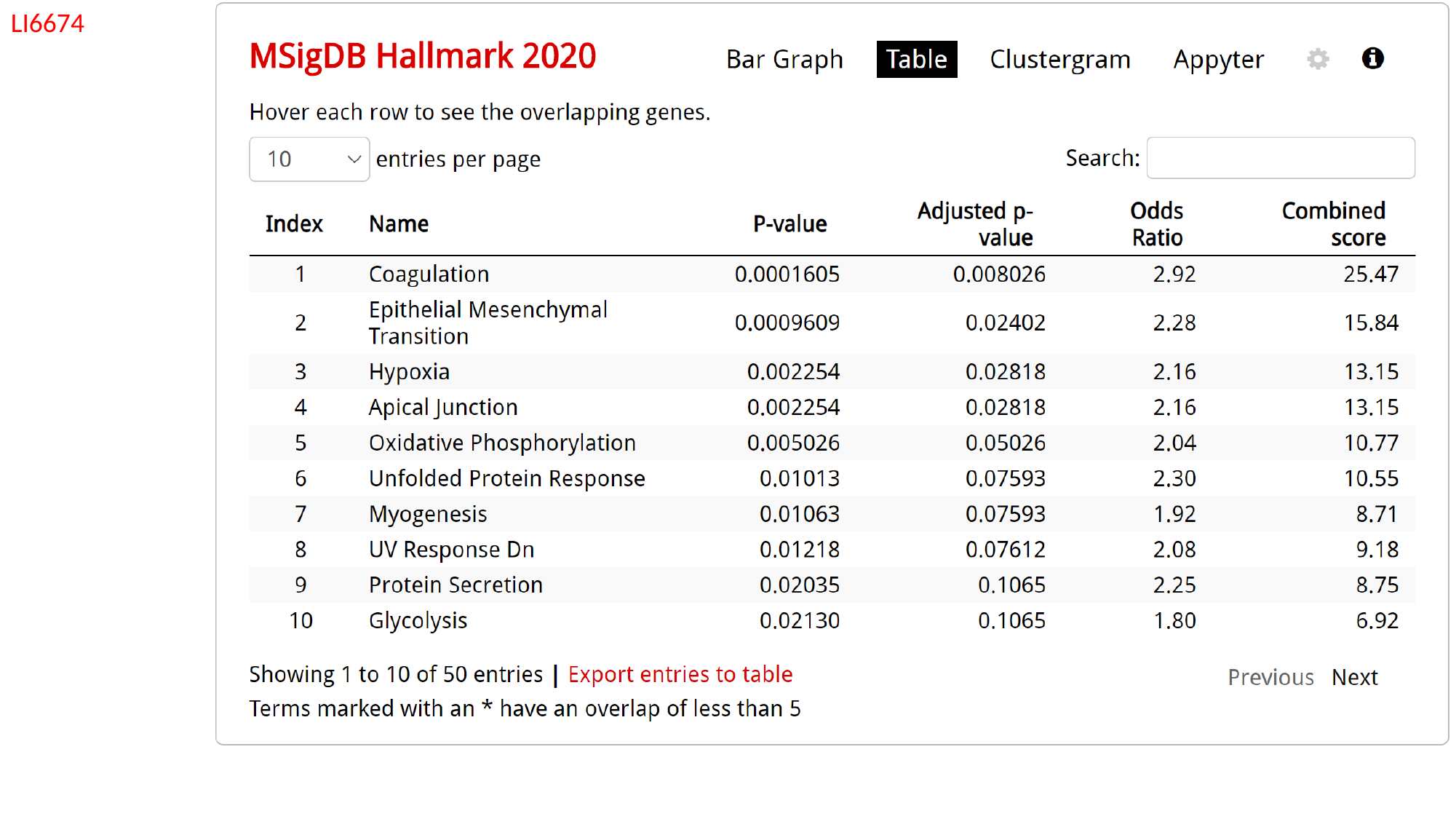

LI6674

## Slide 42
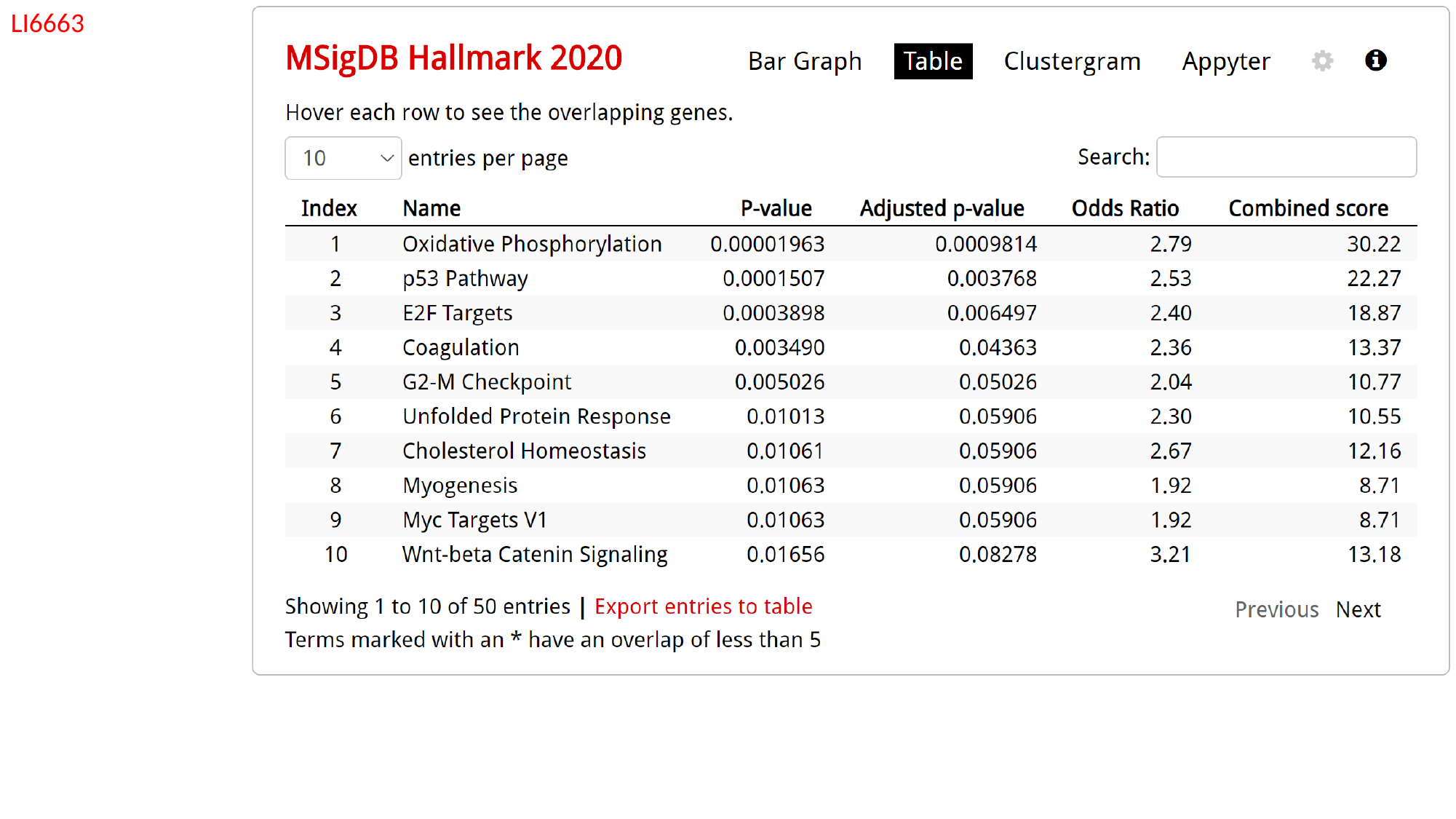

LI6663

## Slide 43
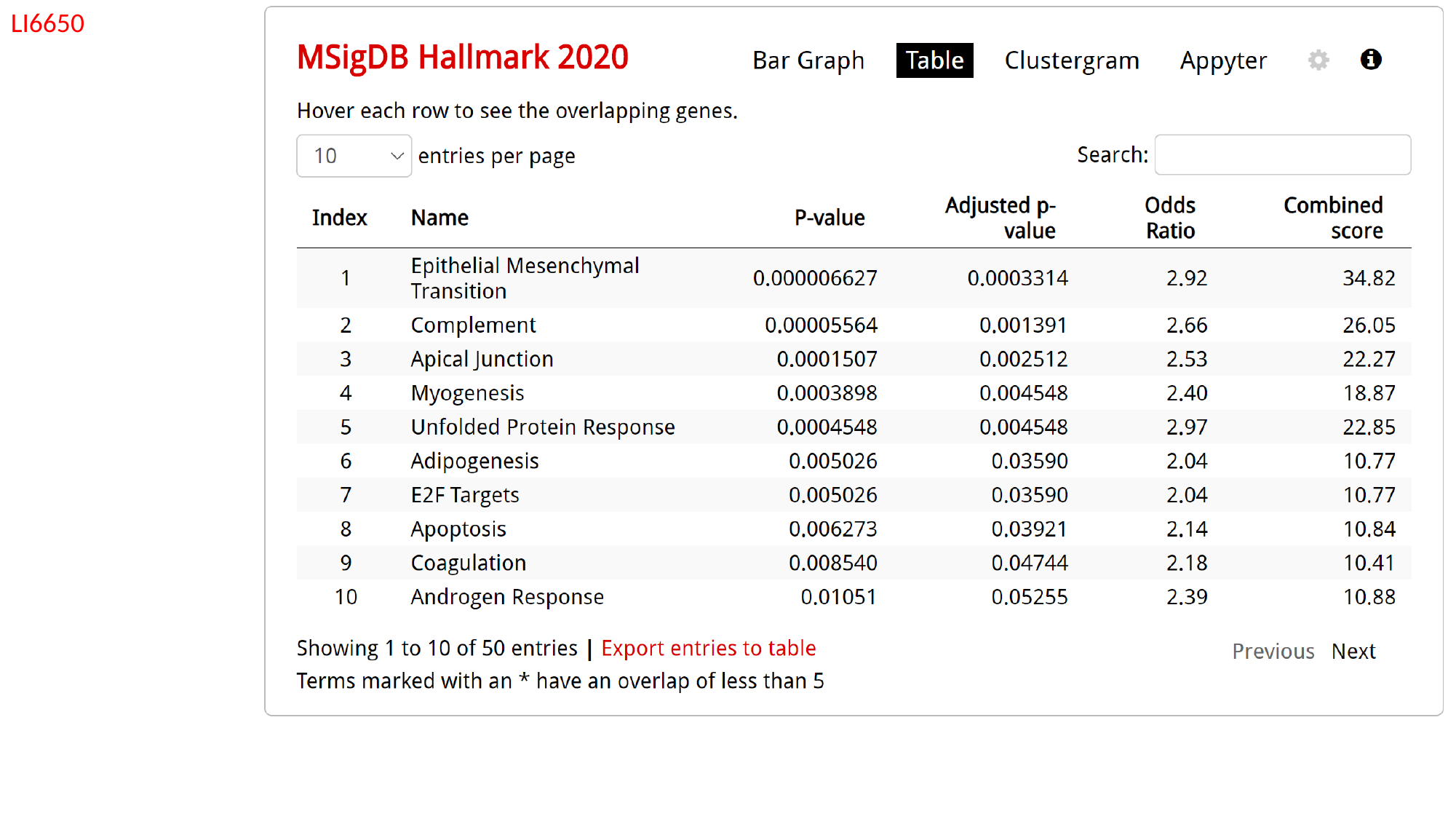

LI6650

## Slide 44
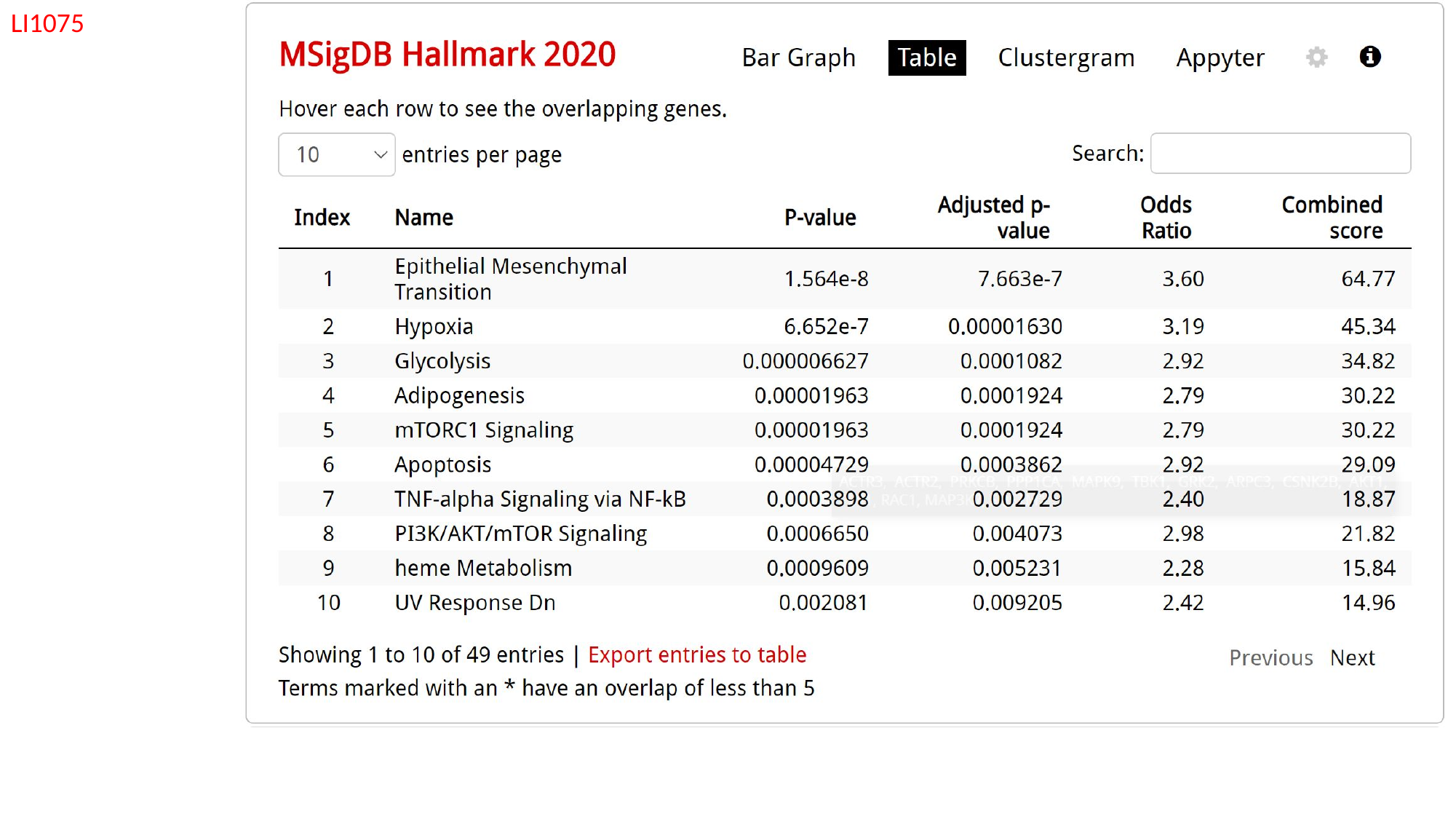

LI1075

## Slide 45
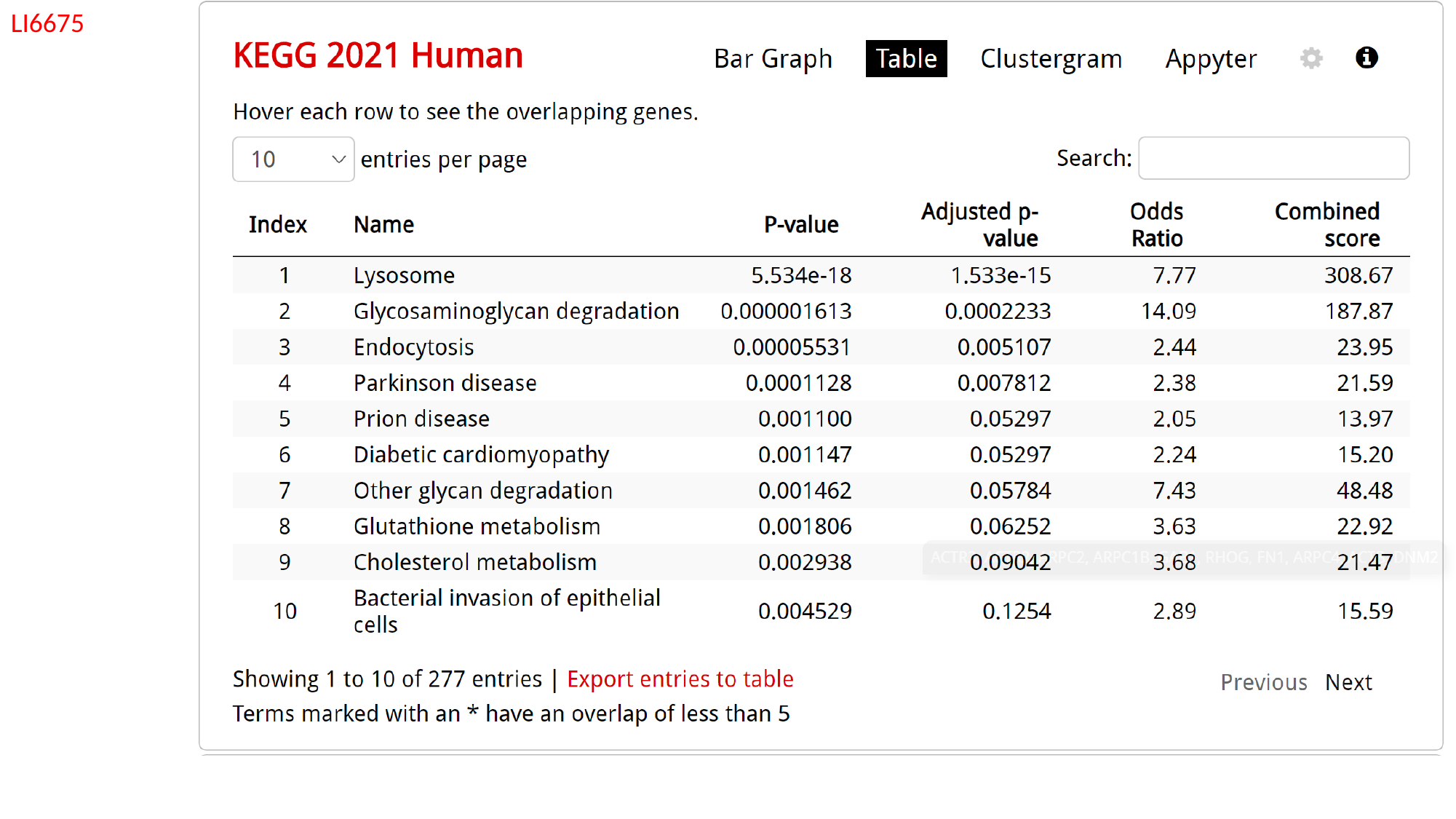

LI6675

## Slide 46
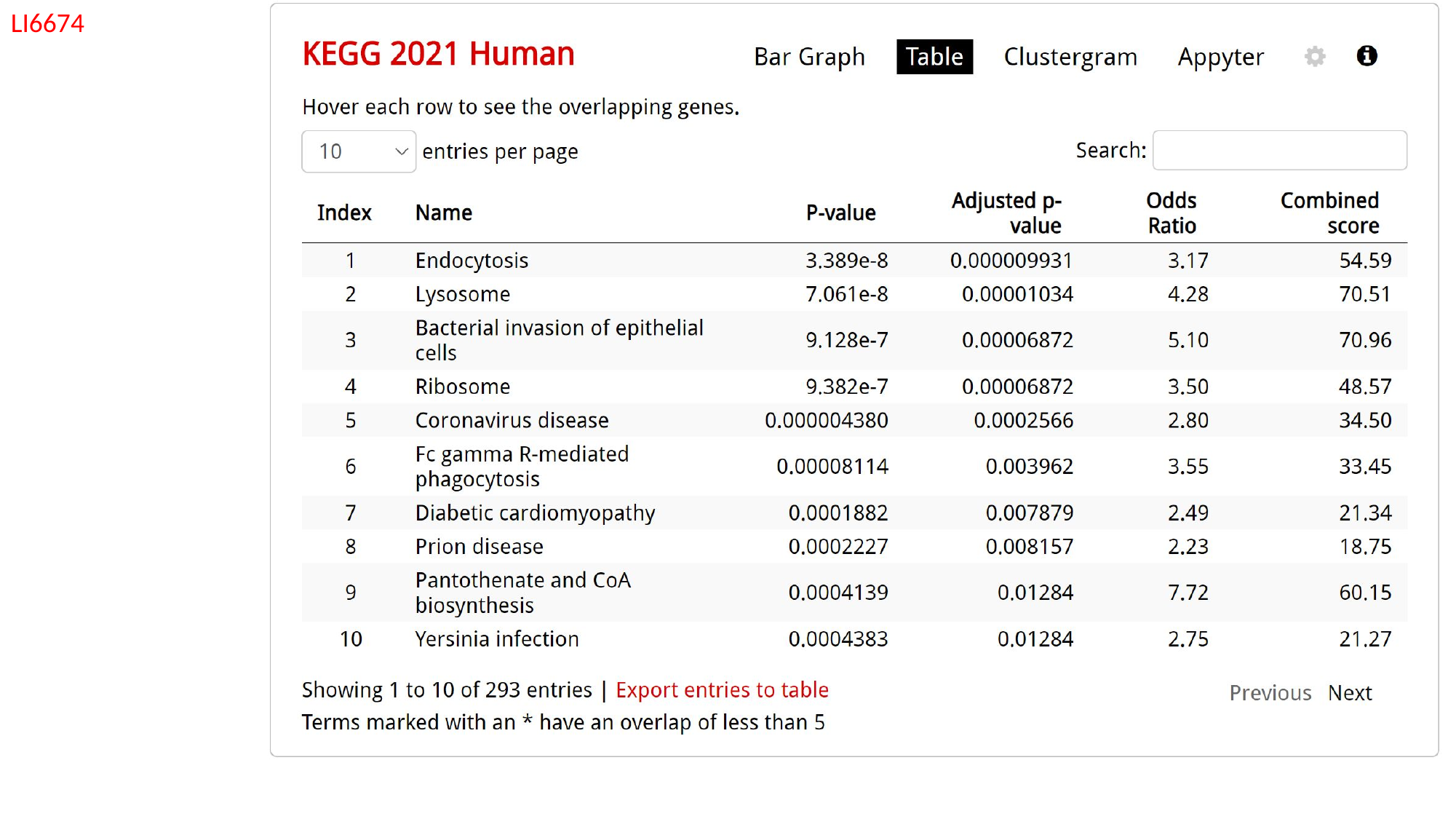

LI6674

## Slide 47
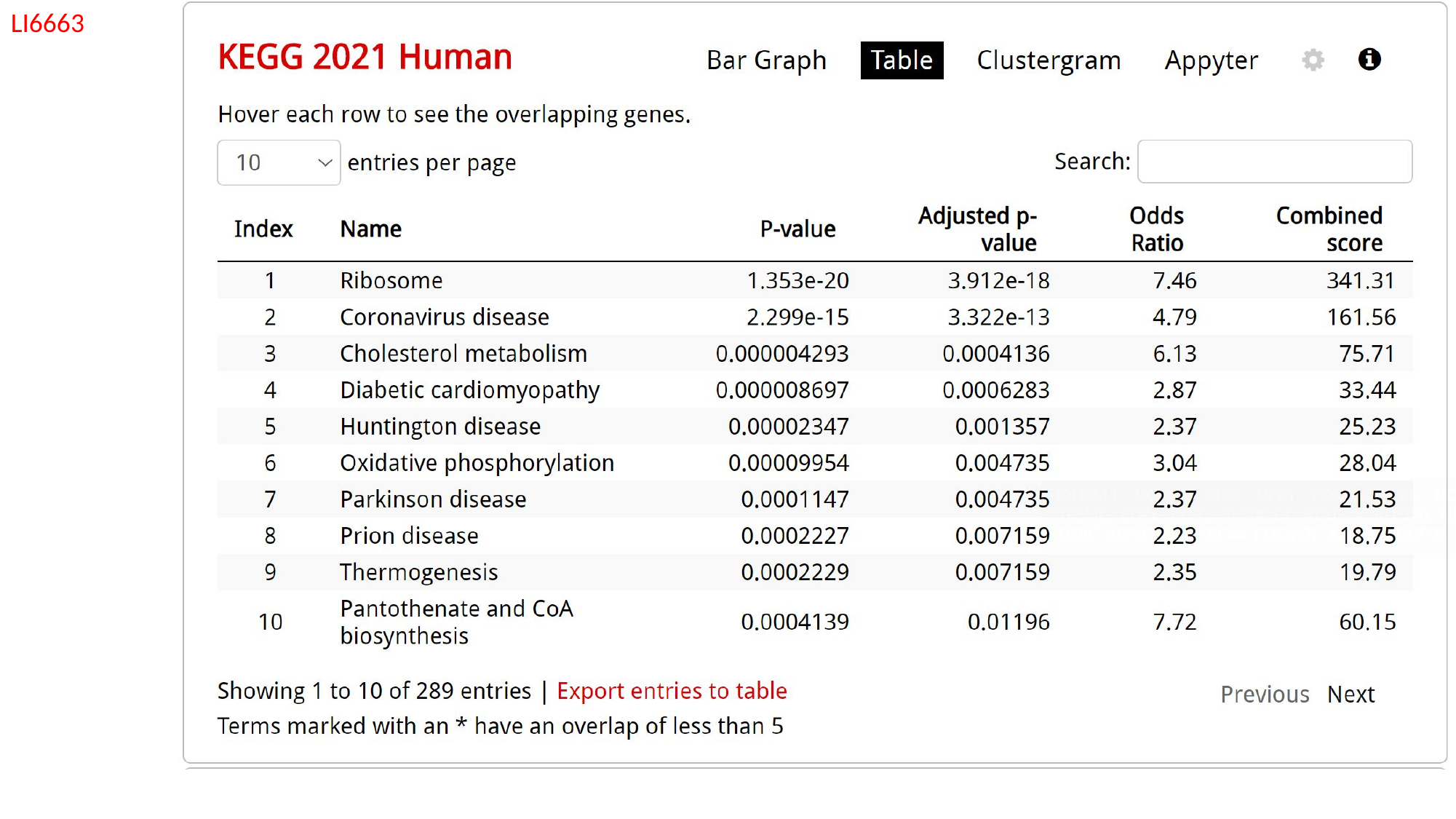

LI6663

## Slide 48
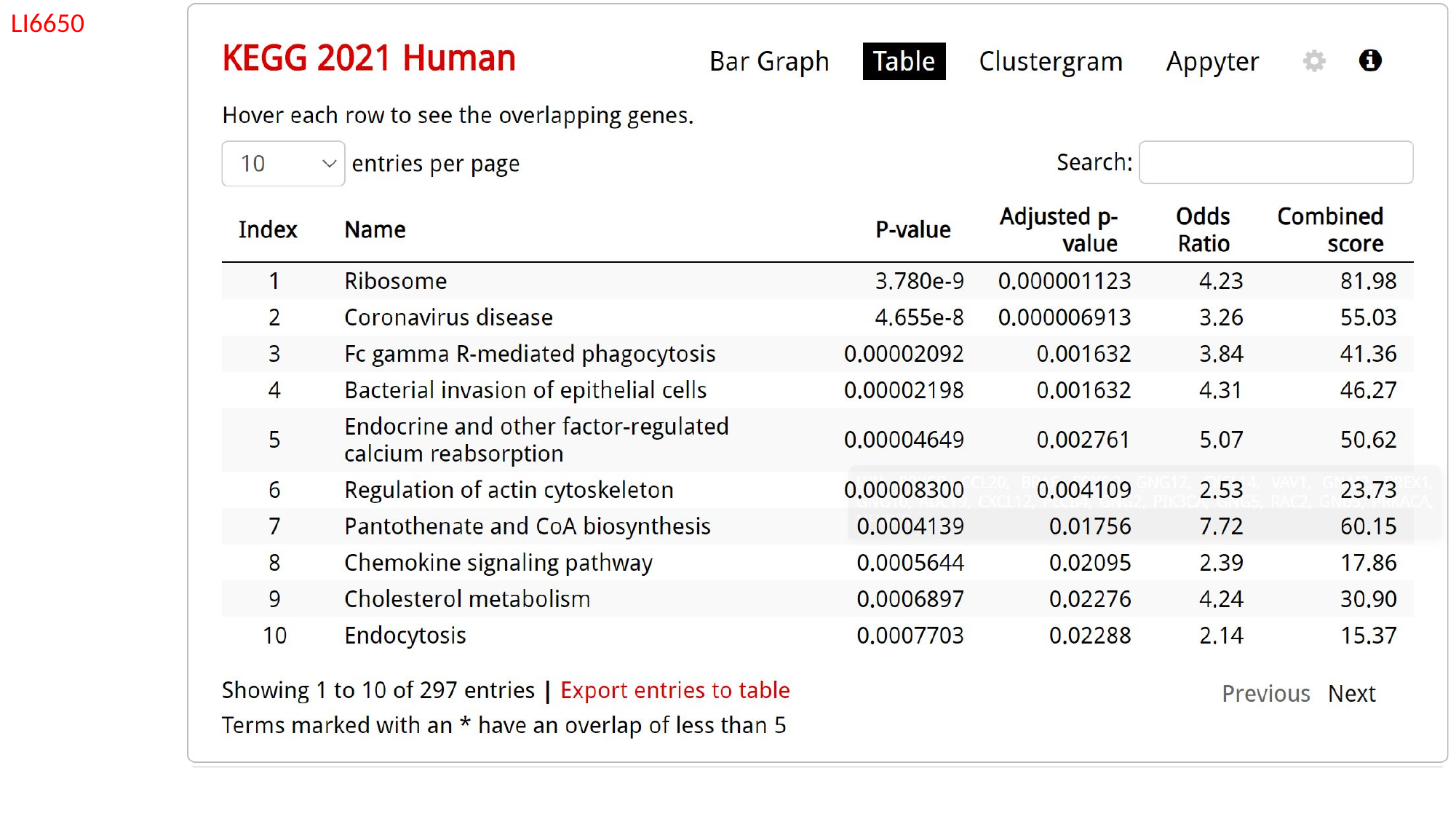

LI6650

## Slide 49
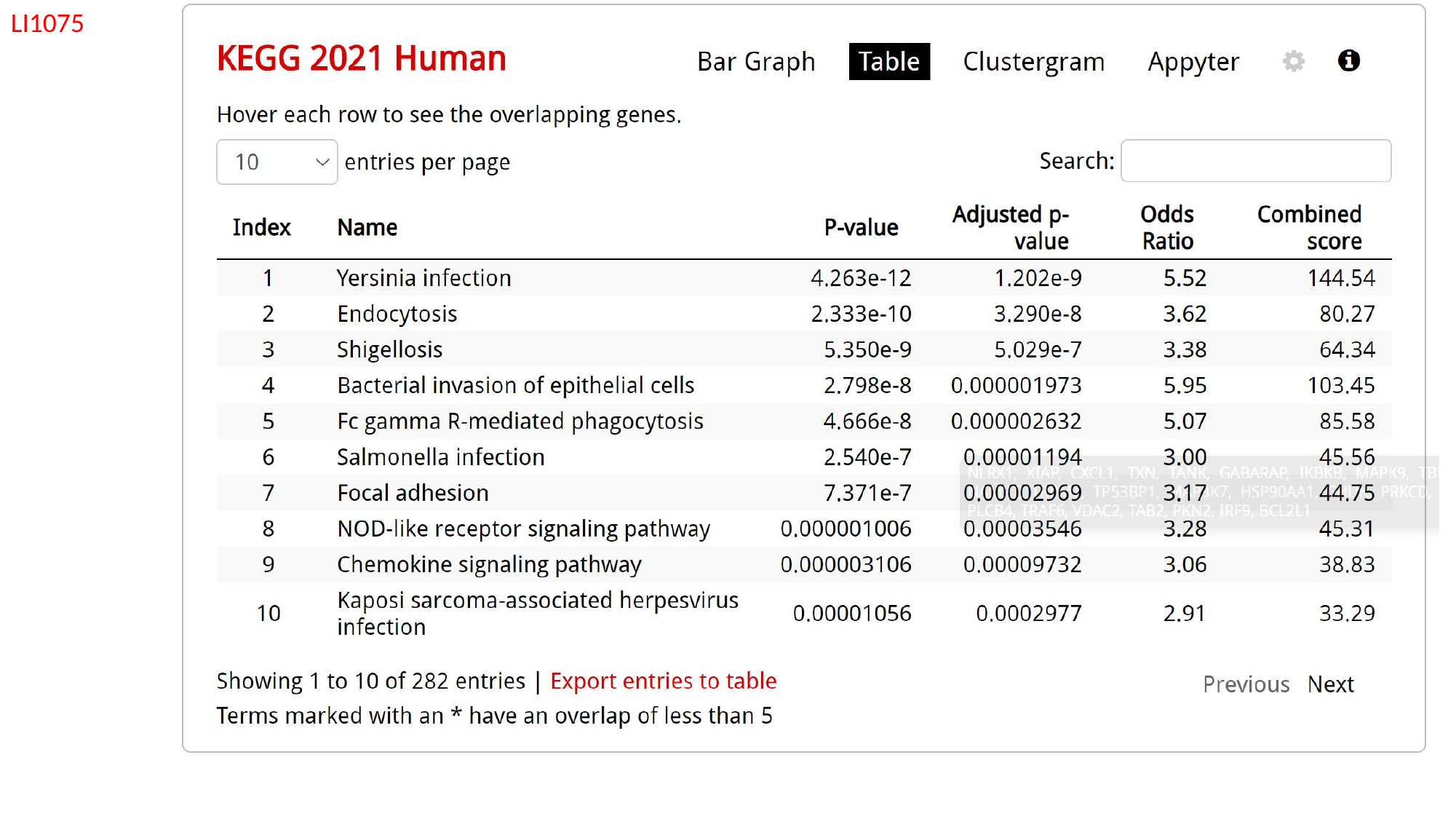

LI1075
